# Active Packaging, Fruit Volatilome, and the Preservation of Taste and Aroma Quality in Fresh Fruits: A Review

**DOI:** 10.3390/foods15183299

**Published:** 2026-09-17

**Authors:** Pedro Melgarejo-Martínez, Antonio López-Gómez, José Ramón Acosta-Motos, Ginés Benito Martínez-Hernández, Juan D. Franco-Navarro, Antonio José Pérez-López

**Affiliations:** 1 Eversia, Pol. Ind. El Saladar II, Calle Madrid s/n, 30564 Lorquí, Spain; pedro.melgarejo@eversia.es; 2 Food Safety and Refrigeration Engineering Group, Department of Agricultural Engineering, Universidad Politécnica de Cartagena, Paseo Alfonso XIII, 48, 30203 Cartagena, Spain; antonio.lopez@upct.es (A.L.-G.); ginesbenito.martinez@upct.es (G.B.M.-H.); 3 Institute of Plant Biotechnology, Universidad Politécnica de Cartagena, Campus Muralla del Mar, Edificio I+D+I, 30202 Cartagena, Spain; 4 Plant Biotechnology for Food and Agriculture Group (BioVegA^2^), Universidad Católica San Antonio de Murcia, Avenida de los Jerónimos 135, 30107 Guadalupe, Spain; juandediosfn@bioscriptsdb.com; 5 Plant Biotechnology, Agriculture and Climate Resilience Group, UCAM, Associated Unit to CSIC by CEBAS-CSIC, 30100 Murcia, Spain; 6 Plant Ion and Water Regulation Group, Instituto de Recursos Naturales y Agrobiología de Sevilla (IRNAS, CSIC), Av. de la Reina Mercedes, 10, 41012 Sevilla, Spain

**Keywords:** active packaging, fresh fruits, volatilome, aroma quality, taste, off-flavors, modified atmosphere packaging, sensory shelf life, consumer acceptance, odor thresholds

## Abstract

Fresh fruits are highly perishable products whose acceptance depends not only on external appearance and firmness, but also on the preservation of taste and aroma. This review addresses the relationship between active packaging and the maintenance of fruit sensory quality, with special emphasis on the volatilome as a dynamic marker of aroma integrity. The first part examines how taste and aroma are formed during ripening through the interaction of sugars, organic acids, phenolics, amino acids, and a wide range of volatile compounds, including fatty-acid-derived esters and aldehydes, terpenoids, carotenoid-derived norisoprenoids, phenylpropanoids, sulfur-containing compounds, and glycosidically bound precursors. It then analyzes how these traits deteriorate during packaged shelf life through temperature effects, refrigerated and room-temperature storage, microbial and enzymatic activity, mechanical damage, and packaging-atmosphere imbalance, showing that visual and sensory shelf life do not necessarily decline together. The second part reviews how active packaging can preserve—or unintentionally distort—this volatilome, covering modified-atmosphere and gas-regulating systems, antimicrobial and ethylene-managing systems based on released essential oils, sulfur dioxide and ethanol emitters, oxygen and carbon dioxide regulators, moisture absorbers, multi-active combinations, and packaging-adjacent edible coatings. A recurring principle emerges: every active function is double-edged, as an intervention that delays spoilage or senescence may itself add exogenous notes or trigger fermentative off-flavors unless it is tuned to a sensory compatibility threshold. The packaging material is also decisive, since paper and cardboard buffer humidity and interact weakly with aroma compounds, whereas high-barrier polymers sustain a modified atmosphere but can scalp esters and terpenes; conversely, releasing volatiles compatible with the native aroma can act as an aroma-positive strategy that restores characteristic notes. Active packaging should therefore be evaluated not only by conventional quality parameters, but also by headspace gas evolution, sugar/acid balance, odor-active volatiles, off-flavor markers, and sensory validation, together with consumer acceptance, odor-threshold relevance, migration and safety considerations, and kinetic matching between active-agent release, fruit respiration, and flavor evolution.

## 1. Introduction

Fresh fruits are valued not only as sources of nutrients, fiber, and bioactive compounds, but also as highly distinctive sensory products. Their market acceptance depends on appearance, texture, juiciness, sweetness, acidity, aroma, and the interaction among these attributes during consumption. Among them, flavor is especially complex because it does not arise from a single chemical group. Sugars and organic acids shape sweetness, sourness, and freshness, whereas volatile organic compounds largely determine the aromatic identity perceived before eating and retronasally during mastication. This interaction explains why fruits with similar soluble solids content or titratable acidity may be judged very differently by consumers if their volatile profile has been altered during breeding, ripening, cold storage, handling, or packaging [1,2,3].

In this context, the concept of fruit volatilome is particularly useful. For the purposes of this review, the fruit volatilome can be defined as the complete and dynamic set of volatile organic compounds emitted, accumulated, transformed, or lost by fruit tissues across development, ripening, postharvest storage, packaging, and consumption. This term is broader than a simple inventory of aroma compounds. It includes the biochemical origin of volatiles, their free and bound forms, their temporal evolution, their partitioning between fruit tissue and package headspace, and their sensory relevance. In fresh fruits, the volatilome is mainly alcohols, aldehydes, ketones, lactones, terpenoids, norisoprenoids, apocarotenoids, phenylpropanoids/benzenoids, and sulfur-containing compounds, although their relative abundance and odor activity vary greatly among species, cultivars, maturity stages, and storage conditions [4,5,6,7,8].

Aroma formation during ripening is the result of coordinated metabolic reprogramming. Fatty-acid-derived volatiles are produced through lipoxygenase-related pathways and include C6 aldehydes and alcohols associated with green and fresh notes, as well as esters that often contribute fruity and sweet-like aromas. Amino-acid-derived volatiles may provide branched-chain alcohols, aldehydes, esters, and sulfur compounds, which are relevant in fruits such as melon, banana, apple, and tropical species. Terpenoids, norisoprenoids, and apocarotenoids contribute floral, citrus, resinous, and fruity notes, depending on the fruit matrix. In addition, many volatiles occur as glycosidically bound precursors, which act as latent aroma reservoirs and can be released by enzymatic or physicochemical processes during ripening and storage [5,6,7,8].

The preservation of this volatilome is one of the major challenges in postharvest technology. Fresh fruits remain metabolically active after harvest, and their sensory profile continues to evolve during transport, storage, retail display, and domestic handling. Packaging can modify the fruit microenvironment by changing O_2_ and CO_2_ concentrations, water vapor pressure, ethylene accumulation, condensation, tissue dehydration, and microbial growth. These changes may be beneficial when they slow respiration, delay senescence, reduce decay, and limit mechanical or water-loss damage. However, an inappropriate package atmosphere can also suppress desirable volatile synthesis, promote fermentative metabolism, accumulate secondary volatiles, or generate sensory defects that reduce consumer acceptance even when the fruit remains visually acceptable [9,10,11,12,13].

The problem is especially relevant because visual shelf life and sensory shelf life do not always decline at the same rate. Low temperature is widely used to reduce respiration and microbial spoilage, but several fruits are sensitive to chilling-induced aroma loss. In tomato, refrigeration has been associated with reduced flavor-associated volatiles and altered expression of genes involved in volatile biosynthesis. In melon, postharvest chilling can reduce volatile acetate esters, which are compounds strongly associated with characteristic melon aroma. Conversely, room-temperature exposure during retail display may accelerate respiration, softening, sugar and acid changes, microbial growth, and the production of off-odors, particularly when temperature abuse interacts with restricted gas exchange inside the package [14,15,16,17]. Off-flavor formation in packaged fruits is commonly associated with fermentative metabolism, microbial spoilage, enzymatic degradation, tissue disruption, and excessive or poorly balanced atmosphere modification. Ethanol, acetaldehyde, ethyl acetate, organic acids, sulfur-related compounds, and other stress-associated volatiles can accumulate when oxygen availability becomes limiting or when microbial communities develop in damaged or high-moisture tissues. These compounds may contribute to alcoholic, solvent-like, vinegar-like, musty, sulfury, or stale notes. Importantly, some of these markers may appear before clear visual deterioration, which makes the volatilome a valuable early indicator of sensory quality loss [14,17].

Active packaging offers a promising strategy to manage postharvest deterioration because it is designed to interact with the food-package environment rather than acting only as a passive barrier. Depending on the system, active packaging may modulate O_2_ and CO_2_ concentrations, ethylene accumulation, moisture, microbial growth, oxidation reactions, or the controlled release of bioactive compounds. Its fundamental objectives include extending shelf life, improving safety, reducing decay, limiting water loss, and maintaining overall fruit quality. Nevertheless, in addition to these established functions, its sensory performance should also be evaluated by its capacity to preserve the native aroma and taste profile of the fruit, limit volatilome distortion, and avoid the formation of off-odors and off-flavors. Recent reviews on active, smart, and edible packaging have emphasized the expansion of antimicrobial, antioxidant, ethylene-scavenging, moisture-regulating, biodegradable, and intelligent systems, but the specific relationship between these technologies and the preservation of the fruit volatilome remains less systematically integrated [18,19].

Representative studies illustrate why this sensory perspective is necessary. In fresh-cut nectarines, active MAP affected quality, sensory attributes, and volatile profiles after cold storage, showing that packaging can be evaluated directly through aroma-relevant endpoints [20]. In strawberries, gas composition, condensation, and membrane permeability influenced off-odor development and volatile accumulation, demonstrating that visual quality preservation does not necessarily guarantee flavor preservation [12]. Similarly, a biopolymer-coated polyethylene active packaging system produced fruit-dependent responses: it helped reduce off-flavor production and preserve sesquiterpenes in longan, whereas in strawberry the volatile profile was more strongly conditioned by the package atmosphere, with hypoxic conditions increasing ethyl esters associated with off-flavor and reducing esters linked to typical fruity aroma [21]. Active volatile release may also be compatible with sensory preservation when properly controlled, as shown by active labels containing cinnamon essential oil in Calanda peach [22]. These examples support the need to distinguish between packages that preserve external quality and packages that genuinely preserve aroma, taste and consumer-relevant sensory identity.

This review examines the relationship between active packaging and the preservation of taste and aroma quality in fresh fruits, with special emphasis on the volatilome as a central marker of sensory integrity. The first part reviews how aroma and taste are formed during fruit ripening and how they deteriorate during packaged shelf life, considering temperature, refrigerated and room-temperature storage, microbial and enzymatic changes, mechanical damage, and packaging-atmosphere imbalance. The second part addresses the main packaging strategies involved in fruit sensory preservation, including modified-atmosphere and active gas-regulating systems, antimicrobial packaging, ethylene management, SO_2_ emission, multi-active approaches, ethanol emission, oxygen scavenging, carbon dioxide regulation, moisture control, and packaging-adjacent edible coatings. In addition, the review integrates consumer perception, sensory shelf life, odor-threshold relevance, aroma scalping, safety and migration issues, regulatory considerations, comparative effectiveness among active technologies and the kinetic matching between active-agent release, fruit respiration, and flavor evolution [23,24,25,26,27,28,29,30,31,32,33,34,35,36,37,38,39,40,41,42,43,44,45,46,47]. By connecting fruit physiology, volatile biochemistry, packaging design, and sensory quality, this review aims to support a more precise and critically validated evaluation of packaging technologies for fresh fruits.

## 2. Formation of Aroma and Taste in Fruits During Ripening

**Figure 1:**
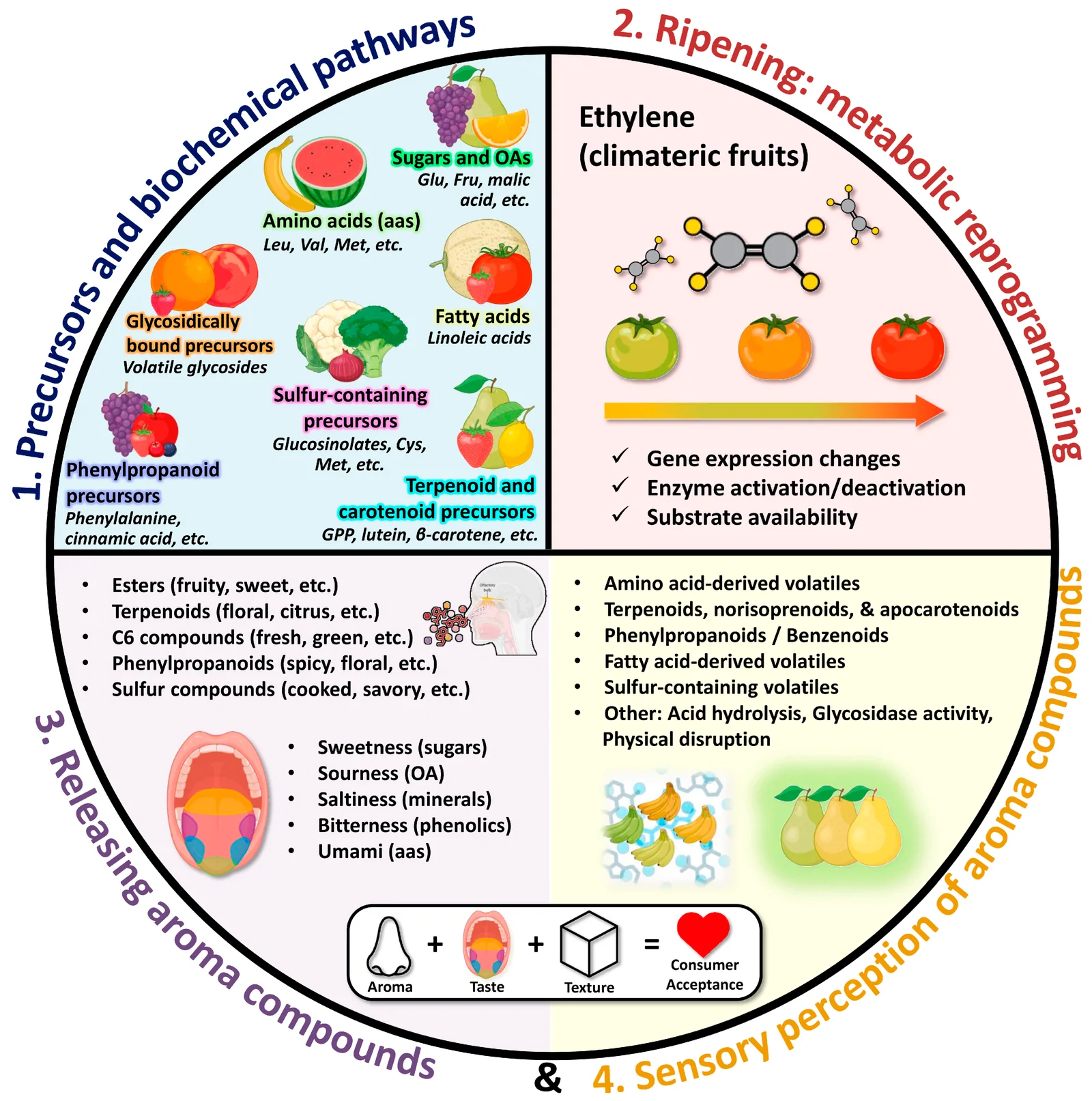
Schematic overview of the biochemical and physiological processes underlying aroma and taste development during fruit ripening. Primary metabolites and aroma precursors are transformed through ripening-associated metabolic reprogramming controlled by substrate availability, enzyme activation or deactivation, ethylene response, and gene expression changes. The figure distinguishes carbohydrate-derived taste components, fatty-acid-derived aldehydes, alcohols and esters, amino-acid-derived volatiles, terpenoid- and carotenoid-derived compounds, phenylpropanoid/benzenoid volatiles, sulfur-containing compounds, and glycosidically bound aroma precursors. The resulting volatilome, together with non-volatile taste-active compounds and fruit texture, shapes sensory perception and ultimately influences consumer acceptance. Abbreviations: OA, organic acids; GPP, geranyl diphosphate; C6 compounds, six-carbon volatile compounds; aas, amino acids.

Fruit ripening is the developmental process through which fleshy fruits acquire the sensory traits that define their commercial and eating quality. From a physiological perspective, ripening involves coordinated changes in respiration, hormone signaling, pigment metabolism, cell wall disassembly, primary metabolism, secondary metabolism, and tissue softening. From a sensory perspective, the same process builds the fruit’s flavor identity through the interaction between sweetness, acidity, bitterness, astringency, juiciness, texture and aroma. Sugars and organic acids provide the main taste framework, whereas volatile organic compounds are responsible for much of the characteristic aroma perceived before and during consumption [48,49,50]. This is particularly relevant for packaged fresh fruits because packaging technologies can preserve, modulate or damage an existing sensory potential, but they cannot fully create a volatilome that was not properly developed before harvest. Fruit harvested before adequate physiological maturity may retain firmness and external quality, but often shows weak aroma, insufficient sugar accumulation, excessive acidity, or an unbalanced volatile profile. Conversely, fruit harvested at a suitable maturity stage enters postharvest storage with a more complete biochemical capacity to sustain desirable volatile production, provided that temperature, atmosphere composition, and handling conditions do not suppress ripening-related metabolism [48,49,51,52]. The ripening-dependent formation of fruit sensory quality, from biochemical precursors and metabolic regulation to aroma release, taste perception, texture, and consumer acceptance is summarized in Figure 1.

At the molecular level, the formation of fruit flavor during ripening depends on coordinated regulation of precursor supply, structural genes, enzyme activities, and volatile release. Fatty-acid-derived aroma formation involves lipoxygenase-related metabolism and the downstream balance among aldehydes, alcohols, and esters through alcohol dehydrogenases and alcohol acyltransferases. Amino-acid-derived aroma depends on the catabolism of branched-chain and aromatic amino acids, whereas terpenoid and carotenoid-derived volatiles depend on terpene synthases and carotenoid cleavage. Ethylene regulates many of these processes in climacteric fruits by coordinating ripening-related transcription, substrate availability and enzyme activity, while non-climacteric fruits rely on a broader combination of hormonal and developmental signals. Therefore, active packaging can affect aroma not only by retaining or losing volatiles in the headspace, but also by modifying the physiological conditions that regulate the genes and enzymes responsible for volatile biosynthesis [51,52,53,54,55,56,57,58,59,60,61,62,63,64,65,66].

Ripening behavior differs markedly between climacteric and non-climacteric fruits, although this distinction should not be applied in an overly rigid manner. Climacteric fruits, including apples, banana, pear, peach, tomato, and several melon types, show a ripening-associated increase in respiration and ethylene production, and ethylene regulates many processes involved in softening, pigmentation, sugar/acid balance, and volatile biosynthesis. Non-climacteric fruits, including strawberry, grape, citrus, and many berries, do not show a climacteric ethylene burst, although ethylene may still influence specific ripening responses. In these fruits, abscisic acid, sugars, auxins, jasmonates and other signals contribute to ripening control in a species-dependent manner [51,52,67]. These physiological differences have direct postharvest implications. In climacteric fruits, part of the aroma-forming capacity may continue after harvest if the fruit has reached the appropriate physiological stage, whereas in many non-climacteric fruits the scope for postharvest aroma development is more limited. This explains why early harvesting may be especially detrimental for fruits such as strawberry, grape, or citrus, where on-plant ripening strongly determines sugar accumulation, acid decline, and varietal aroma. Nevertheless, cultivar, maturity stage, ethylene sensitivity, storage temperature and package atmosphere can strongly alter the final sensory output within the same species [48,52,67].

Taste development during ripening is largely determined by the accumulation and balance of soluble sugars, organic acids, phenolic compounds, free amino acids, and other soluble metabolites. Glucose, fructose, and sucrose are the main sugars contributing to sweetness, although their relative proportions vary among species and cultivars. Organic acids, mainly malic and citric acids, provide sourness and freshness and strongly modulate perceived sweetness. For this reason, the sugar/acid ratio is usually more informative than soluble solids content alone, especially in fruits such as citrus, pineapple, grape, apple, peach, and strawberry, where consumer acceptance depends on balance rather than sweetness alone [48,68,69]. Phenolic compounds influence bitterness, astringency, and mouthfeel, particularly in fruits such as grape, persimmon, pomegranate, apple peel, and berries. Free amino acids may contribute directly to taste, but their main relevance in the context of the volatilome is that they also act as precursors of aroma volatiles. For example, branched-chain and aromatic amino acids can generate aldehydes, alcohols, and esters with fruity, malty, floral, or honey-like notes, linking primary metabolism with the formation of fruit aroma [53,54,70].

The fruit volatilome can be understood as the complete and dynamic set of volatile organic compounds produced, stored, transformed, released, or lost by fruit tissues during development, ripening, and postharvest life. In ripe fruits, this volatilome is commonly composed of esters, alcohols, aldehydes, ketones, lactones, terpenes, sesquiterpenes, norisoprenoids, apocarotenoids, phenylpropanoids, benzenoids, and sulfur-containing compounds [4,5,6,7,53,54,70]. The sensory relevance of each compound depends not only on its concentration, but also on odor threshold, volatility, matrix interactions, tissue disruption, temperature, and retronasal release during consumption. Therefore, compounds present at low concentrations may strongly influence aroma if their odor thresholds are low, whereas abundant compounds may have limited sensory impact if they are poorly odor-active. This is why the preservation of fruit aroma during packaging should not be evaluated only through total volatile abundance, but through the maintenance of key odor-active compounds and the avoidance of off-flavor markers [3,14,48,69].

Fatty-acid metabolism is one of the main biochemical sources of fruit aroma volatiles. Through lipoxygenase-related pathways, linoleic and linolenic acids can be converted into C6 and C9 aldehydes, alcohols, and esters. Compounds such as hexanal, (E)-2-hexenal, (Z)-3-hexenal, hexanol, and (Z)-3-hexenol are associated with green, grassy, leafy, and fresh-cut notes. In moderate concentrations, these volatiles contribute freshness; in excess, they may indicate immaturity, mechanical damage, or tissue disruption. Their formation is therefore closely linked to membrane status, oxygen availability, enzyme activity, and storage conditions [53,54,55,56]. As ripening progresses, many fruits shift from aldehyde-rich profiles towards alcohols and esters. Alcohol dehydrogenases participate in the interconversion between aldehydes and alcohols, while alcohol acyltransferases catalyze ester formation from alcohols and acyl-CoA substrates. Esters are especially important in apple, banana, strawberry, melon, and pear, where they provide fruity, sweet, banana-like, apple-like, pear-like or tropical notes. This pathway is highly relevant for packaging because excessive atmosphere restriction, low oxygen, altered ethylene action or unsuitable temperature may reduce desirable ester biosynthesis or redirect metabolism towards fermentative compounds [56,57,58,59,60].

Amino-acid-derived volatiles represent another major component of fruit aroma. Branched-chain amino acids such as leucine, isoleucine and valine may generate branched-chain aldehydes, alcohols, and esters, whereas phenylalanine may lead to phenylacetaldehyde, 2-phenylethanol, benzaldehyde, methyl benzoate, eugenol, and related benzenoid or phenylpropanoid compounds. These volatiles contribute fruity, malty, floral, honey-like, almond-like, or spicy notes depending on their concentration and fruit matrix [53,61,62,70]. Methionine and cysteine can also generate sulfur-containing compounds. These compounds are often present at low concentrations and may contribute to desirable tropical or varietal notes when they remain within an appropriate sensory range. However, under unsuitable storage or packaging conditions, especially when oxygen availability is restricted or microbial activity increases, sulfur-containing volatiles may shift towards sulfury, cabbage-like, onion-like, or overripe notes [14,17,62,70].

Terpenoids, carotenoid-derived volatiles, and phenylpropanoid/benzenoid compounds also contribute strongly to fruit aroma identity. Monoterpenes such as limonene, linalool, geraniol, alpha-terpineol, and citral provide citrus-like, floral, fresh, and resinous notes in fruits such as citrus, grape, mango, melon, and berries. Carotenoid cleavage generates apocarotenoids and norisoprenoids, including beta-ionone, beta-damascenone, and geranylacetone, which can have a strong sensory impact at very low concentrations [53,54,63,64]. These pathways connect aroma with ripening-associated pigment metabolism, explaining why changes in fruit color and volatile composition may be metabolically related. Phenylpropanoid and benzenoid volatiles, including benzaldehyde, benzyl alcohol, methyl benzoate, eugenol, guaiacol, methyl salicylate, phenylacetaldehyde, and 2-phenylethanol, can provide almond-like, floral, clove-like, smoky, medicinal, spicy, or honey-like notes. Their contribution is strongly species- and cultivar-dependent, and studies in strawberry have shown how domestication and breeding can reshape the gain or loss of specific flavor-related compounds [65,69].

Not all aroma compounds occur exclusively as free volatiles. Many fruits contain glycosidically bound aroma precursors, in which volatile aglycones are conjugated to sugar residues. These bound forms are usually non-volatile or weakly volatile and therefore do not directly contribute to aroma until hydrolysis releases the aglycone. This bound fraction acts as a latent aroma reservoir and has been described in grape, mango, citrus, kiwifruit, tamarillo, strawberry, and other fruits [8,66]. Its relevance for packaged fruits is considerable because tissue integrity, endogenous enzymes, microbial enzymes, pH, and atmosphere composition may influence the release or transformation of bound aroma compounds during shelf life. A package that delays senescence but suppresses the release of desirable aroma aglycones may preserve firmness while weakening flavor, whereas conditions that promote tissue stress or microbial hydrolysis may accelerate the formation of atypical aroma notes [8,14,66].

The sensory potential established during ripening remains highly vulnerable after harvest because the same metabolic pathways responsible for desirable aroma can also shift towards quality loss under unsuitable storage or packaging conditions. Fatty-acid oxidation, altered ester biosynthesis, amino-acid catabolism, carotenoid cleavage, glycoside hydrolysis, and fermentative metabolism may all modify the volatile profile once fruit tissues are exposed to cold stress, temperature abuse, restricted gas exchange, mechanical injury, or microbial development [14,17,53,54,55,56,61,66]. The transition from ripening to shelf life should therefore be understood as a continuum rather than as two independent phases: the volatilome formed during ripening becomes the biochemical substrate on which postharvest deterioration processes act. This point is particularly relevant for packaged fresh fruits, where the package atmosphere may either preserve the native aroma profile or accelerate the appearance of off-odors and off-flavors depending on the balance between fruit respiration, film permeability, temperature, and microbial control [9,10,11,12,13,14].

Maturity stage should therefore be considered a primary determinant of packaging response. In immature or early-harvested fruits, the native volatilome may be poorly developed; active packaging can maintain firmness and external appearance, but it cannot fully generate the aroma potential that was not established during ripening. In mature-firm climacteric fruits, carefully designed packaging may slow softening and water loss while still allowing limited ripening-related volatile formation during distribution, but excessive ethylene removal, low O_2_, or high CO_2_ may suppress the formation of esters, lactones and other ripe-fruit volatiles. In ready-to-eat fruits, the packaging objective is different: the priority is to preserve an already developed aroma profile, avoid aroma scalping, limit fermentative markers, and prevent microbial off-odors without excessively restricting volatile release. In overripe or senescent fruits, packaging may delay visible decay or dehydration, but it is unlikely to restore the native aroma once tissue breakdown, fermentation, or microbial metabolism dominate. For non-climacteric fruits such as strawberry, grape, citrus, and many berries, harvest maturity is especially decisive because postharvest aroma development is more limited than in climacteric fruits; therefore, packaging should be evaluated as a preservation tool rather than as a substitute for appropriate maturity at harvest [48,52,67]. Overall, the preservation of fruit sensory quality requires consideration of both the biochemical origin of taste and aroma and the variables used to evaluate their evolution during packaged storage. The main biochemical routes involved in the formation of taste- and aroma-related compounds during fruit ripening are summarized in Table 1, whereas Table 2 integrates conventional quality measurements with volatilome-related variables that should be considered when evaluating the performance of active packaging for fresh fruits. 

## 3. Taste and Aroma Deterioration Processes During the Shelf Life of Packaged Fresh Fruits

After harvest, fresh fruits remain metabolically active, and their sensory quality continues to evolve during storage, transport, retail display, and domestic handling. In packaged fruits, this evolution is shaped by the interaction between fruit physiology and the microenvironment created by packaging. Respiration, transpiration, ethylene production, tissue softening, membrane changes, and microbial development continue after harvest, while the package modifies oxygen availability, carbon dioxide accumulation, water vapor pressure, condensation, headspace volume and volatile partitioning [11,12,13,14]. As a result, shelf life should not be interpreted only as the period during which fruit remains visually acceptable, but as the period during which appearance, texture, taste, and aroma remain aligned with consumer expectations. Recent reviews on postharvest flavor deterioration have emphasized that odor-active volatile changes, sugar/acid imbalance, fermentative metabolism and microbial spoilage may compromise flavor before severe external symptoms become evident [14,72].

Taste deterioration during packaged storage is mainly associated with changes in soluble sugars, organic acids, phenolic compounds, and texture-related perception. Sugars and organic acids may be consumed as respiratory substrates, diluted by water movement, modified by continued ripening or affected indirectly by senescence and tissue breakdown. These changes alter sweetness, sourness, and freshness, but their sensory impact also depends on aroma because some volatiles can enhance or suppress perceived sweetness and fruitiness [1,48,68,69]. Phenolic oxidation, cell wall degradation, and loss of juiciness may further change mouthfeel, bitterness, astringency and the release of taste-active compounds during mastication. Therefore, taste deterioration in packaged fruits is not only a chemical process, but also a physiological and structural process in which metabolism, texture, and aroma release interact [71,73].

Aroma deterioration is more directly linked to changes in fruit volatilome. During shelf life, desirable odor-active compounds may decrease because of reduced biosynthesis, volatilization, enzymatic transformation, sorption into packaging materials or altered partitioning between tissue and headspace. At the same time, new compounds may accumulate through lipid oxidation, amino-acid catabolism, fermentative metabolism, tissue disruption, or microbial activity [14,17,53,54]. This dual process is central to packaged fruit quality: sensory failure may result either from the loss of characteristic varietal volatiles or from the appearance of off-odor markers such as ethanol, acetaldehyde, ethyl acetate, acetic acid, sulfur-like volatiles, musty compounds, or stale aldehydic notes [14,17,72].

The deterioration trajectory is strongly influenced by the design of the packaging system. A suitable package can slow respiration, reduce water loss, limit microbial growth and maintain a favorable O_2_/CO_2_ balance. However, a poorly matched package can suppress desirable aroma biosynthesis, promote hypoxia, accelerate fermentative pathways, increase condensation, or create conditions that favor microbial off-odor formation [11,12,13]. This is especially relevant for fresh-cut fruits, berries, and soft fruits because their high-water activity, fragile tissues, and exposed surfaces increase respiration, leakage of cellular nutrients and susceptibility to microbial spoilage [12,71]. Thus, the preservation of taste and aroma requires packaging systems that are evaluated not only through firmness, color, weight loss and decay incidence, but also through odor-active volatile preservation, off-flavor prevention, and maintenance of the sugar/acid balance [13,14,72].

**Figure 2:**
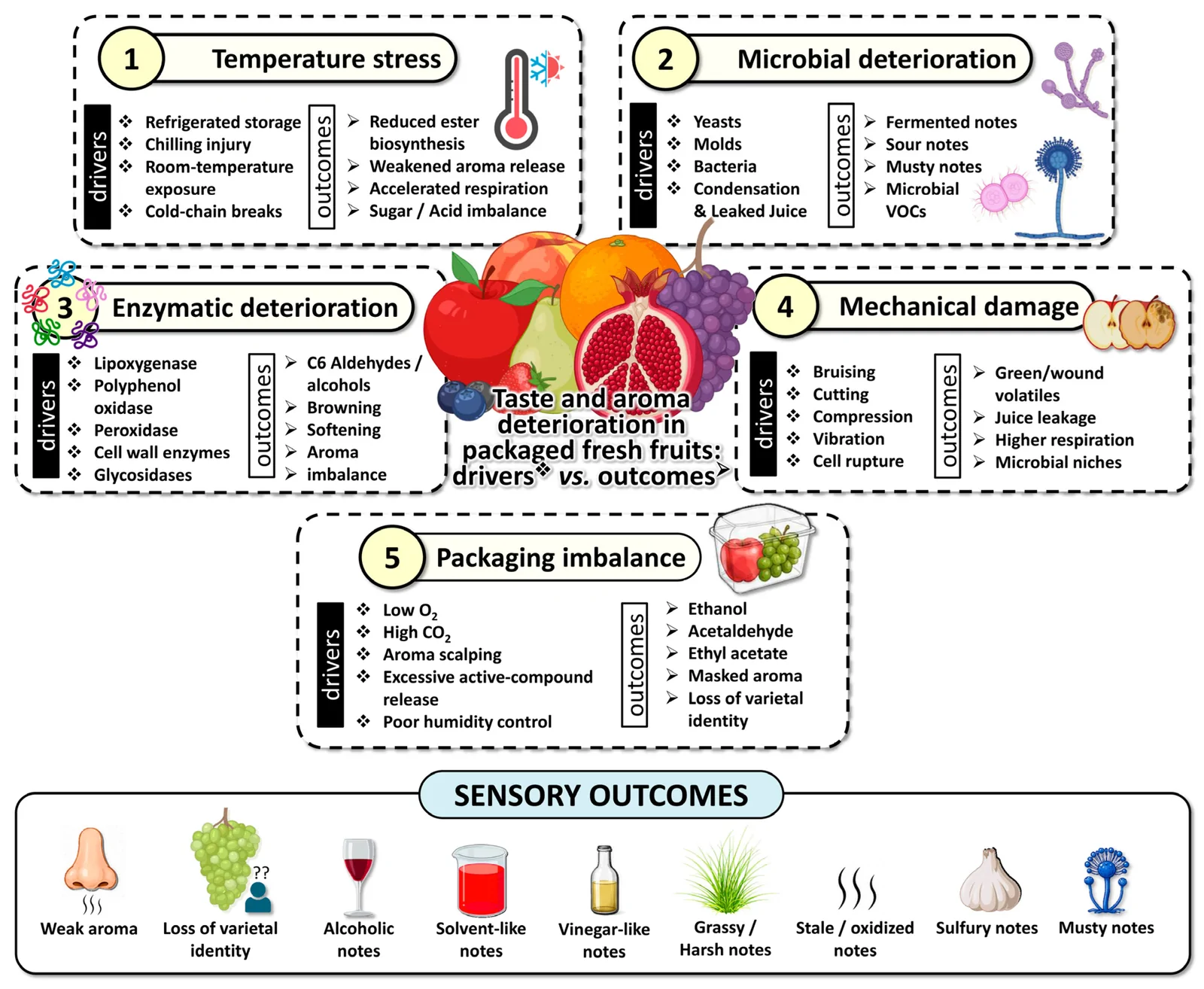
Main routes of taste and aroma deterioration during the shelf life of packaged fresh fruits. Temperature stress, microbial deterioration, enzymatic deterioration, mechanical damage, and packaging imbalance can independently or jointly alter the sensory quality of packaged fresh fruits. Temperature stress includes refrigerated storage, chilling injury, room-temperature exposure and cold-chain breaks, which may reduce ester biosynthesis, weaken aroma release, accelerate respiration and disturb the sugar/acid balance. Microbial deterioration, driven by yeasts, molds, bacteria, condensation, and leaked juice, may generate fermented, sour, musty notes, and microbial volatile organic compounds. Enzymatic deterioration involves lipoxygenase, polyphenol oxidase, peroxidase, cell wall enzymes, and glycosidases, leading to C6 aldehydes and alcohols, browning, softening, and aroma imbalance. Mechanical damage caused by bruising, cutting, compression, vibration or cell rupture promotes wound-related volatiles, juice leakage, increased respiration and microbial niches. Packaging imbalance, including low O_2_, high CO_2_, aroma scalping, excessive active-compound release, and poor humidity control, may favor ethanol, acetaldehyde, ethyl acetate accumulation, masked aroma, and loss of varietal identity. The final sensory outcomes include weak aroma, loss of varietal character, alcoholic, solvent-like, vinegar-like, grassy, musty, sulfury, stale, or oxidized notes, and reduced consumer acceptance.

In this section, deterioration is considered through two connected levels. First, temperature is addressed as the main external driver controlling respiration, ethylene response, enzyme activity, microbial proliferation and volatile release. Second, the formation of off-odors and off-flavors is examined through microbial, enzymatic, mechanical, and packaging-related routes. This structure is useful because the same sensory defect may arise from different mechanisms. For example, alcoholic notes may reflect fruit fermentation under low oxygen, yeast activity in damaged tissues, or excessive atmosphere restriction inside the package; green notes may indicate normal freshness, immaturity, cutting injury, or lipid oxidation depending on concentration and context [14,17,53,72]. A mechanistic interpretation of these routes is therefore essential for designing active packaging systems that preserve the native fruit volatilome rather than merely prolonging visual shelf life. The main physiological, microbiological, enzymatic, mechanical and packaging-related routes involved in taste and aroma deterioration during packaged shelf life are summarized in Figure 2.

### 3.1. Influence of Temperature on Taste and Aroma Deterioration in Fresh Fruit

Temperature is one of the most decisive external factors controlling the deterioration of taste and aroma in fresh fruits because it simultaneously affects fruit metabolism, microbial growth, gas exchange, and volatile release. As temperature increases, respiration and ethylene-related processes generally accelerate, leading to faster consumption of sugars and organic acids, more rapid softening, increased membrane permeability, and shorter sensory shelf life. Conversely, low temperature slows many of these processes and is therefore essential for postharvest preservation, but excessive cooling may impair ripening-related aroma biosynthesis or induce chilling injury in sensitive fruits [14,48,72,74]. This dual role makes temperature management particularly complex: the optimum condition is not simply the lowest possible temperature, but the lowest temperature that delays deterioration without suppressing the fruit’s capacity to maintain its characteristic sensory profile.

The effect of temperature on the volatilome depends on both biochemical and physical mechanisms. Biochemically, temperature modifies the activity of enzymes involved in volatile formation, including lipoxygenases, alcohol dehydrogenases, alcohol acyltransferases, terpene synthases, and enzymes related to amino-acid and carotenoid-derived volatile metabolism [53,54,55,56,57,58,59,60,61,62]. Physically, temperature affects the vapor pressure of volatile compounds and their partitioning between fruit tissue, juice phase, cut surface, package atmosphere, and the surrounding air. A fruit stored at low temperature may contain relevant aroma compounds but release them less intensely at the moment of consumption, whereas a fruit exposed to warmer conditions may emit volatiles more strongly while simultaneously deteriorating faster [14,48,74]. Thus, aroma intensity during shelf life reflects both the metabolic preservation of the volatilome and the temperature-dependent release of volatile compounds.

Temperature also determines whether packaging produces a beneficial modified atmosphere or an unfavorable microenvironment. In packaged fruits, O_2_ concentration decreases and CO_2_ concentration increases as a function of fruit respiration, film permeability, headspace volume, product load and storage temperature. If temperature rises above the range for which the package was designed, respiration may exceed gas transmission through the film, causing excessive O_2_ depletion and CO_2_ accumulation. Under these conditions, fruit tissues may shift towards fermentative metabolism, with increased production of ethanol, acetaldehyde, ethyl acetate, and other compounds associated with alcoholic, solvent-like, or overripe notes [11,17,75,76]. For this reason, temperature abuse is especially problematic in packaged fruits: it not only accelerates normal senescence but can also transform a suitable packaging atmosphere into a sensory-damaging one.

The interaction between temperature and packaging is particularly relevant for fresh-cut fruits, berries, and other highly perishable commodities. Cutting or tissue damage increases respiration, surface area, cellular leakage, and microbial susceptibility, while packaging increases humidity and modifies gas composition. At inadequate temperatures, this combination can accelerate sugar and acid depletion, phenolic oxidation, tissue softening, microbial growth and off-odor formation [71,72,75]. In these products, a short period of temperature abuse may have a disproportionate impact because the exposed tissues respond rapidly and because the package atmosphere can change faster than in intact fruits. Therefore, the temperature sensitivity of the product should be considered together with the permeability and active functions of the package, rather than as an independent storage variable [75,76].

From a sensory perspective, temperature influences both the rate and the direction of quality change. Moderate warming may temporarily increase aroma perception by enhancing volatile release, but it can also accelerate the transition from desirable ripening notes to overripe, fermented, or stale notes. Excessive cooling may preserve firmness and external appearance, but weaken aroma by reducing ester formation, altering ethylene-dependent metabolism, or inducing chilling-related physiological disorders in sensitive fruits [14,15,16,74]. This explains why visual quality is often an insufficient indicator of sensory quality in packaged fruit. A fruit may remain firm, bright, and apparently marketable while its volatilome has already lost key odor-active compounds or accumulated early off-flavor markers [12,13,14].

For active packaging, temperature should therefore be treated as a design parameter, not only as a storage condition. Oxygen scavengers, CO_2_ emitters or absorbers, ethylene scavengers, antimicrobial systems, moisture regulators, and volatile emitters may all show temperature-dependent kinetics. A system that performs well under stable refrigeration may be less effective, or even detrimental, under fluctuating retail or domestic conditions if its active response does not match fruit respiration and microbial dynamics [18,19,75,76]. Consequently, studies evaluating active packaging for fresh fruits should report not only nominal storage temperature, but also temperature fluctuations, headspace gas evolution, humidity, condensation, volatile profile, and sensory descriptors. Without this information, it is difficult to determine whether a packaging technology truly preserves fruit volatilome or merely prolongs external shelf life.

#### 3.1.1. Refrigerated Storage

Refrigerated storage is one of the most widely used strategies to extend the shelf life of fresh fruits because it reduces respiration, ethylene production, transpiration, enzymatic activity, and microbial growth. However, refrigeration does not always preserve sensory quality in the same way that it preserves external appearance. In many fruits, low temperature slows visible deterioration but may also reduce the formation or release of key aroma volatiles, especially esters, terpenoids, and other compounds associated with ripe-fruit identity [14,15,16,74]. This discrepancy is particularly important in packaged fruits because low temperature, package permeability, and fruit respiration jointly determine the headspace atmosphere. If refrigeration reduces volatile biosynthesis or release, the fruit may remain visually acceptable while its aroma becomes weaker, less varietal or less attractive to consumers [12,13,14,76]. The effect of refrigeration depends strongly on the physiological tolerance of each fruit species. Temperate fruits such as apples, pears, and many berries generally benefit from cold storage, although prolonged storage may still modify volatile profiles and reduce aroma intensity. By contrast, many tropical and subtropical fruits are susceptible to chilling injury when stored below their tolerance threshold but above the freezing point. Chilling injury involves membrane dysfunction, altered lipid metabolism, oxidative stress, impaired energy status, and disruption of ripening-related processes, which may lead to pitting, browning, uneven ripening, water-soaked tissues, mealiness, increased decay, and poor aroma development [77,78]. From the perspective of the volatilome, chilling injury is relevant because membrane damage and oxidative stress can alter fatty-acid-derived volatiles, suppress normal ester biosynthesis and favor the appearance of stress-related aldehydes, alcohols or stale notes [14,53,55,77].

Tomato provides a clear example of the sensory risks associated with refrigeration. Although low temperature can delay softening and decay, chilling has been linked to a marked loss of flavor-related volatiles and to changes in the expression and epigenetic regulation of genes involved in volatile synthesis [15]. This is highly relevant for packaged tomatoes and tomato-derived fresh products because the consumer often judges quality by aroma intensity, sweetness perception, and the balance between green and ripe notes. A package that maintains firmness but allows the cold-induced depletion of key volatiles would extend commercial shelf life without preserving eating quality [3,15,48,69]. Similar reasoning applies to aromatic melons, where postharvest chilling can reduce volatile acetate ester biosynthesis. Since esters are central contributors to melon aroma, refrigeration may weaken the sensory character of the product even when visual quality remains acceptable [16].

Fresh-cut fruits require particular attention under refrigerated storage because cutting intensifies respiration, exposes cellular contents, increases wound-related metabolism and creates a larger surface for volatile release and microbial colonization. In fresh-cut cantaloupe melon, storage at 4 °C was associated with a considerable decrease in several aroma compounds, especially esters, and this reduction was interpreted as an early step in freshness loss [79]. This example is important because it shows that refrigeration can slow microbial spoilage while still allowing aroma deterioration. In packaged fresh-cut fruits, the problem may be intensified when high humidity and condensation develop inside the package, because these conditions can favor microbial growth and the accumulation of off-odor metabolites if temperature control is not stable [75,76,79].

Berries also illustrate the difficulty of preserving aroma under cold storage. Strawberries are usually refrigerated to slow softening, decay and water loss, but their aroma is highly sensitive to storage duration, tissue integrity and preharvest conditions. In halved strawberry fruits, storage modified volatile organic compound profiles and affected gene expression related to quality maintenance, while the response was influenced by preharvest factors [80]. For packaged strawberries and other berries, this means that refrigerated storage should not be evaluated only by decay incidence or firmness retention. Changes in esters, furanones, terpenoids, aldehydes, and alcohols may alter the characteristic fruity and floral profile even before the product becomes visually unacceptable [12,14,75,80].

Refrigeration also modifies aroma perception through physical effects on volatile release. At low temperature, the vapor pressure of aroma compounds decreases, reducing their transfer from fruit tissue to the package headspace and from the food matrix to the nasal cavity during consumption. This means that refrigerated fruit may smell less intense immediately after removal from storage, even if part of the volatile pool remains present in the tissue [14,48,74]. In practical terms, this creates a distinction between chemical preservation and sensory availability. Some fruits may recover part of their aroma perception after tempering at room temperature before consumption, but this recovery depends on whether the volatile biosynthetic capacity has merely been slowed or has been irreversibly impaired by chilling stress, senescence or prolonged storage [14,15,16,77].

The possibility of flavor restoration after refrigeration depends on the mechanism responsible for aroma loss. When aroma weakening is mainly physical, caused by reduced vapor pressure, reduced volatile release and lower transfer from fruit tissue to the headspace or nasal cavity, part of the perceived aroma may recover after tempering at room temperature before consumption. When refrigeration has slowed, but not permanently impaired, ripening-related metabolism, partial recovery may also occur if the fruit remains physiologically capable of producing esters, lactones, terpenes, or other characteristic volatiles. However, aroma loss becomes largely irreversible when low temperature causes chilling injury, membrane dysfunction, oxidative stress, substrate depletion, prolonged senescence, microbial spoilage, or tissue breakdown. In these cases, warming the fruit may increase volatile release, but it cannot fully regenerate a native volatilome whose biosynthetic capacity has been damaged. Therefore, refrigerated active packaging should distinguish between reversible sensory unavailability, partially reversible metabolic suppression and irreversible physiological aroma loss [14,15,16,74,77,78,79,81]. The main aroma-loss scenarios and their degree of reversibility are summarized in Table 3.

In active packaging design, refrigerated storage should therefore be treated as a commodity-specific condition rather than a universal preservation solution. The package must maintain an atmosphere that slows respiration and microbial growth without imposing excessive O_2_ limitation or CO_2_ accumulation, while also controlling water vapor to avoid condensation. This balance is especially delicate under temperature fluctuations because small increases in temperature can sharply increase respiration, whereas the gas transmission properties of the package may not adjust at the same rate [75,76]. For aroma preservation, refrigerated active packaging should be validated through headspace gas composition, sensory descriptors and volatile profiling, including both the loss of desirable compounds and the accumulation of early off-flavor markers. This approach is necessary to avoid the misleading conclusion that refrigeration and packaging are successful simply because fruit remains firm, bright, and free of visible decay [13,14,76]. 

#### 3.1.2. Storage at Room Temperature

Storage at room temperature, including retail display, temporary in-store exposure, and domestic handling, represents a markedly different deterioration scenario from refrigerated storage. Moderate warming can increase the immediate perception of aroma because volatile compounds show higher vapor pressure and are released more readily from the fruit matrix into the package headspace and surrounding air. In climacteric fruits, room-temperature exposure may also allow ripening-related metabolism to continue, which can be desirable when fruit has been harvested at a mature but not fully ripe stage [48,51,52,74]. However, this apparent sensory advantage is usually short-lived. Higher temperature accelerates respiration, ethylene production, water loss, softening, membrane permeability, and substrate consumption, thereby shortening the period during which sweetness, acidity, texture, and aroma remain balanced [74,88]. For packaged fruits, the main risk is that the same warming that enhances aroma release may also accelerate the biochemical and microbiological routes that lead to off-flavor formation.

Room-temperature storage affects taste primarily through the faster use of sugars and organic acids as respiratory substrates. As respiration increases, soluble sugars and organic acids may decline or become less balanced, reducing sweetness, freshness, and the characteristic sugar/acid ratio of the fruit [48,68,74]. In whole fruits, these changes may be gradual, but in fresh-cut products they can occur more rapidly because cutting increases surface area, disrupts tissue compartmentalization, and stimulates wound-related metabolism. Fresh-cut fruits have been shown to lose quality more rapidly than whole fruits during storage, with changes depending on the commodity, processing intensity and storage conditions [89]. Therefore, in room-temperature conditions, the deterioration of taste should not be understood only as a change in soluble solids or acidity, but as a combined loss of metabolic balance, texture integrity, and aroma support.

The effect of room temperature on aroma is even more complex because it can initially increase volatile emission while simultaneously destabilizing the native volatilome. In the early stages of exposure, warmer conditions may enhance the perception of fruity, floral, or ripe notes, particularly if esters and terpenoids are still being produced or released. As storage progresses, however, desirable volatiles may decline because of substrate depletion, enzyme imbalance, volatilization, oxidation, or microbial transformation [14,53,54,74]. Fresh-cut pineapple provides a useful example: when stored at 20 °C, free volatiles may increase initially but later decline sharply, while odor-associated compounds such as ethyl acetate and ethanol become more prominent at late storage stages [90]. This illustrates a key point for the present review: stronger aroma at room temperature does not necessarily mean better aroma quality; it may indicate a transition from varietal aroma towards overripe or fermentative notes.

Room-temperature storage is particularly problematic in packaged fruits because fruit respiration and package gas transfer may become mismatched. Packaging films are often selected for a target commodity, product load, and storage temperature. If fruit is held at room temperature after being packed for refrigerated distribution, respiration may increase faster than gas transmission through the film. O_2_ may then fall below the fruit’s tolerance threshold, while CO_2_ accumulates in the headspace [11,75,76]. Under these conditions, fruit tissues may shift towards fermentative metabolism, producing ethanol, acetaldehyde, ethyl acetate, and related compounds associated with alcoholic, solvent-like, overripe or fermented notes [14,17,76]. The risk is especially high in fresh-cut fruits because damaged tissues respire more rapidly and because leaked cellular fluids provide substrates for microbial growth [75,88,89].

Fresh-cut melons are a good example of this temperature sensitivity. Fresh-cut cantaloupe is valued for its aroma but is highly perishable, and storage temperatures higher than the ideal refrigerated range can rapidly alter quality markers. A multi-trait study comparing 0, 5, and 10 °C found that temperature influenced microbial load, vitamin C loss, phenolic content, and the volatile organic compound profile; the authors also identified volatile markers associated with storage time and temperature [91]. Although 10 °C is still below typical room temperature, this evidence is useful because it shows that even moderate deviations from optimal refrigeration can reshape the volatilome of fresh-cut fruit. At true room temperature, the same direction of change would be expected to occur faster and with greater risk of microbial and fermentative off-odor development [74,75,91].

Microbial deterioration becomes a central issue at room temperature. Yeasts, molds, and bacteria grow more rapidly when temperature increases, especially when package humidity is high and fruit surfaces are wet or damaged. In fresh-cut fruits, cellular leakage provides sugars, organic acids, and amino acids that microorganisms can metabolize into alcohols, organic acids, esters, ketones, sulfur compounds, and other microbial volatile organic compounds [75,88,89]. These compounds may mask the native fruit aroma and generate fermented, sour, musty, earthy, vinegar-like or sulfury, notes. The problem is intensified when condensation forms inside the package, because surface moisture facilitates microbial colonization and can create localized zones of low oxygen. Thus, in room-temperature storage, physiological fermentation, and microbial spoilage may reinforce each other rather than acting as separate deterioration routes [14,17,75].

From the perspective of active packaging, room-temperature exposure should be treated as a realistic stress condition, not as an exceptional event. In commercial chains, fruit may experience temporary warming during loading, retail display, consumer transport, or domestic storage. Active systems such as antimicrobial films, ethylene scavengers, moisture absorbers, oxygen scavengers, and CO_2_ regulators may respond differently under these conditions because their kinetics are affected by temperature and relative humidity [18,19,75,76]. A package that performs well under stable refrigeration may therefore fail at room temperature if it cannot compensate for accelerated respiration, condensation, microbial growth, or volatile accumulation. For this reason, studies on active packaging for fruit aroma preservation should include, where possible, a temperature-abuse or room-temperature scenario, together with headspace gas monitoring, sensory analysis, and volatilome profiling. This would make it possible to determine whether the system preserves the original sensory identity of the fruit or merely delays visible deterioration while allowing aroma imbalance to develop [13,14,76,90,91].

### 3.2. Formation of Off-Odors and Off-Flavors

Off-odors and off-flavors represent one of the clearest forms of sensory failure in packaged fresh fruits because they indicate that the native aroma profile has moved away from the expected sensory identity of the product. This deterioration may occur through two complementary processes: the loss or suppression of desirable odor-active volatiles, and the accumulation of compounds perceived as alcoholic, solvent-like, vinegar-like, musty, stale, grassy, sulfury, rancid, or overripe [14,17,92]. The distinction is important because a fruit can lose aroma quality even without producing an obvious unpleasant odor. For example, a reduction in esters, terpenoids, lactones, or norisoprenoids may make the fruit seem flat or weakly aromatic, whereas the accumulation of ethanol, acetaldehyde, ethyl acetate, acetic acid, sulfur-containing compounds, or microbial volatiles may actively generate rejection [14,17,53,54,92].

The formation of off-odors is strongly concentration- and matrix-dependent. Some volatile compounds that are desirable at low or moderate levels can become defective when they exceed their sensory balance within the fruit matrix. Esters are a clear example: they contribute fruity and sweet notes in apple, melon, banana, strawberry and pear, but excessive accumulation of ethyl acetate may produce solvent-like or fermented impressions [17,53,57,58,59,60,61]. Aldehydes show a similar duality. C6 aldehydes can provide freshness and green notes, but high concentrations after cutting, bruising, or lipid oxidation may make the aroma harsh, grassy or damaged [53,55,56]. Sulfur-containing volatiles may contribute varietal complexity in some fruits, yet under anaerobic or microbial conditions they can rapidly shift towards sulfury, cabbage-like, or onion-like descriptors [14,17,62]. Therefore, off-flavor formation should be interpreted as a disruption of volatile balance rather than as the simple presence or absence of a single compound.

In packaged fruits, the most frequent off-flavor route is associated with low oxygen availability and the activation of fermentative metabolism. When O_2_ concentration falls below the fruit’s lower oxygen tolerance, pyruvate may be redirected towards acetaldehyde through pyruvate decarboxylase and then towards ethanol through alcohol dehydrogenase. Acetaldehyde and ethanol are natural metabolites involved in fruit ripening and aroma formation, but they accumulate much more intensely under partially or fully anaerobic conditions, including controlled or modified atmospheres, wax coatings, or poorly ventilated packaging systems [17,92]. Their accumulation may be accompanied by ethyl acetate and other esters that intensify alcoholic, fermented, or solvent-like notes. This route is particularly relevant for active packaging because systems that reduce O_2_ too strongly, or that are not matched to the product respiration rate, may preserve visual appearance while damaging flavor [11,75,76,92].

Off-flavors may also arise from oxidative and wound-related metabolism. Mechanical injury, cutting, bruising, chilling damage, and senescence disrupt cell compartmentalization, allowing enzymes and substrates to interact more freely. Lipoxygenase-related reactions may increase the formation of aldehydes and alcohols derived from membrane fatty acids, while oxidative stress can alter membrane integrity and promote the appearance of stale, grassy, or oxidized notes [53,55,56,77,78]. At the same time, changes in alcohol dehydrogenase and alcohol acyltransferase activity may alter the balance between aldehydes, alcohols, and esters, making the profile less characteristic of the ripe fruit [56,57,58,59,60]. In fresh-cut fruits, this route is especially important because the exposed surface promotes volatile release, tissue leakage, respiration, and microbial access to nutrients [71,88,89,93].

Microbial activity adds another layer of complexity because microorganisms can both produce their own volatile organic compounds and modify fruit-derived compounds. Yeasts, molds and bacteria may generate alcohols, organic acids, ketones, esters, sulfur compounds, and other microbial volatiles associated with fermented, musty, earthy, sour, vinegar-like, or spoiled notes [75,82,93]. These microbial aromas do not simply add to the native fruit aroma; they can mask varietal notes, accelerate tissue degradation, and contribute to package-atmosphere imbalance by consuming O_2_ and producing CO_2_. The problem is intensified when high relative humidity, condensation, damaged tissues, or leaked juices create localized niches for microbial growth inside the package [75,82,88,89,93].

Analytically, off-odor formation cannot be properly assessed only by measuring total volatile abundance. Some fruit with a high total volatile concentration may still be defective if the increase is dominated by fermentation products or microbial metabolites. Conversely, a fruit with a lower total volatile concentration may be acceptable if the main odor-active compounds remain balanced and off-flavor markers remain below sensory thresholds [14,17,23]. For this reason, volatilome-based evaluation should combine untargeted profiling with targeted monitoring of key markers such as ethanol, acetaldehyde, ethyl acetate, acetic acid, C6 aldehydes, sulfur-like compounds, and microbial VOCs. Gas chromatography-olfactometry and odor activity value approaches are useful because they help distinguish compounds that are abundant from those that actually contribute to aroma perception [23].

For active packaging, the prevention of off-odors and off-flavors requires a balance between slowing deterioration and avoiding excessive stress. Antimicrobial systems may reduce microbial spoilage, ethylene scavengers may delay ripening and senescence, moisture absorbers may reduce condensation, and gas-modulating systems may help maintain adequate O_2_ and CO_2_ levels. However, these functions must be carefully adjusted to the fruit species, cultivar, maturity stage, cut status, product load, film permeability, and expected storage temperature [18,19,75,76]. An active system that is too aggressive in oxygen removal, too weak in moisture control, or poorly adapted to temperature fluctuations may shift the package microenvironment towards fermentation or microbial off-odor formation. Therefore, off-flavor prevention should be considered a central performance criterion in active packaging design, not a secondary consequence of shelf-life extension [14,17,76,92].

#### 3.2.1. By Microbial Deterioration

Microbial deterioration is one of the most important causes of off-odor and off-flavor development in packaged fresh fruits, particularly in berries, soft fruits, and fresh-cut products. Fruits are naturally exposed to microorganisms in the field, during harvest, washing, cutting, packing, transport, retail handling, and domestic storage. Although not all microorganisms immediately cause spoilage, the combination of high-water activity, available sugars and organic acids, fragile tissues, and postharvest handling damage creates favorable conditions for yeasts, molds, and bacteria to develop during shelf life [75,82,83,84,93]. In packaged fruits, microbial activity is especially relevant because the package can retain humidity, concentrate volatile metabolites, and modify O_2_/CO_2_ levels, thereby influencing both microbial growth and the sensory expression of spoilage [75,76,83].

The microbial ecology of fresh fruits is strongly affected by commodity type, pH, surface structure, maturity stage, tissue integrity, and storage temperature. Berries and grapes, for example, are particularly vulnerable to molds and yeasts because of their soft tissues, high sugar content and delicate epidermis. A study on fresh berries, grapes and citrus fruits showed that mold and yeast contamination varied among fruit types, supporting the idea that microbial deterioration cannot be generalized across commodities [84]. In fresh-cut fruits, the risk is even higher because peeling, slicing or dicing removes natural protective barriers, releases cellular nutrients and creates large, wounded surfaces. These conditions accelerate both physiological deterioration and microbial colonization, making microbial spoilage a central limitation for sensory shelf life [83,88,89,93].

Microbial off-odors arise because microorganisms metabolize fruit-derived substrates into volatile compounds that can interfere with the native fruit aroma. Yeasts may convert sugars into ethanol, higher alcohols, esters, organic acids, and carbonyl compounds, while molds and bacteria can generate a broader set of alcohols, aldehydes, ketones, acids, sulfur compounds, terpenoid-like compounds, and musty or earthy volatiles [82,85,86]. In a strawberry-based model system, yeasts isolated from strawberries produced volatile compounds such as acetone, ethyl acetate, ethanol, isopropyl acetate, ethyl butyrate, 1-propanol, 2-methyl-1-propanol, 1-butanol, 2-methyl-1-butanol, 3-methyl-1-butanol, 1-hexanol, and hexyl acetate, while sugar concentrations decreased during storage [85]. This is particularly important for volatilome interpretation because some of these compounds also occur naturally in fruits. Their presence alone does not necessarily indicate spoilage; their concentration, timing, microbial context, and balance with varietal aroma compounds determine their sensory meaning [14,17,85].

This overlap between fruit-derived and microbially derived volatiles makes microbial deterioration difficult to diagnose using only untargeted volatile abundance. Ethanol and ethyl acetate, for instance, may be produced by fruit tissues under low-oxygen conditions, but they may also arise from yeast metabolism in damaged or sugar-rich tissues [17,85,92]. Similarly, esters can contribute desirable fruity notes when they are part of the normal ripening profile, but they may produce fermented, solvent-like or overripe impressions when microbial activity or anaerobic metabolism shifts their concentration beyond the expected sensory range [17,53,57,58,59,60,61,85]. Therefore, microbial deterioration should be assessed by combining volatile markers with microbial counts, sensory descriptors, tissue condition and headspace gas composition, rather than by interpreting individual compounds in isolation [23,75,83,85].

Molds play a particularly important role in packaged fruit deterioration because they can grow on damaged surfaces, latent infections, or zones of high humidity. Common postharvest fungal problems in fruits include infections caused by genera such as Botrytis, Penicillium, and Rhizopus, depending on the commodity and storage conditions. In strawberries, fungal infection has been shown to modify the volatile profile, and an electronic nose combined with GC-MS was able to discriminate early fungal disease development and classify different fungal infection types before deterioration became fully advanced [87]. This supports the relevance of volatile monitoring as an early-warning approach for microbial spoilage. In the context of active packaging, this point is especially valuable because microbial volatile organic compounds may reveal the beginning of sensory failure before visible decay is severe enough to be detected by conventional quality inspection [23,82,87].

The packaging atmosphere can either restrict or promote microbial off-flavor formation depending on how it interacts with temperature, product load, film permeability, and moisture. Modified atmospheres with reduced O_2_ and elevated CO_2_ may slow the growth of some aerobic spoilage organisms and reduce respiration, but they do not sterilize the product. If temperature control is poor, or if the atmosphere becomes too restrictive, facultative anaerobic microorganisms and yeasts may continue to grow and produce fermentative metabolites [75,76,83,85]. High CO_2_ may also alter the competitive balance among microbial groups, while condensation inside the package can create wet microzones that favor microbial colonization. Thus, microbial deterioration in packaged fruits is not only a function of microbial contamination at packing; it is also a consequence of the microenvironment created during shelf life [75,76,83,86].

Microbial deterioration also affects taste, not only aroma. As microorganisms consume sugars and organic acids, the fruit may lose sweetness, freshness, and the characteristic sugar/acid balance. At the same time, microbial production of organic acids, ethanol, and other metabolites may introduce sour, fermented, or vinegar-like notes. Tissue maceration caused by microbial enzymes can increase softening, leakage, and loss of juiciness, while fungal growth or bacterial spoilage may generate bitterness, stale mouthfeel, or an unpleasant aftertaste [83,86,88,89,93]. These effects are especially problematic in fresh-cut fruits because texture collapse and liquid exudation increase the availability of nutrients, reinforcing microbial growth and creating a feedback loop between tissue breakdown, package humidity, and off-flavor formation [83,88,89,93].

For active packaging, microbial deterioration should be considered a sensory problem as much as a safety or decay problem. Antimicrobial films, coatings, pads, or emitters may reduce microbial growth, but their effectiveness should be evaluated together with aroma preservation. Some antimicrobial compounds, including essential oils or plant-derived volatiles, may have their own strong odor and could mask or distort the native fruit aroma if the release rate is not properly controlled [18,19,82]. Likewise, moisture absorbers may reduce condensation and microbial growth, but excessive dehydration can damage texture and freshness perception. The most suitable active packaging system is therefore not necessarily the one with the strongest antimicrobial effect, but the one that limits microbial growth while maintaining the balance of fruit-derived odor-active compounds, suppressing microbial off-odor markers and preserving the expected taste profile [14,18,19,75].

This interpretation should be balanced by recognizing that microbial volatile production is not inherently undesirable in all food systems. In controlled fermentations, yeasts, lactic acid bacteria, and other microorganisms may generate esters, alcohols, organic acids, carbonyl compounds, and sulfur-containing volatiles that contribute pleasant fruity, floral, dairy-like, alcoholic, or fermented notes. However, packaged fresh fruits are not normally intended to develop a fermented sensory profile. Their commercial and sensory value depends on preserving the native fruit volatilome generated during ripening. Therefore, the same microbial metabolites that may be desirable in fermented foods become negative in fresh fruits when they appear outside the expected varietal context, accumulate above their sensory compatibility range, are associated with tissue leakage or microbial growth, or shift the aroma from fresh, fruity, and varietal notes towards fermented, sour, musty, sulfurous, stale, or rotten descriptors. For this reason, microbial volatiles in packaged fresh fruits should be interpreted through the combined evidence of compound identity, concentration, timing, microbial counts, tissue condition, headspace gas composition, and sensory descriptors [82,83,84,85,86,92,93].

From a methodological perspective, microbial deterioration should be incorporated into volatilome-oriented packaging studies through integrated monitoring. At a minimum, studies should combine microbial counts, headspace O_2_/CO_2_, temperature history, visible decay, sensory descriptors, and targeted volatile markers such as ethanol, acetaldehyde, ethyl acetate, acetic acid, higher alcohols, sulfur-like compounds, and musty or earthy volatiles [14,17,23,85,86,87]. For more detailed studies, untargeted GC-MS, GC-olfactometry, electronic nose systems and multivariate analysis can help distinguish physiological aroma changes from microbial spoilage signatures. This distinction is essential for the development of active packaging systems that preserve the fruit volatilome rather than simply delaying visible mold growth [23,82,87].

#### 3.2.2. By Enzymatic Deterioration

Enzymatic deterioration is a central mechanism of taste and aroma loss during the shelf life of packaged fresh fruits, especially when tissue integrity has been weakened by ripening, cutting, bruising, chilling stress, senescence, or microbial colonization. In intact fruit, many enzymes and substrates are spatially separated by membranes, organelles, and cell wall structures. Once this compartmentalization is disrupted, enzymes gain access to substrates that were previously less available, accelerating browning, softening, lipid oxidation, volatile transformation, and the release or depletion of taste-active compounds [71,73,88,89,93]. This process is particularly relevant for fresh-cut fruits because minimal processing creates exposed surfaces, stimulates wound responses, increases respiration and ethylene production, and favors faster contact between enzymes, oxygen, and cellular substrates [73,88,89,93].

One of the main enzymatic routes affecting aroma is the oxidation of membrane lipids through lipoxygenase-related pathways. Linoleic and linolenic acids can be converted into aldehydes and alcohols such as hexanal, (E)-2-hexenal, (Z)-3-hexenal, hexanol, and (Z)-3-hexenol, which are associated with green, grassy, and fresh-cut notes [53,55,56]. At moderate levels, these compounds may reinforce freshness, but after cutting, bruising, or storage stress they can become excessive and contribute to harsh, damaged or unripe sensory impressions. The same pathway also interacts with alcohol dehydrogenase and alcohol acyltransferase activity, which regulate the balance among aldehydes, alcohols, and esters [56,57,58,59,60]. Therefore, enzymatic deterioration does not necessarily mean the simple loss of volatiles; in many cases, it means a redistribution of the volatilome towards a less balanced profile, with lower varietal identity and a higher proportion of wound-related or oxidized notes.

Enzymatic browning is another important route of sensory deterioration because it affects appearance, taste, and, indirectly, aroma perception. Polyphenol oxidase and peroxidase catalyze the oxidation of phenolic compounds into quinones, which can polymerize into brown pigments and modify the redox environment of the tissue [94]. Although browning is usually evaluated visually, its sensory consequences are broader. Oxidation of phenolics may change bitterness, astringency, and mouthfeel, while tissue browning can reduce consumer expectation of freshness before the fruit is even consumed [71,94]. In fruits such as apple, pear, peach, banana, and some fresh-cut tropical fruits, browning reactions are particularly relevant because cutting or bruising exposes phenolic substrates to oxygen and oxidative enzymes. Packaging atmospheres with reduced O_2_ may slow browning, but excessive O_2_ limitation can simultaneously increase fermentative metabolism; therefore, anti-browning control must be balanced with the need to preserve normal aroma metabolism [75,76,92,94].

Cell wall-degrading enzymes also contribute to the deterioration of taste and aroma because texture strongly determines how flavor is perceived during consumption. Polygalacturonases, pectin methylesterases, beta-galactosidases, cellulases, expansins, and other cell wall-modifying proteins participate in softening during ripening, but excessive or uncontrolled activity during storage leads to tissue collapse, loss of crispness, leakage, mealiness, or reduced juiciness [71,95,96]. These physical changes affect flavor in several ways. First, they modify the release of sugars, acids, and volatiles during mastication. Second, they increase cellular leakage and surface wetness, creating conditions that favor microbial growth. Third, they can expose additional substrates for oxidative and fermentative reactions. Thus, texture deterioration should not be separated from aroma deterioration; both processes are connected through tissue structure, enzyme activity, and volatile release dynamics [71,88,89,93,95].

Enzymes involved in ripening-related aroma biosynthesis may also become unbalanced during storage. Alcohol dehydrogenase and alcohol acyltransferase are essential for the formation of alcohols and esters that contribute to desirable fruit aroma, but their activity depends on oxygen availability, substrate supply, maturity stage, ethylene regulation, and temperature [56,57,58,59,60]. Under suitable ripening conditions, these enzymes help form fruity esters in apple, banana, strawberry, melon, and pear. Under stress conditions, however, altered enzyme activity can contribute to the accumulation of ethanol, acetaldehyde, ethyl acetate, or atypical ester profiles, particularly when packaging promotes low-O_2_ conditions or when temperature abuse accelerates respiration [17,57,58,59,60,76,92]. This point is important because the same enzymatic families can support desirable aroma formation during ripening and off-flavor development during shelf life, depending on the physiological context.

Glycosidase-related activity also deserves attention in packaged fruits because many aroma compounds occur as glycosidically bound precursors. Hydrolysis of these bound forms can release terpenes, norisoprenoids, alcohols, phenols, and other aglycones that may enhance varietal aroma [8,66]. However, uncontrolled hydrolysis during storage, especially when tissue damage or microbial enzymes are involved, may change the timing and balance of aroma release. A limited release of bound volatiles may improve aroma complexity, whereas excessive or poorly synchronized release may contribute to atypical notes or accelerate the depletion of latent aroma reserves [8,66,82]. This mechanism is still less studied in active packaging than respiration, microbial growth, or firmness retention, but it is highly relevant for a volatilome-oriented interpretation of fruit sensory quality.

The interaction between enzymatic deterioration and packaging atmosphere is particularly delicate. Reduced O_2_ levels may slow oxidative browning and some lipid oxidation reactions, but if O_2_ becomes too low, the fruit may shift towards anaerobic metabolism, leading to the accumulation of fermentation-related compounds [75,76,92]. Elevated CO_2_ may delay some physiological processes and microbial growth, but excessive CO_2_ can contribute to physiological disorders, altered acidity, and abnormal flavor in sensitive commodities [11,75,76]. Similarly, high humidity inside the package reduces water loss but can increase condensation, tissue maceration, and microbial enzyme activity. Therefore, enzymatic deterioration cannot be managed by a single packaging target such as low oxygen or high humidity; it requires a carefully balanced microenvironment adapted to the fruit species, cut status, maturity stage, and temperature conditions [73,75,76].

For active packaging, controlling enzymatic deterioration means preserving metabolic balance rather than fully suppressing metabolism. Antioxidant systems, anti-browning agents, edible coatings, moisture regulators, and atmosphere-modifying materials may reduce enzymatic damage, but their effectiveness should be evaluated in relation to taste and aroma preservation, not only visual quality [18,19,73]. A coating or film that reduces browning but suppresses ester formation may improve appearance while weakening aroma. Conversely, a package that maintains volatile formation but fails to control phenolic oxidation or softening may preserve aroma but lose consumer acceptance through poor appearance and texture. Future studies should therefore combine enzyme-related quality markers with volatile profiling, sensory analysis, sugar/acid balance, firmness, browning index, and headspace gas monitoring to determine whether active packaging truly protects the sensory identity of fresh fruit [14,23,71,73,76].

#### 3.2.3. By Mechanical Damage

Mechanical damage is one of the earliest and most underestimated triggers of taste and aroma deterioration in fresh fruits. It can occur during harvesting, field handling, washing, grading, sorting, packing, transport, retail display, and consumer handling, and may result from impact, compression, vibration, abrasion, puncture, cutting, or friction [97,98,99,100]. Although bruising is often treated as a visual defect, its consequences are physiological and biochemical. Mechanical stress disrupts cell walls and membranes, breaks tissue compartmentalization, increases electrolyte leakage, stimulates respiration and ethylene production, accelerates water loss, and exposes internal substrates to oxygen and enzymes [97,98,99]. These changes directly affect fruit volatilome because the loss of tissue integrity modifies both volatile biosynthesis and volatile release.

The sensory impact of mechanical damage depends on the severity, location, and visibility of the injury. Some defects, such as cuts, cracks, or crushed tissue, are immediately evident, whereas internal bruising or microstructural damage may remain hidden during early shelf life [97,98]. This is particularly problematic for packaged fruits because visually acceptable products may already contain damaged tissues that are metabolically unstable. Once enclosed in a package, injured fruit can modify the headspace faster than sound fruit because respiration and transpiration are increased, while damaged cells release sugars, organic acids, phenolics, amino acids, and lipid substrates. As a result, the package may shift more rapidly towards high humidity, O_2_ depletion, CO_2_ accumulation, and volatile imbalance, especially when the product load is high or the film permeability was designed for undamaged fruit [75,76,97,100].

Mechanical injury has an immediate effect on aroma through wound-induced volatile production. Damage to membranes increases the availability of fatty acid substrates for lipoxygenase-related reactions, promoting the formation of C6 aldehydes and alcohols such as hexanal, (E)-2-hexenal, (Z)-3-hexenal, hexanol, and (Z)-3-hexenol [53,55,56]. These compounds can be desirable when they provide moderate fresh and green notes, but after bruising, cutting, or crushing they may become dominant and give the fruit an excessively grassy, harsh, raw, or damaged aroma. In strawberry fruit, wounding has been shown to stimulate the biosynthesis of trans-2-hexenal, confirming the close connection between tissue injury, lipoxygenase activity, and the rapid formation of green-note volatiles [101]. Therefore, wound-related volatiles should be interpreted carefully: they may indicate freshness in freshly cut tissue, but also early deterioration when their concentration exceeds the normal sensory balance.

The response to mechanical damage is also maturity dependent. As fruit ripens, tissue firmness declines, cell wall structure changes, and susceptibility to compression or impact damage often increases. At the same time, the volatile pathways active at each maturity stage differ, so the effect of wounding on aroma is not constant throughout ripening. In tomato, wounding elicited ripening-stage-specific changes in gene expression and volatile production, showing that the same mechanical stimulus can generate different aroma outcomes depending on fruit developmental stage [102]. This has direct implications for packaged fruits: a ripe fruit may be more aromatic but also more fragile, whereas a less ripe fruit may tolerate transport better but possess a weaker volatilome. Packaging must therefore protect the fruit physically without forcing harvest at an excessively immature stage that compromises taste and aroma potential [48,52,97,102].

Mechanical damage also affects taste through changes in texture, juice release, and the exposure of taste-active compounds to enzymatic and microbial reactions. Bruised or compressed tissues may lose crispness, firmness, and juiciness, while cell rupture can lead to localized leakage of sugars, organic acids, and phenolics. These changes may initially increase the release of soluble compounds during consumption, but they usually accelerate loss of freshness, tissue collapse, and unbalanced mouthfeel during storage [71,73,88,95]. Phenolic oxidation after injury can increase browning and alter bitterness or astringency, while cell wall degradation may produce mealiness or a watery texture that weakens the perception of sweetness and freshness [73,94,95,96]. In this sense, mechanical damage does not only generate off-odor risk; it alters the physical basis through which taste and aroma are perceived.

A key consequence of mechanical damage is its interaction with microbial deterioration. Injured surfaces and leaked juices provide nutrients and water that facilitate the growth of yeasts, molds, and bacteria. This is especially relevant in fresh-cut fruits, berries, ripe stone fruits, and soft tropical fruits, where even small injuries can create localized spoilage niches inside the package [75,83,84,85,86,87,88,89,93]. Microorganisms developing on damaged tissues may produce ethanol, higher alcohols, esters, acids, ketones, sulfur compounds, and musty or earthy volatiles, which can mask the native aroma and generate fermented, sour or spoiled notes [82,85,86,87]. Thus, mechanical injury often acts as an initiating event that links enzymatic deterioration, microbial spoilage, and package-atmosphere imbalance.

Mechanical damage during transport deserves special attention because vibration and repeated low-intensity impacts can produce cumulative injury even when individual events appear minor. Transport-related vibration depends on road conditions, vehicle speed, vibration duration, package position, stack height, cushioning, package geometry, and fruit-to-fruit contact [100]. This type of damage is relevant for aroma preservation because cumulative bruising may not be detected immediately, yet it can accelerate respiration, softening, water loss, and microbial susceptibility during subsequent storage. For active packaging, this means that chemical or biological activity alone is insufficient if the package does not provide adequate structural protection. Antimicrobial films, ethylene scavengers or moisture absorbers cannot fully compensate for bruising caused by poor cushioning, excessive compression, overfilling, or unstable transport conditions [97,98,99,100].

The relationship between mechanical damage and active packaging should therefore be interpreted through a combined physical-biochemical framework. Cushioning pads, absorbent pads, molded trays, compartmentalized punnets, dividers, corrugated liners, air-cell structures, and shock- or vibration-absorbing package designs are not active packaging in the strict sense when they do not release, absorb or scavenge active compounds. However, they are critical packaging-design elements because they reduce the primary mechanical injury that triggers secondary sensory deterioration. By limiting bruising, compression, cell rupture and fruit-to-fruit impact, these structures can reduce wound-related C6 aldehydes and alcohols, juice leakage, localized condensation, microbial niches, respiration increases, and fermentative off-flavor formation. Conversely, an antimicrobial, ethylene-scavenging or gas-regulating active package may still fail to preserve aroma if poor mechanical protection allows tissue damage during transport. For this reason, flavor-oriented active-packaging studies should report cushioning strategy, tray or punnet geometry, pad type, stack height, fill ratio, vibration or impact simulation, and visible or microscopic tissue damage together with volatile and sensory outcomes [97,98,99,100,101,102].

The design of packaging systems should therefore integrate mechanical protection with atmosphere and moisture control. Trays, punnets, pads, cushioning materials, headspace volume, fill weight, sealing conditions, and stacking resistance influence the degree of tissue injury and, indirectly, the evolution of the volatilome [97,98,99,100]. A package that minimizes impact and compression can help preserve the native aroma profile by reducing cell rupture, lipid oxidation, juice leakage and microbial colonization. Conversely, a package with good gas-modifying properties but poor physical protection may maintain an apparently favorable O_2_/CO_2_ atmosphere while allowing local bruising and off-flavor development. For fresh-cut fruits, mechanical damage is partly inherent to processing, but its consequences can still be reduced through sharp cutting tools, gentle handling, adequate washing and drying, suitable portion size, and packaging that prevents excessive tissue movement and free-liquid accumulation [73,88,89,93].

From a methodological perspective, mechanical damage should be incorporated into volatilome-oriented studies on active packaging rather than treated only as a handling artifact. Studies should report the physical condition of the fruit before packaging, the presence of bruising or cut surfaces, package fill ratio, headspace volume, transport simulation or vibration conditions, and the degree of juice leakage or condensation during storage [97,98,99,100]. Volatile profiling should include both desirable aroma compounds and wound-related markers such as C6 aldehydes and alcohols, together with fermentative and microbial markers when damaged tissues are present [14,17,53,55,56,85,86,87,101]. This would allow researchers to distinguish whether off-flavor formation is mainly caused by the packaging atmosphere itself, by pre-existing mechanical injury, or by the interaction between damaged tissues and the package microenvironment.

#### 3.2.4. By Packaging System

The packaging system can protect fresh fruits against deterioration, but it can also become a direct cause of taste and aroma loss when its properties are not properly matched to the physiology of the product. In packaged fruits, the final microenvironment depends on the interaction between fruit respiration, product mass, headspace volume, film permeability, perforation level, humidity, temperature, and storage duration [11,75,76]. When these variables are balanced, packaging can reduce water loss, slow respiration, limit microbial growth, and preserve freshness. When they are poorly adjusted, the same package can promote O_2_ depletion, excessive CO_2_ accumulation, condensation, volatile imbalance, fermentative metabolism, and off-flavor formation [14,17,75,76]. Therefore, the packaging system should not be considered a neutral container, but an active determinant of the fruit volatilome during shelf life.

The most evident packaging-related route of off-flavor formation is gas imbalance. Modified atmosphere packaging is usually designed to reduce O_2_ and increase CO_2_ to levels that slow respiration and senescence without inducing physiological damage. However, if O_2_ falls below the tolerance threshold of the fruit, aerobic respiration becomes insufficient and fermentative metabolism is activated, leading to the accumulation of acetaldehyde, ethanol, ethyl acetate, and related compounds [17,76,92]. These volatiles may generate alcoholic, solvent-like, fermented, or overripe notes, especially in fruits with high respiration rates or in fresh-cut products with exposed tissues. The risk becomes greater when packages designed for refrigeration are exposed to room temperature, because fruit respiration increases faster than the package can compensate through gas transmission [74,75,76,90]. Under these conditions, visual shelf life may appear extended, while sensory shelf life is already compromised.

The effect of packaging on volatile composition is not limited to fermentation markers. Packaging can reshape the whole aroma profile by modifying the relative abundance of esters, aldehydes, alcohols, terpenes, sesquiterpenes, and other odor-active compounds. A study using a biopolymer-coated polyethylene active film showed that packaging affected the volatile composition of fruits through the combined influence of modified atmosphere and packaging-shaped bacterial communities [21]. In that work, the active film helped reduce off-flavor production and preserve sesquiterpenes in longan, partly through antimicrobial and moisture-absorbing effects, whereas in strawberry the high respiration rate made the volatile profile more dependent on the atmosphere created inside the package. In strawberries, hypoxic conditions increased ethyl esters associated with off-flavor while decreasing methyl and hexyl esters linked to typical fruity aroma [21]. This example is particularly relevant because it shows that the same active packaging concept may have different sensory consequences depending on the fruit’s respiration rate, microbial ecology and tolerance to low O_2_.

Another packaging-related mechanism is aroma scalping, which refers to the sorption of volatile aroma compounds by packaging materials. Many fruit aroma compounds are hydrophobic, low-molecular-weight molecules that can partition into polymeric materials, reducing their availability in the fruit headspace and altering the perceived aroma balance [27,28]. This is especially important for esters, terpenes, aldehydes, and other compounds with high sensory relevance at low concentrations. Flavor scalping may reduce global aroma intensity, but it may also produce a more subtle distortion: some compounds are absorbed more strongly than others, so the package does not simply reduce aroma but changes its proportions [27,28]. In fruits whose sensory identity depends on delicate volatile ratios, such as strawberry, melon, citrus, peach, or apple, selective sorption of key odorants may weaken varietal identity even when total volatile production remains relatively high [3,14,23,53].

The extent of aroma scalping depends on the chemical properties of both the volatile compounds and the packaging material. Non-polar compounds tend to interact more strongly with non-polar polymers, while polymer density, crystallinity, layer structure, additives, contact time, temperature, and food matrix composition influence sorption and diffusion [27,28]. Although many studies on scalping have used model systems or beverages, the mechanism is directly relevant to fresh fruits because packages contain an aqueous, humid headspace in which aroma compounds partition between tissue, package atmosphere, and polymer surface. In a sealed package, the film can therefore act as an unintended sink for odor-active volatiles. This issue deserves more attention in fruit active packaging studies, where the focus is often placed on gas composition, microbial counts and firmness, while polymer-volatile interactions remain insufficiently assessed [14,18,19,27,28].

Packaging may also alter aroma through the release of active compounds. Antimicrobial or antioxidant packaging systems based on essential oils, plant extracts, volatile compounds, or active labels can reduce microbial spoilage and oxidative deterioration, but they may also introduce their own aroma into the package headspace [18,19,22,103]. This effect can be beneficial if the active volatile is compatible with the fruit aroma and released at a low, controlled rate. However, it can be negative if the active compound masks the native volatilome, gives the fruit a spicy, medicinal, resinous, or artificial note, or remains at concentrations above consumer acceptance thresholds [103]. For example, active labels containing cinnamon essential oil extended the shelf life of Calanda peach and reduced infection while maintaining most positive sensory descriptors close to the initial quality level after storage, showing that active volatile release can be compatible with sensory preservation when the system is well designed [22]. Nevertheless, this result should not be generalized to all fruit–essential oil combinations, because the sensory impact depends on the fruit matrix, active compound, release kinetics, package volume, and storage temperature [22,103].

Moisture regulation is another packaging function with direct consequences for taste and aroma. High relative humidity reduces fruit dehydration and helps preserve firmness and juiciness, but excessive humidity and condensation promote microbial growth, surface wetness, tissue maceration, and off-odor formation [12,75,83]. Conversely, excessive moisture removal may limit condensation but can increase weight loss, shriveling, and textural dryness, reducing the perception of freshness and sweetness. Moisture-absorbing pads, hydrophilic layers and active films may therefore contribute to aroma preservation indirectly by reducing wet microenvironments that support microbial growth. However, their performance must be balanced against the need to maintain juiciness and avoid excessive dehydration [12,21,75,76]. This balance is particularly critical in berries and fresh-cut fruits, where small amounts of leaked juice can create localized spoilage niches inside the package.

The packaging system can also influence taste and aroma through ethylene management. In climacteric fruits, ethylene is involved in ripening, softening, pigment development, and volatile biosynthesis [51,52,57]. Ethylene scavengers or absorbers can delay ripening and senescence, but excessive suppression of ethylene-related processes may also limit the development of desirable aroma, especially in fruit harvested before full sensory maturity [48,51,52,57]. In this sense, ethylene control should not be interpreted only as a tool for slowing ripening. It must be adjusted according to the fruit’s maturity stage and intended market window. A package that delays softening but prevents the formation of ripe-fruit esters, lactones, or other characteristic volatiles may extend commercial life while reducing eating quality [48,52,57,60].

The interaction between packaging and temperature remains decisive for all these mechanisms. Film permeability, fruit respiration, active compound release, volatile sorption, and microbial growth are all temperature-dependent [21,74,75,76]. A package that produces a favorable atmosphere at 4–5 °C may become hypoxic at 10–20 °C; an antimicrobial active label with acceptable release under refrigeration may become too intense at room temperature; a moisture-absorbing system that works under stable cold storage may be overwhelmed by condensation during temperature fluctuations [21,22,75,76,103]. Therefore, packaging-related aroma deterioration should be studied under realistic postharvest scenarios, including cold-chain interruptions, retail display, and domestic handling. Otherwise, the sensory performance of the packaging system may be overestimated.

From a methodological point of view, the packaging system should be evaluated as part of the volatilome rather than as an external preservation factor. Studies should report package material, thickness, permeability, perforation, headspace volume, product-to-headspace ratio, active component concentration, release or absorption kinetics, O_2_/CO_2_ evolution, relative humidity, condensation, and temperature history [21,75,76]. Volatile profiling should include both fruit-derived aroma compounds and packaging-sensitive markers such as ethanol, acetaldehyde, ethyl acetate, key esters, terpenoids, aldehydes, sulfur-like volatiles, and any active volatile released by the packaging system [14,17,21,22,23,103]. This approach would make it possible to distinguish whether sensory deterioration originates from fruit physiology, microbial spoilage, material sorption, active compound release, or a mismatch between fruit respiration and package permeability.

The central implication is that packaging performance cannot be judged only by the extension of visual shelf life. A package may reduce decay and weight loss while still weakening aroma through scalping, suppressing ester biosynthesis, inducing hypoxia, or masking fruit volatiles with active compounds [14,17,21,22,27,28,103]. Conversely, a well-designed active package can help preserve the native fruit volatilome by maintaining adequate gas balance, limiting condensation, reducing microbial growth, and avoiding excessive interaction between aroma compounds and packaging materials [18,19,21,22]. For this reason, the relationship between active packaging and sensory quality must be evaluated through an integrated framework that combines conventional shelf-life parameters with volatilome analysis and sensory validation. This point provides the logical transition to the next part of the review, where the main active packaging strategies for preserving taste and aroma in fresh fruits are considered.

A further point concerns the distinction between volatile biosynthesis, volatile release, and volatile transport. In fruit packaging studies, transport is often discussed at the package level, referring to diffusion from fruit tissue to the headspace, partitioning between fruit matrix and package atmosphere, sorption by packaging polymers, and loss through film permeability. However, at the biological level, plant volatile emission is not necessarily a purely passive process: volatile compounds must cross membranes, the aqueous cell wall and, depending on the tissue, the cuticle before being released into the gas phase. Although direct evidence linking active packaging to volatile-transporter gene regulation in fresh fruits remains scarce, this mechanism highlights why packaging-induced changes in temperature, water status, membrane integrity, oxygen availability, and tissue physiology may influence not only volatile biosynthesis, but also volatile emission and availability in the package headspace [104,105]. The main deterioration processes affecting taste and aroma quality are summarized in Table 4, while Table 5 outlines the packaging-related mechanisms that may preserve or impair the fruit volatilome.

### 3.3. Consumer Perception and Sensory Validation of Packaged-Fruit Flavor

Instrumental volatile analysis is essential for understanding how packaging modifies fruit aroma, but GC-MS data alone cannot determine whether a packaged fruit will be accepted by consumers. Consumer perception depends on the integrated experience of appearance, texture, juiciness, sweetness, acidity, aroma intensity, aroma familiarity, freshness, aftertaste, serving temperature, and consumption context. A package may preserve or even increase the concentration of some volatiles, but still be judged negatively if the aroma is weak, unbalanced, fermented, artificial, sulfurous, medicinal, excessively herbal, or masked by active compounds. Conversely, measurable changes in volatile abundance may have little impact on acceptance if they remain below perceptual relevance or do not alter the overall sensory identity expected by consumers [1,2,3,14,17,23].

The sensory relevance of volatile compounds should also be interpreted using odor thresholds and, whenever possible, odor activity values. A compound detected by GC-MS is not necessarily relevant to perceived fruit aroma if its concentration remains below its odor threshold, whereas a compound present at low concentration may strongly affect aroma when its threshold is very low. This is particularly important for esters, aldehydes, lactones, terpenes, norisoprenoids, sulfur-containing compounds, and fermentative markers such as ethanol, acetaldehyde, and ethyl acetate. However, odor thresholds are not fixed universal values and should be used as interpretative references rather than absolute cut-off values. Supplementary Table S3 summarizes indicative odor-threshold values and sensory descriptors for selected compounds relevant to fresh-fruit active packaging [23,24,25,26].

Consumer-oriented sensory testing should complement volatilome profiling and trained-panel evaluation. Descriptive analysis is useful for identifying which attributes change, such as fruitiness, freshness, green notes, fermented notes, sulfurous notes, essential-oil notes, sweetness, sourness, or aftertaste. Consumer acceptance tests address a different question: whether those changes affect liking, preference, purchase intention or willingness to consume. Hedonic scales, paired-preference tests, purchase-intention questions, check-all-that-apply methods, just-about-right scales, and penalty analysis can help determine whether packaging-induced aroma changes are commercially relevant [1,2,3,23,24,25,26,45,46,47].

The interpretation of consumer tests should also consider visual and contextual bias. In fresh fruits, appearance often shapes expectation before aroma and taste are evaluated. Packaging cues may also influence perceived freshness, naturalness or safety, especially when active labels, pads, sachets, coatings, or strong aroma-emitting systems are visible to consumers. Therefore, studies evaluating flavor-oriented active packaging should specify whether consumer tests were blind or informed, whether visual appearance was controlled, whether the fruit was served at refrigeration or consumption temperature, and whether consumers evaluated aroma separately from overall liking [45,46,47].

The disconnect between visual shelf life and sensory shelf life should be quantified explicitly whenever possible. A practical metric is the visual–sensory shelf-life gap, defined as the difference between the last storage day on which the fruit remains visually marketable and the last storage day on which aroma, taste, or consumer acceptance remain within an acceptable range. This gap can be expressed in days, as a percentage of the total storage period, or through paired endpoints such as decay reduction versus off-flavor intensity, firmness retention versus ester loss, color retention versus consumer liking, or marketability score versus odor activity values for fermentative or sulfurous markers. Table 6 summarizes representative case types in which this mismatch should be quantified in active-packaging studies [12,13,14,17,23,24,25,26].

## 4. Preservation of Taste and Aroma in Fresh Fruits by Active Packaging

To avoid a subjective classification of active-packaging systems, this section uses a three-level framework. First, technologies are classified according to their primary active function, such as gas regulation, antimicrobial action, ethylene control, SO_2_ emission, ethanol emission, O_2_ scavenging, CO_2_ regulation, moisture adsorption, or multi-active intervention. Second, each system is described according to its material or format, including polymeric film, coating, sachet, pad, label, tray, paper, cardboard, edible coating, or multilayer structure. Third, the release, absorption, or interaction mechanism is specified, including passive atmosphere equilibrium, active gas flushing, controlled release, encapsulation, cyclodextrin inclusion complexes, adsorption, scavenging, diffusion, humidity-responsive release, or direct surface interaction. Within this framework, passive MAP is considered a reference atmospheric strategy rather than active packaging in the strict sense, edible coatings are treated as packaging-adjacent systems, and mechanical-protection elements are considered package-design components unless they include an active release, absorption, or scavenging function [18,19,143,144,145]. This classification framework is summarized in Table 7.

The aroma-preserving function of active packaging is mainly determined by the interaction of six factors: fruit physiology, gas balance, temperature, packaging material design, humidity control, and active-agent kinetics. Fruit physiology includes species, cultivar, climacteric or non-climacteric behavior, maturity stage, respiration rate, ethylene sensitivity, and cut status. Gas balance regulates respiration, ester biosynthesis, fermentative metabolism, and microbial growth. Temperature controls respiration rate, film permeability, volatile release, and active-compound release. Material design determines permeability, water-vapor transfer, aroma scalping, and active-agent exposure. Humidity control affects condensation, microbial off-odors, juiciness, and aroma release. Active-agent kinetics determine whether antimicrobial compounds, scavengers, or emitters act within a sensory-compatible window [18,19,21,22,27,28,75,76,103].

The case studies discussed in this section are also interpreted according to fruit physiological behavior. In climacteric fruits, active packaging may influence flavor by delaying ethylene-dependent ripening, softening, and senescence, but excessive ethylene removal, low O_2_, or high CO_2_ can suppress desirable esters, lactones, and ripe-fruit volatiles. In non-climacteric fruits, packaging mainly acts by preserving the volatilome already established at harvest and by preventing water loss, decay, microbial off-odors, aroma scalping, or fermentative metabolism. Fresh-cut fruits represent a third practical category because cutting increases respiration, wounding, surface moisture, enzyme-substrate contact, and microbial susceptibility. Therefore, active-packaging examples are interpreted below not only by function and material, but also by fruit physiology, respiration rate, maturity stage, and cut status [20,21,22,33,48,49,50,51,52,67,106,107,108,109,110,111,112,168,169,170,171].

To avoid treating active packaging as a generic preservation category, the following subsections evaluate each technology through a flavor-oriented filter: which sensory deterioration pathway it targets, which taste- or aroma-relevant compounds it helps preserve or reduce, which negative sensory effects it may cause if poorly designed, and which analytical or sensory endpoints are needed to validate its performance [11,12,13,14,15,16,17,18,19,23,24,25,26].

The preceding section established that the packaging system is not a neutral container but an active determinant of the fruit volatilome, able to either preserve or degrade sensory quality depending on how its properties are matched to the physiology of the product (Section 3.2.4, Figure 2). The same variables that, when poorly adjusted, promote oxygen depletion, condensation, aroma scalping, and fermentative off-flavor formation can, when deliberately controlled, be turned into tools for maintaining the native aroma profile. Active packaging exploits this duality: instead of limiting the package to a passive barrier, it incorporates components that regulate the gaseous environment, remove or release specific compounds, or suppress the metabolic and microbial processes that drive aroma loss. This section therefore develops, as functional strategies, the mechanisms that Section 3.2 introduced as sources of risk and evaluates each of them by the criterion adopted throughout this review, their net effect on the volatilome and on the balance of taste-active compounds, rather than on shelf life judged by appearance alone.

A single principle underlies the whole section and follows directly from the fruit-dependent behavior illustrated at the end of Section 3.2.4, where the same active-packaging concept preserved sesquiterpenes in one fruit while promoting fermentative esters in another: every active function is double-edged. A modified atmosphere that slows senescence can, if oxygen falls too low, trigger the ethanol and acetaldehyde accumulation already described; an antimicrobial or ethylene-suppressing volatile that protects the fruit may itself add exogenous notes if released above its sensory threshold; and a gas emitter that controls decay can distort the sugar–acid balance. The mechanisms summarized at a conceptual level in Table 4 are examined here in detail. The volatilome-related endpoints appropriate for evaluating these systems are those already outlined in Table 2, while representative active-packaging systems, grouped by active function, fruit physiology, material or format, mechanism of action, and sensory outcome, are summarized in Table 8. The following subsections address packaging-material properties (Section 4.1), modified-atmosphere and active gas regulation (Section 4.2), antimicrobial active packaging (Section 4.3), ethylene management (Section 4.4), SO_2_ emission (Section 4.5), multi-active packaging (Section 4.6), and other gas-, moisture-, and packaging-adjacent systems (Section 4.7).

### 4.1. Packaging Material Properties as Determinants of MAP Performance and Flavor Preservation

Packaging material properties determine whether an active or modified atmosphere will preserve fruit flavor or unintentionally distort it. Film thickness, O_2_, and CO_2_ transmission, water-vapor transmission, perforation level, polymer polarity, crystallinity, headspace volume, and product load jointly determine the package atmosphere and the interaction of the material with volatile compounds. A material that slows respiration and water loss may still weaken aroma if it scalps esters, terpenes, or lactones, whereas breathable and hygroscopic cellulosic materials may buffer humidity and reduce condensation but provide less gas-barrier control. Therefore, flavor-oriented packaging design should report material type, thickness, OTR, CO_2_TR, WVTR, perforation, headspace volume, and product-to-package ratio [27,28,29,30,31,32,33,106,107,108,109,110,111,112].

Aroma scalping should be interpreted as a partitioning and mass-transfer process rather than as simple surface adsorption. In a sealed fruit package, aroma compounds are distributed among fruit tissue, juice or aqueous phase, package headspace, and packaging material. At equilibrium, this distribution can be described by polymer/headspace or polymer/food partition coefficients, or by solubility coefficients when the volatile is considered as a sorbate in the polymer phase. The rate at which equilibrium is approached depends on diffusion coefficients, film thickness, temperature, contact time, and volatile concentration or activity. A single universal adsorption coefficient cannot be assigned to esters or terpenes as broad families; the coefficient must be defined for a specific compound, polymer, matrix, or simulant, temperature, and concentration range [27,28,29,30,31,32].

From a materials-science perspective, aroma scalping depends on both volatile and polymer properties. Volatile compounds with higher hydrophobicity, suitable molecular size, and stronger compatibility with the polymer phase generally show greater sorption. Terpenes such as limonene and myrcene, and medium-chain esters such as ethyl caproate, are relevant because they can contribute strongly to fruit aroma even at low concentrations. At the polymer level, polarity, glass-transition temperature, crystallinity, density, free volume, chain mobility, orientation, additives, and multilayer structure influence both sorption and diffusion. Polyolefins such as PP may interact with hydrophobic aroma compounds, whereas polyester materials such as PET and PLA may behave as stronger aroma barriers under some conditions, depending on grade, crystallinity, temperature, and volatile activity [29,30,31,32]. The principal packaging-material variables are summarized in Table 9, while indicative O_2_/CO_2_ flavor-risk conditions for representative fruit contexts are presented in Table 10. Material-science descriptors and reported sorption/partition evidence for aroma scalping in PP, PET, and PLA are compiled in Supplementary Table S1.

### 4.2. Modified Atmosphere Packaging and Active Gas Regulation

Modified atmosphere packaging requires careful terminology in the context of active packaging. Passive MAP should not be considered active packaging in the strict sense because the modified atmosphere is generated by the interaction between fruit respiration and film permeability, without the intentional incorporation of an active component. Active MAP, in contrast, involves deliberate atmospheric intervention through gas flushing or active gas-regulating elements such as O_2_ scavengers, CO_2_ emitters or absorbers, ethylene scavengers, or moisture-regulating components. The interpretation of MAP effects differs between climacteric and non-climacteric fruits: in climacteric fruits, MAP may delay ripening and ethylene-related volatile formation, whereas in non-climacteric fruits it mainly preserves an aroma profile already established at harvest and reduces decay, water loss, and fermentative off-flavor risk [75,76,111,112,182,183,184,185].

MAP modifies the gaseous environment surrounding the fruit, usually by reducing O_2_ and increasing CO_2_. In passive MAP, the equilibrium atmosphere is generated by respiration and film permeability. In active MAP, a defined atmosphere is introduced by gas flushing or by active gas-regulating elements. MAP can reduce respiration, delay senescence, limit microbial growth and maintain firmness and color [75,182,183,184,185]. From a flavor perspective, MAP must be understood as a metabolic regulator. O_2_ and CO_2_ affect respiration, fermentation, ethylene biosynthesis, ester biosynthesis, organic acid metabolism, sugar depletion, membrane oxidation, and microbial growth. Therefore, the optimal MAP for flavor preservation may be different from the optimal MAP for visual shelf-life extension.

Cozzolino et al. [20] evaluated fresh-cut ‘Big Top’ nectarines stored in air or active MAP. Using SPME-GC-MS, the authors showed that MAP influenced the volatile profile and sensory quality of nectarine slices after cold storage. Bovi et al. [12] showed that gas composition, condensation and membrane permeability influenced off-odor development and volatile accumulation in strawberries. Costa et al. [184] investigated passive and active MAP conditions for ready-to-eat table grapes, showing that packaging strategy influenced quality preservation. Kumar and Sethi [186] reported that MAP influenced shelf life and quality of fresh-cut apple wedges. Tinebra et al. [187] showed that different MAP conditions influenced mulberry fruit quality, including freshness-related attributes. Mulla et al. [188] reported that active MAP affected strawberry shelf life and quality parameters.

MAP can preserve taste by slowing respiration-related sugar and organic acid depletion. However, if atmosphere composition induces hypoxia, the resulting fermentative volatiles can dominate sensory perception even when soluble solids and acidity remain acceptable. Thus, MAP evaluation should include soluble solids, titratable acidity, pH, organic acid profile, sugar profile, sugar–acid ratio, ethanol, acetaldehyde, ethyl acetate, and sensory descriptors for sweetness, sourness, freshness, and fermented notes [12,14,72,189,190].

Super-atmospheric oxygen MAP has been proposed as an alternative for some whole and fresh-cut fruits and vegetables. It may suppress browning and microbial growth while avoiding severe hypoxia. However, very high oxygen can also accelerate oxidative reactions and modify volatile stability. Xu et al. [185] concluded that the effects of super-atmospheric oxygen MAP are product-dependent and require careful optimization.

### 4.3. Antimicrobial Active Packaging

Antimicrobial active packaging incorporates or releases compounds capable of inhibiting spoilage microorganisms. In fresh fruit applications, the most studied active agents include essential oils, plant extracts, organic acids, chitosan, antimicrobial peptides, volatile aldehydes, and encapsulated natural compounds [103,109,116,117,118,119,120,191,192,193,194]. Antimicrobial active packaging preserves flavor mainly by reducing spoilage-related volatile production. Fungal pathogens and spoilage yeasts can generate moldy, musty, fermented, acidic, and rotten notes. Inhibiting these microorganisms delays sensory rejection and preserves freshness. However, antimicrobial packaging may also introduce exogenous aroma, taste, or trigeminal sensations if the active compounds migrate or are released at excessive levels.

Active cardboard packaging containing β-cyclodextrin–essential oil inclusion complexes has been evaluated for grapes, nectarines, and lettuce. López-Gómez et al. [193] reported that active packaging reduced microbial growth and improved quality retention. In mandarins, active cardboard boxes with smart internal lining based on encapsulated essential oils enhanced shelf life [194]. Luesuwan et al. [116] reported that clove essential oil-fortified active packaging reduced fungal decay and preserved table grape quality. Rusková et al. [117] showed that biodegradable active packaging enriched with essential oils enhanced strawberry shelf life. Pinto et al. [119] reported that red thyme oil vapors reduced fungal decay and extended the shelf life of oranges. Kahramanoğlu [120] showed that lemongrass oil application combined with MAP improved postharvest life and quality of strawberries. Controlled release is critical for antimicrobial active packaging. Direct incorporation of essential oils into films can cause burst release, intense exogenous aroma, and rapid depletion. Encapsulation technologies, including cyclodextrin inclusion complexes, nanoemulsions, electrospun fibers, nanosponges, and multilayer structures, can regulate release and reduce sensory impact [109,121,122,123,124,125,126,127,128,129].

Cyclodextrins are particularly relevant because they form inclusion complexes with hydrophobic volatile molecules. Xiao et al. [122] reviewed cyclodextrins as carriers for volatile aroma compounds. Silva et al. [123] demonstrated that cyclodextrin nanosponges can encapsulate coriander essential oil for controlled-release active packaging. Shi et al. [124] prepared lemon essential oil/β-cyclodextrin inclusion complexes for blackberry preservation. Cao et al. [125] developed carvacrol β-cyclodextrin inclusion complexes and 1-MCP-α-cyclodextrin coated paper for peach preservation. Magri et al. [126] studied lemongrass essential oil encapsulation in cyclodextrins and maltodextrin. Xi et al. [127] and Yin et al. [128] showed that hydroxypropyl-β-cyclodextrin can form inclusion complexes with essential oil compounds and regulate their release kinetics. Ming et al. [129] reviewed rational design, molecular interactions, and activity assessment of essential oil encapsulation in cyclodextrins.

### 4.4. Ethylene Adsorption Packaging

Ethylene-regulating systems are primarily relevant to climacteric fruits and ethylene-sensitive commodities, where they can delay senescence and softening but may also suppress ripening-dependent aroma formation if applied too strongly or too early [153,154,155,156,157,158,159,160,161,162,163,164,165,166,167,168,169,170,171,172,173,174,175].

Ethylene is the central regulator of ripening and senescence in climacteric fruits and, consequently, a primary driver of both aroma development and aroma loss during postharvest life. At trace concentrations, it accelerates respiration, chlorophyll degradation, tissue softening, and membrane deterioration, and it governs the ester- and lactone-forming pathways that define ripe-fruit aroma, so that either its excess or its excessive suppression can degrade flavor [57,108]. Two complementary active-packaging strategies are used to limit its effect: removal of the ethylene accumulated in the package headspace (scavenging or adsorption) and suppression of the fruit’s own ethylene biosynthesis by volatiles released from the package [130]. From a flavor perspective, these routes are not equivalent. Scavenging withdraws an exogenous stimulus without adding volatiles to the headspace, whereas biosynthesis-suppressing systems based on essential oils simultaneously introduce aroma-active molecules, so that their sensory outcome is governed by the same sensory compatibility threshold discussed for antimicrobial active packaging (Section 4.3).

#### 4.4.1. Ethylene Scavenging and Adsorption Systems

Ethylene scavengers reduce the headspace ethylene concentration by chemical oxidation or physical adsorption, and are presented as sachets, as fillers dispersed in polymer films, or as coatings on paper and cardboard [131,132]. Potassium permanganate, which oxidizes ethylene to carbon dioxide and water, remains the most widely used chemical scavenger; because it is not food-contact-safe, it must be confined to sachets or immobilized on high-surface-area mineral supports [151,152]. The approach is long-established: Scott et al. [153] delayed banana ripening with permanganate in polyethylene bags, and Scott and Wills [154] reduced brown heart in pears by absorbing ethylene from the storage atmosphere. Álvarez-Hernández et al. reviewed the adsorbent materials available for this purpose [151] and, specifically, permanganate-based scavengers as active packaging [152], and then validated two mineral supports in fruit: a protonated montmorillonite loaded with potassium permanganate delayed senescence and preserved quality in blueberries [155], and a sepiolite-supported permanganate scavenger retained postharvest quality in apricots [156]. Comparable benefits have been reported with other carriers and fruits, permanganate-coated zeolite nanoparticles in ‘Golden Delicious’ apples [157], permanganate treatments in kiwifruit [158], permanganate–zeolite sachets in banana [159], and permanganate impregnated on silica–alumina derived from sugar cane bagasse ash, which delayed ripening and reduced ethylene production and weight loss in mangoes packed in cardboard boxes [160]. In all these systems, the support governs performance, since specific surface area and water availability determine both the oxidation rate and the risk of over-oxidation [151].

Physical adsorbents remove ethylene without chemical conversion. Activated carbon inside modified-atmosphere packages maintained tomato quality during cold storage [161], palladium-modified zeolites extended banana shelf life [162], and titanium dioxide coatings photocatalytically oxidize ethylene under ultraviolet illumination [163]. Halloysite nanotubes (HNTs) are particularly interesting for flavor-oriented packaging because their tubular lumen can retain ethylene while also acting as a reservoir for volatile actives. Tas et al. [164] dispersed HNTs in polyethylene to obtain nanocomposite films combining ethylene scavenging with an improved gas barrier, whereas Buendía-Moreno et al. [165] incorporated essential oils entrapped within cyclodextrins and/or HNTs into the coating of an active cardboard box for fresh tomato, so that the same nanomaterial provided both an ethylene sink and a controlled-release antimicrobial function.

The material format conditions the flavor outcome. When the scavenger is dispersed in a plastic film, as in the HNT/polyethylene nanocomposites above [164], or in the sachet-plus-LDPE systems that preserved banana and kiwifruit under modified atmosphere [166], the same barrier that retains the modified atmosphere also sorbs lipophilic esters and terpenes, a phenomenon known as flavor scalping that can measurably deplete the aroma of the packaged product [27]. When the scavenger is applied as a coating on paper or cardboard, the substrate is porous and hygroscopic, does not generate a strong modified atmosphere, but buffers condensation and interacts less strongly with fruity volatiles [165]. From the standpoint of aroma preservation, ethylene scavenging therefore has the distinctive advantage of being an essentially aroma-neutral intervention: it delays the senescence-driven decline of characteristic esters, lactones and terpenes, and the softening that governs their release, without adding exogenous odorants to the headspace. Its main limitation is that scavenging alone does not control fungal spoilage, so it is frequently combined with antimicrobial functions, as in the antifungal active packaging that associated thymol with an ethylene scavenger during storage of cherry tomatoes [167], a multi-active configuration further discussed in Section 4.6.

#### 4.4.2. Suppression of Ethylene Biosynthesis by Released Essential Oils

A mechanistically different route does not remove ethylene from the headspace but reduces its formation within the fruit. The reference technology for inhibiting ethylene action is 1-methylcyclopropene (1-MCP), which blocks ethylene receptors and has been widely adopted to preserve firmness and delay senescence [168]. Receptor blockade, however, also suppresses the ripening program that generates aroma. Yang et al. [169] showed that ethylene and 1-MCP regulate the major volatile biosynthetic pathways in apple, with 1-MCP down-regulating ester formation; the inhibition of alcohol acyltransferase, alcohol dehydrogenase, and lipoxygenase by 1-MCP has been repeatedly reported as the biochemical basis of this aroma loss [57]. The effect is documented across fruits: 1-MCP altered the volatile composition of ‘Packham’s Triumph’ pears during long-term storage [170] and modified the aroma compounds of ‘Big Top’ nectarines after shelf life [171]. This illustrates a general constraint on any ethylene-suppressing strategy: excessive inhibition of ripening preserves texture and acidity at the expense of the very esters and lactones that define ripe-fruit aroma, yielding firmer but blander fruit.

Released essential oils offer a milder and multifunctional alternative. Navarro-Martínez et al. [172] showed that essential oils emitted from active packaging strongly reduce ethylene biosynthesis in broccoli and apples, and López-Gómez et al. subsequently quantified the effect on the key biosynthetic enzymes, reporting reductions of up to about 50% in ACC synthase and ACC oxidase activity, with grapefruit and thyme essential oils the most effective in broccoli and tomato [173], and anise and lemon essential oils inhibiting ethylene production in blueberries and blackberries by 60–76%, even at an abusive temperature of 22 °C [174]. The same principle had been demonstrated earlier with active paper sheets carrying nanoencapsulated essential oils, which controlled ethylene production and maintained quality in flat peaches [175]. Unlike 1-MCP, these systems attenuate rather than abolish ethylene signaling, and they are almost always built on active paper sheets rather than high-barrier plastic films, so they combine ethylene suppression with the moisture-buffering behavior of the cellulosic substrate and avoid the flavor scalping associated with polymeric barriers [27].

The flavor balance of this route is more delicate than that of scavenging, because the released essential oils are simultaneously ethylene-suppressing, antimicrobial (Section 4.3), and aroma-active. Their benefit for aroma is twofold, delayed senescence and reduced microbial spoilage, but the same carvacrol, thymol, anise, or citrus volatiles can add herbal, phenolic, anise-like, or citrus notes if released above the sensory threshold. This is particularly critical in citrus, whose aroma is dominated by limonene, linalool, α-terpineol, and terpinen-4-ol, compounds proposed as quality-control parameters [176] and closely correlated with sensory quality [177], so that an exogenous citrus-type volatile may either reinforce or distort the native profile. The determining design variable is therefore the release rate, which must remain within the window bounded below by physiological and antimicrobial efficacy and above by sensory acceptability. López-Gómez et al. [33] characterized the release kinetics of carvacrol from active paper packaging under open- and closed-package conditions, providing the quantitative release data required to keep the emitted dose inside that window. Active paper sheets with nanoencapsulated essential oils have been validated in lemons, where they controlled microbial growth [178] and modulated the antioxidant system of the fruit [178], and essential oil vapors have also been shown to preserve quality in other highly perishable produce such as sliced mushrooms [179]. A further co-benefit is energetic: the antimicrobial and anti-ethylene action of released essential oils can reduce the refrigeration requirements of the cold chain [180].

A limiting case of this reasoning is the deliberate use of released volatiles to restore, rather than merely preserve, product aroma. Martínez-Hernández et al. [81] applied essential oil vapors at the end of the vacuum cooling of fresh culinary herbs and showed that the treatment promoted aromatic recovery, partially compensating the loss of characteristic volatiles caused by the cooling process. This result is conceptually important for the present review because it demonstrates that active packaging based on volatile release need not be a source of off-aromas: when the emitted volatiles are compatible with, or belong to, the product’s own aroma profile, controlled release can act as an aroma-positive intervention. It is the clearest counterpoint to the off-flavor risk that dominates the antimicrobial and biosynthesis-suppressing literature, and it reinforces the general criterion that the volatiles released by active packaging should, whenever possible, be selected among those naturally present in the target aroma.

### 4.5. SO_2_ Emission Active Packaging

SO_2_-emitting systems illustrate a different physiological context because they are mainly applied to non-climacteric, decay-prone fruits such as table grapes, where the main sensory objective is not to regulate ripening but to prevent fungal decay and the associated moldy, rotten, or fermented off-odors [34,35,36,37,38].

Sulfur dioxide (SO_2_) emission is the dominant active-packaging technology for table grapes, a non-climacteric fruit whose postharvest life is limited less by ripening metabolism than by gray mold caused by *Botrytis cinerea*, against which SO_2_ remains the standard control [34,35]. SO_2_-generating pads are cellulosic sheets containing sodium or potassium metabisulfite that react with the water vapor transpired by the fruit, so that the emission rate is intrinsically coupled to package humidity and temperature [34]. Commercial pads are engineered with two release phases: a fast initial burst that eradicates fungal spores on the berry surface during the first hours of storage, and a slow, sustained release that suppresses latent inoculum for up to two months [34]. Chaves Junior et al. [36] compared dual- and slow-release pads in clamshell-packaged ‘Benitaka’ grapes and showed that the active-ingredient load and the perforated liner jointly determine the control of gray mold, shattered berries and stem browning, and Higuchi et al. [37] demonstrated that combining a field ultra-fast pad applied before packaging with a dual-release pad during cold storage extends the shelf life of ‘Italia’ grapes while minimizing the total dose delivered to the fruit.

From a flavor standpoint, SO_2_ emission preserves the sensory identity of the bunch mainly by preventing the decay that would otherwise generate moldy, fermented and rotten off-odors, and by maintaining rachis freshness through its antioxidant action, which limits stem browning and the associated loss of visual and aromatic quality [34]. This protective effect, however, is bounded by a narrow tolerance window. Excess SO_2_ causes berry bleaching, shattered berries, darkening of the rachis and phytotoxicity, and, most relevant here, a pungent, sulfurous off-flavor that accompanies the irreversible damage observed in over-exposed berries [38], together with sulfite residues to which a fraction of consumers are hypersensitive [39]. Because release is governed by temperature and relative humidity, both of which fluctuate along the cold chain, the delivered dose is difficult to predict: insufficient release leaves the fruit unprotected, whereas excessive vaporization bleaches the berries and imparts off-flavor [39]. Regulatory limits reflect this risk, with the main import markets, including the European Union and the United States, setting a maximum residue tolerance of 10 ppm of SO_2_ in table grapes [34].

The material configuration is characteristic and instructive. The metabisulfite emitter is a paper-based sheet placed inside cardboard boxes together with perforated plastic liners, so that a cellulosic emitter and a polymeric retention barrier operate jointly: the liner retains the gas long enough for it to act, while its perforations prevent both saturation and excessive condensation [34,37]. The central design challenge is therefore not efficacy, which is well established, affordable, and safer than fungicide dipping [34], but the control of a reactive gas whose optimal concentration for decay suppression lies close to the threshold for bleaching, residue accumulation and sulfurous off-flavor. In this respect, SO_2_ emission poses the same quantitative problem as essential oil release (Section 4.4.2), matching a humidity- and temperature-dependent release rate to a narrow sensory window, and would benefit from the same kinetic characterization approach used for carvacrol release from active paper [33].

These limitations have driven a sustained search for gentler emitters. Ethanol vapor has been proposed as the leading alternative for grapes [39,133], and chlorine dioxide-releasing sachets and films have been developed for berries [35]; both are discussed in Section 4.7. The comparison is a useful illustration of the general principle of this review: an active gas that is highly effective against spoilage microorganisms is not necessarily neutral for flavor, and the selection of the emitted molecule should weigh its antimicrobial potency against its own sensory footprint.

### 4.6. Multi-Active Packaging

Multi-active packaging combines two or more functions in a single system. In fresh fruit packaging, the most relevant functions are modified atmosphere, antimicrobial release, antioxidant activity, ethylene regulation, moisture control, condensation control, and controlled release of volatile compounds. The rationale for multi-active packaging is that fruit flavor deterioration is multifactorial. MAP can delay respiration but may not control fungal spoilage. Antimicrobial packaging can suppress microorganisms but may not prevent fermentation. Moisture control can reduce condensation but does not regulate O_2_ and CO_2_. Ethylene regulation can delay senescence but may suppress desirable aroma formation. Therefore, multi-active packaging aims to control several deterioration pathways simultaneously [121,125,143,144,145].

Wang et al. [121] reviewed multilayer and composite films for active biodegradable packaging, highlighting the potential of layered structures to control release and improve functionality. Ahmed et al. [143] and Pereira de Abreu et al. [144] emphasized that active and intelligent packaging systems are increasingly designed to combine preservation, monitoring, and controlled interaction with the food environment. Kadirvel et al. [145] reviewed current active-packaging applications, prospects, and challenges and highlighted the need to match active systems with product-specific quality and safety requirements.

The combination of MAP with antimicrobial active packaging is particularly promising. MAP reduces respiration and senescence, while antimicrobial components suppress spoilage microorganisms. Mahecha-Rubiano et al. [195] developed a combined active packaging system with modified atmospheres and antimicrobial control for fresh strawberries, illustrating the growing interest in integrated systems. However, this combination must be optimized carefully because MAP can modify the volatility and partitioning of antimicrobial compounds, while high humidity can accelerate release from hydrophilic matrices.

Many natural antimicrobials also have antioxidant activity. Essential oils, phenolic extracts, and chitosan-based systems can reduce microbial growth and oxidative reactions [103,119,191,192]. However, antioxidant activity should not be interpreted as universally beneficial. Some lipid-derived aldehydes contribute positively to fresh green aroma. The objective is not to eliminate all oxidation-derived volatiles but to maintain a desirable balance among fruity, fresh, green, and ripe notes.

### 4.7. Other Active and Packaging-Adjacent Systems

The systems grouped in this section should be interpreted according to respiration rate, cut status and decay susceptibility rather than only according to climacteric classification, because ethanol emission, O_2_/CO_2_ regulation, and water-vapor adsorption often target high-respiring, fresh-cut, berry, or decay-prone commodities [39,133,134,135,136,137,138,181,182,183,184,185,186,187,188].

Beyond ethylene management, antimicrobial release and SO_2_ emission, a further group of active systems targets the gaseous and moisture environment of the package through the emission or removal of ethanol, oxygen, carbon dioxide, and water vapor [196]. In fresh fruit, these systems must be evaluated, as in the preceding sections, not only by their effect on decay and shelf life but by their consequences for the volatilome and the sugar–acid balance, since each of them can shift fruit metabolism towards or away from the fermentative pathways that generate off-flavors.

#### 4.7.1. Ethanol Emission

Ethanol vapor emission was developed largely as a gentler alternative to SO_2_ for decay-prone fruits. Ethanol is a generally-recognized-as-safe compound, and controlled-release sachets release ethanol vapor that inhibits *Botrytis cinerea* and other postharvest fungi. Lichter et al. [39] demonstrated that ethanol controls postharvest decay of table grapes while avoiding the bleaching and sulfite residues associated with metabisulfite pads, and Chervin et al. [133] reported that ethanol vapors limit Botrytis development over the postharvest life of table grapes; ethanol is accordingly listed among the main chemical and bio-based alternatives for Botrytis control in this crop [35]. Its flavor outcome is genuinely two-sided. At moderate doses, ethanol can be channeled into ester biosynthesis and has been reported to increase desirable aroma in fruits such as pear [197] and sweet cherry [198], and an ethanol-releasing sachet reduced decay while improving the aroma attributes of mulberry fruit [199], a rare example of an emitting system that enriches rather than masks the native aroma. At excessive doses, however, the same pathway accumulates ethanol, acetaldehyde, and ethyl acetate in the tissue, producing the alcoholic and solvent-like notes that define fermentative off-flavor [181]. As the emitter is typically enclosed within a plastic modified-atmosphere bag, the released dose accumulates in a low-exchange headspace and must be matched to the fermentative tolerance of the fruit.

#### 4.7.2. O_2_ Scavenging

Oxygen scavengers, most commonly iron-based sachets, remove residual and permeating oxygen from the package to suppress oxidative reactions and aerobic spoilage, and are well established for oxygen-sensitive foods [113]. In fresh respiring fruit, however, their use is constrained by the fruit’s own oxygen requirement. Beaudry [106] established that the benefits of reduced O_2_ partial pressure are bounded by a lower limit below which pyruvate metabolism shifts to fermentation; beyond that limit, alcohol dehydrogenase and pyruvate decarboxylase generate the ethanol, acetaldehyde, and ethyl acetate that dominate anaerobic off-flavor, the same mechanism that constrains modified-atmosphere packaging (Section 4.2). Oxygen absorbers are therefore used cautiously with whole fruit and more often in combination with other elements than alone: Aday et al. [110] combined oxygen and carbon dioxide absorbers to delay softening and mold development and extend the shelf life of strawberries, showing that the gain in firmness and decay control must be balanced against the risk of anaerobic off-flavor formation. From a flavor perspective, the value of O_2_ scavenging in fruit lies less in creating a near-anoxic atmosphere than in trimming the oxygen peaks that drive oxidative aroma degradation, while remaining above the fermentative threshold.

#### 4.7.3. CO_2_ Emission

Carbon dioxide emission raises the headspace CO_2_ concentration to inhibit microbial growth, an approach particularly suited to soft fruits such as strawberries and other berries, which tolerate, and benefit from, elevated CO_2_ levels that suppress Botrytis and slow respiration [110]. CO_2_ emitters are frequently paired with oxygen absorbers, since the volume contraction caused by oxygen removal is offset by CO_2_ generation while the accumulated CO_2_ adds an antimicrobial effect [110]. The flavor trade-off is well characterized in strawberry and closely mirrors that of high-CO_2_ modified atmospheres. Ke et al. [107] showed that the aroma of controlled-atmosphere strawberries is altered not only by the overproduction of acetaldehyde and ethanol but also by a reduced production of volatile esters, so that flavor is degraded from two directions at once. Larsen and Watkins [181] related off-flavor to increases in ethyl acetate and ethanol and identified atmospheres near 10% CO_2_ and 2% O_2_ as beneficial for firmness and ripening delay without off-flavor development, and Pelayo-Zaldívar et al. [114] found that the flavor life of strawberries, judged by aroma compounds, sugars, acids, and sensory evaluation, is shorter than the postharvest life judged by appearance, both in air and under CO_2_ enrichment. More recently, short-term high-CO_2_ treatment of ‘Seolhyang’ strawberries was shown to suppress the characteristic furanone while preserving the C6 aldehydes responsible for fresh notes and reducing acetaldehyde accumulation [115], confirming that the direction of the effect depends strongly on dose and duration. Where fruit respiration or CO_2_ sensitivity makes accumulation a hazard rather than a benefit, the complementary tool is CO_2_ scavenging, for which calcium hydroxide-based adsorbing films have been developed [134]. In both directions, the design objective is the same: to hold CO_2_ within the species-specific window that controls microorganisms without triggering CO_2_ injury, ester suppression, or fermentative off-flavor.

#### 4.7.4. Water Vapor Adsorption

Water vapor adsorption addresses a problem common to all packaged fruit: transpired and respired water accumulates in the headspace and condenses on the film and on the fruit surface, where it promotes fungal growth, tissue maceration, appearance defects, and, ultimately, flavor loss [135]. Plastic films have a water-vapor permeability far below the transpiration rate of most fresh produce, so they tend to trap condensation [135], and dedicated moisture regulators have been developed to counter this while maintaining the high relative humidity that fruit requires. Contact absorber pads remove liquid water only, which is insufficient because respiring fruit releases most of its water as vapor into the headspace [136]; non-contact systems therefore embed a hygroscopic salt or sugar between polymer layers. Rux et al. [137] developed humidity-regulating trays containing sodium chloride and validated them on strawberries and tomatoes, and Bovi et al. [138] characterized the moisture absorption kinetics of fructose-loaded pads, keeping the weight loss of packaged strawberries below 1% while minimizing in-package condensation. The material dimension connects directly to the paper-versus-plastic distinction that recurs throughout this review: the cellulosic substrate of active paper and cardboard is intrinsically hygroscopic and moderates in-package humidity as a co-benefit of its antimicrobial function. Active cardboard boxes with coatings of encapsulated essential oils have been shown to extend the shelf life of fresh tomato [139] and to preserve quality in bulk-packaged bell pepper [140] and nectarines [141], while active paper sheets have been validated in cherry tomato and kale [142]. Controlling condensation is, finally, an aroma-relevant intervention: by limiting the water film that both favors spoilage microorganisms and creates local anaerobic pockets, moisture regulation reduces the microbial and fermentative off-odors that would otherwise develop, complementing the temperature- and atmosphere-based strategies described in the preceding sections.

#### 4.7.5. Packaging-Adjacent Edible Coatings and Surface-Active Layers

Edible coatings should be distinguished from active packaging in the strict sense. They are not package-based systems because they are applied directly to the fruit surface and are consumed together with the product, whereas active packaging is normally associated with an external material, film, sachet, pad, label, tray, paper, cardboard, or multilayer structure that modifies the food-package environment. Nevertheless, edible coatings are considered here as packaging-adjacent systems because they can act through mechanisms directly relevant to flavor preservation: gas and water-vapor exchange, dehydration control, microbial colonization, controlled release of active compounds, and aroma retention at the fruit surface [19,147,148,149,150,151,152,183].

Edible coatings are not packaging in the strict sense, since they are applied to the fruit surface rather than to a container, but they share the two defining features of active packaging discussed above: they modify the gas exchange of the product and they act as reservoirs for the controlled release of active compounds, and they are therefore briefly considered here for completeness [196]. Chitosan-based matrices are the most studied: carvacrol-loaded chitosan nanoparticles maintained the quality of fresh-cut carrots [146], silver–chitosan nanocomposites applied as edible coatings affected the quality of fresh-cut melon [147]. Chitosan has also been used as a carrier for micronutrient fortification in fresh-cut produce [148]. From the perspective of aroma, the most relevant example is the combination of a carnauba wax coating with essential oils entrapped in β-cyclodextrins, which provides simultaneously a surface diffusion barrier and a controlled-release antimicrobial system in citrus fruit [149]; this configuration reproduces, at the fruit surface, the encapsulation-and-release logic that governs active paper and cardboard, and is subject to the same sensory constraint, since the coating places the aroma-active compound in direct contact with the tissue that will be consumed. Related encapsulation strategies have been applied to the sanitizing step rather than to the package [150], illustrating the continuity between surface treatments and packaging-borne release systems.

**Figure 3:**
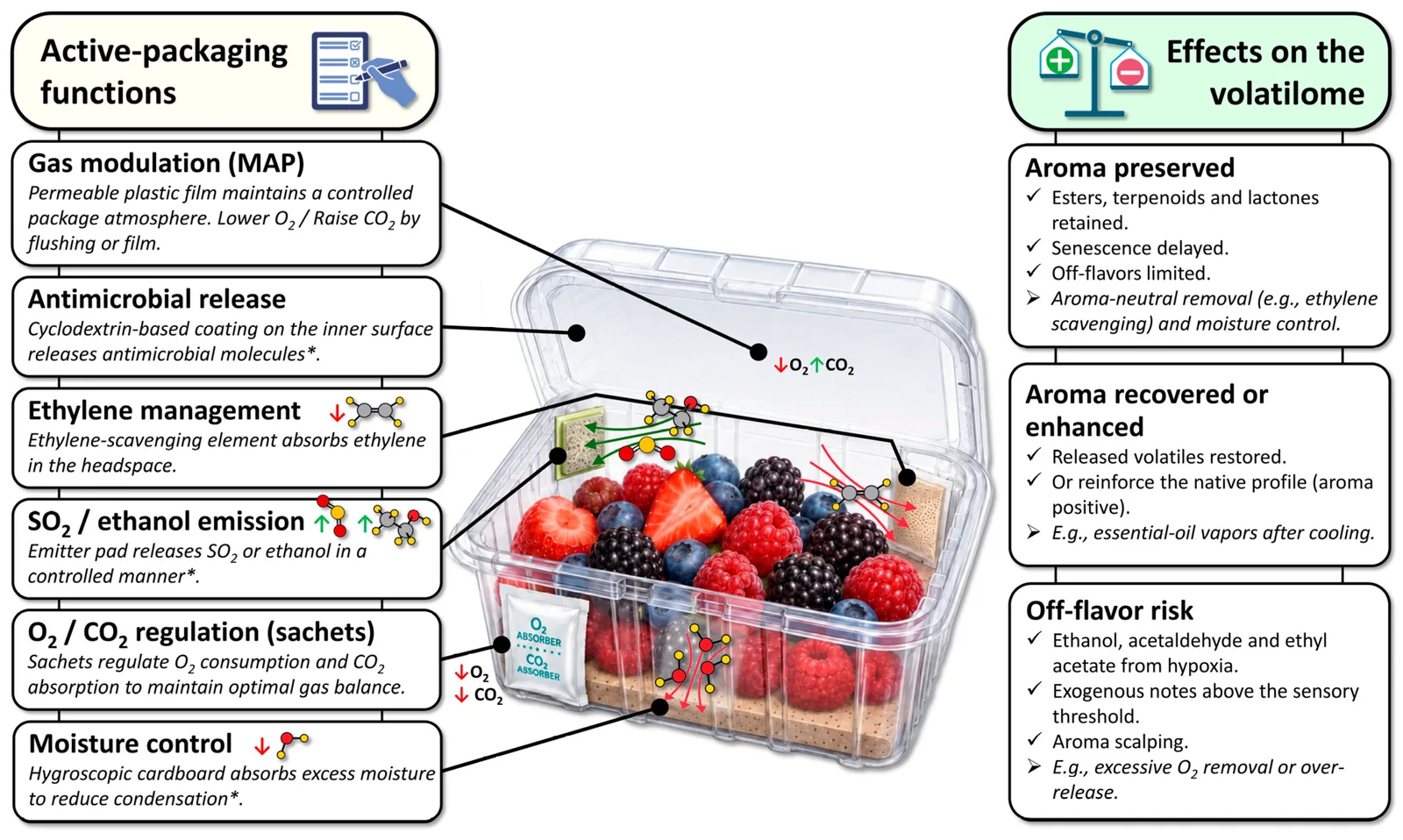
Active-packaging systems for the preservation of taste and aroma in fresh fruits and their effect on the volatilome. Active-packaging functions (**left**) act on the packaged-fruit volatilome (**center**), whose response is conditioned by the packaging material along a continuum from breathable, hygroscopic paper and cardboard to high-barrier polymeric films and PLA. Depending on how each function is matched to the fruit physiology, the outcome (**right**) ranges from aroma preservation, through the aroma-positive case in which released volatiles restore or reinforce the native profile, to off-flavor risk. Across all release-based systems, performance is bound by the sensory compatibility threshold. * Release of absorption kinetics may depend on temperature, relative humidity, packaging material and storage conditions.

Overall, the active-packaging strategies reviewed in this section demonstrate that the preservation of fruit flavor and aroma depends on achieving an appropriate balance between gas composition, moisture regulation, microbial control, ethylene management, and the controlled release or removal of active compounds. Modified-atmosphere systems, antimicrobial agents, ethylene scavengers, SO_2_ or ethanol-emitting devices, O_2_/CO_2_ regulators, moisture absorbers, and multi-active configurations may preserve desirable volatiles, delay senescence, and limit the formation of spoilage-related compounds. However, poorly adjusted atmosphere composition, excessive active-compound release, hypoxia, condensation, or aroma scalping by packaging materials may suppress native aroma, promote fermentative metabolites, and reduce consumer acceptance. Figure 3 integrates the main active-packaging functions discussed in this section and summarizes their potential beneficial and adverse effects on the fruit volatilome, emphasizing that successful packaging design requires matching the active mechanism, release kinetics, and material properties to the physiology and sensory tolerance of each fruit.

### 4.8. Advances in Flavor-Oriented Active Packaging During Storage

Recent advances in active packaging show a progressive shift from general shelf-life extension towards systems designed to preserve the sensory identity of fresh fruits during storage. These advances can be grouped according to the flavor-deterioration pathway they target: optimized MAP and gas-regulating systems to prevent fermentation without suppressing normal aroma biosynthesis; controlled-release antimicrobial systems to reduce microbial off-odor formation while limiting the sensory impact of the active compound itself; ethylene-regulating systems to delay senescence without completely suppressing ripening-related volatile formation; and active emitters or absorbers targeting O_2_, CO_2_, SO_2_, ethanol, and water vapor to regulate the microenvironment more precisely [18,19,20,21,22,103,145,191,192,193,194,195,196].

Multi-active systems and packaging-adjacent edible coatings are promising because flavor deterioration rarely has a single cause. Systems that combine gas regulation, antimicrobial release, ethylene control, moisture buffering, and material selection may better preserve flavor than technologies targeting only one route. However, the design criterion should remain sensory rather than merely technological: the most advanced package is not necessarily the one with the strongest antimicrobial, barrier, or anti-senescence effect, but the one that maintains the native volatilome, prevents off-flavor formation, and preserves consumer-relevant taste and aroma [139,140,141,142,195,196].

In the specific case of refrigeration-induced aroma weakening, controlled release of aroma-compatible volatiles may partially reinforce perceived aroma, but this should be distinguished from true restoration because irreversible chilling injury, senescence, substrate depletion, or microbial spoilage cannot be corrected simply by warming or by adding volatile compounds [14,15,16,81,180].

### 4.9. Safety, Migration and Regulatory Considerations for Active Packaging

Active packaging for fresh fruits should be evaluated not only through shelf-life extension, microbial control, or aroma preservation, but also through food-contact safety, migration, residue formation, and regulatory compliance. This is particularly important for systems that release active substances into the package atmosphere or onto the fruit surface, including essential oils, plant-derived volatiles, SO_2_ emitters, ethanol emitters, antimicrobial coatings, active papers, active cardboard, polymeric films containing active agents, and edible coatings. These systems should be validated within a safety window that includes technological efficacy, sensory compatibility, toxicological acceptability, food-contact compliance, and any applicable labelling or residue requirements [40,41,42,43,44].

Essential-oil-based active packaging illustrates this balance particularly well. Essential oils and individual volatile constituents may reduce microbial growth, delay senescence or suppress ethylene-related processes, but they are also odor-active. Their natural origin should not be interpreted as automatic regulatory or sensory acceptability. Studies should identify active compounds, purity, carrier, dose, release kinetics, headspace concentration, residue or migration level, food-contact status, and expected consumer exposure [40,41,42,43,44,103,145,191,192,193,194,195,196].

SO_2_-emitting systems require a similarly explicit safety interpretation. In table grapes, SO_2_-generating pads suppress Botrytis and prevent moldy or fermented off-odors, but excessive exposure may cause berry bleaching, rachis darkening, tissue damage, sulfurous off-flavor, and residue accumulation. Therefore, SO_2_ systems should combine decay control, sensory descriptors, residue monitoring, release kinetics, and compliance with the target-market requirements [34,35,36,37,38,39,40,41,42,43,44]. The main safety, migration, and regulatory checkpoints for these systems are summarized in Table 11.

### 4.10. Critical Summary of Potential Negative Effects of Active Packaging on Fruit Flavor

Active packaging should not be interpreted as intrinsically beneficial for fruit flavor. Its effect depends on the balance between preservation mechanism, fruit physiology, package material, storage temperature, humidity, gas composition, release or absorption kinetics, and consumer sensory expectations. A technology that extends visual shelf life, reduces decay, or delays senescence may still reduce eating quality if it suppresses desirable volatile biosynthesis, removes key aroma compounds from the headspace, releases an incompatible active aroma, induces anaerobic metabolism, or creates a sensory profile inconsistent with the native fruit identity [11,12,13,14,15,16,17,18,19,27,28,29,30,31,32,40,41,42,43,44,103].

The most frequent negative effect is atmosphere imbalance. MAP, O_2_ scavengers, CO_2_ emitters, or high-barrier materials may reduce respiration and microbial growth, but excessive O_2_ depletion or CO_2_ accumulation can trigger fermentative metabolism and increase ethanol, acetaldehyde, ethyl acetate, and other solvent-like or alcoholic notes. A second negative effect is selective aroma loss through polymer scalping. A third risk concerns active-compound release, especially essential oils, ethanol, and SO_2_. Finally, active packaging may maintain firmness, color, or decay control while sensory shelf life has already ended [11,12,13,14,15,16,17,27,28,29,30,31,32,34,35,36,37,38,39,40,41,42,43,44,133]. These potential negative effects and the corresponding control parameters are summarized in Table 12.

### 4.11. Comparative Effectiveness of Active-Packaging Technologies for Flavor Preservation

The relative effectiveness of active-packaging technologies for preserving fruit taste and aroma cannot be ranked using a single universal criterion. The most appropriate technology should be selected according to the dominant sensory-failure pathway of each fruit: respiration and fermentative metabolism, microbial off-odor formation, ethylene-driven senescence, water loss or condensation, aroma scalping, oxidative reactions, or active-compound masking. Table 13 ranks technologies by application scenario rather than absolute superiority [18,19,33,34,35,36,37,38,39,40,41,42,43,44,103,106,107,108,109,110,111,112,133,145,191,192,193,194,195,196,197,198,199].

### 4.12. Kinetic Matching Between Active Release, Fruit Respiration, and Flavor Evolution

Active-packaging systems should be designed according to kinetic compatibility, not only according to active-agent identity or total loading. In fresh fruits, the package environment changes continuously because the product consumes O_2_, produces CO_2_, releases water vapor, emits ethylene, modifies its volatile profile, and may progressively develop microbial or fermentative metabolites. Therefore, the active component must act at a rate that matches the physiological and sensory trajectory of the fruit [23,33,34,35,36,37,38,39,40,41,42,43,44,74,103,133,134,135,136,137,138,172,173,174,175,176,177,178,179,180].

Release-based antimicrobial systems must remain between two limits: the minimum concentration needed for antimicrobial or physiological efficacy and the sensory/safety threshold above which the active compound masks the native aroma or becomes unacceptable. Gas-regulating systems require matching O_2_ removal, CO_2_ buffering, and ethylene control to respiration rate, maturity, temperature, and tolerance limits. SO_2_ and ethanol emitters illustrate the need to synchronize active release with humidity, temperature, and decay pressure [33,34,35,36,37,38,39,133]. The kinetic relationships that should be matched between active-packaging function, fruit physiology, and flavor preservation are summarized in Table 14.

## 5. Study Limitations, Methodological Quality Appraisal, and Future Perspectives

### 5.1. Methodological Quality Appraisal of Packaging-Induced Odor and Flavor Studies

Because this review focuses on taste, aroma, and volatilome preservation rather than on packaging performance alone, the methodological quality of the original studies was interpreted using a flavor-oriented appraisal framework. This was especially important for studies addressing packaging-induced odor, off-flavor formation, aroma scalping, microbial volatile production, fermentative metabolism, or exogenous active-compound masking. General postharvest indicators such as color, firmness, weight loss, decay incidence, and microbial counts are relevant, but they do not by themselves demonstrate preservation of fruit aroma or taste. For this reason, studies were considered stronger when they integrated packaging characterization, headspace gas evolution, volatile profiling, sensory validation, and physiological or microbial interpretation [200,201]. The methodological quality evaluation form used for this appraisal is provided in Supplementary Table S2.

### 5.2. Study Limitations

The first limitation of the available literature is commodity specificity. Fruit species, cultivar, maturity stage, climacteric behavior, cut status, respiration rate, and microbial ecology strongly influence packaging response, so conclusions obtained for one fruit cannot be transferred directly to another. A second limitation concerns heterogeneous packaging descriptions: many studies report the active agent but not film thickness, permeability, perforation, headspace volume, product load, humidity profile, or release/absorption kinetics [1,2,3,4,5,6,7,8,9,10,11,12,13,14,15,16,17,18,19,20,21,22,23,24,25,26,27,28,29,30,31,32,33,40,41,42,43,44,45,46,47,103,106,107,108,109,110,111,112,200,201].

A specific source of bias concerns the sensory method used to validate packaging performance. Descriptive sensory analysis and consumer acceptance tests should not be interpreted as interchangeable endpoints. Descriptive analysis is appropriate for detecting attributes such as fruitiness, freshness, fermented notes, sulfurous notes, essential-oil aroma, sweetness, acidity, bitterness, astringency, and aftertaste. Consumer tests provide information on liking, preference, purchase intention, or willingness to consume, but they are less diagnostic mechanistically. Therefore, sensory evidence should be interpreted according to the method used, panel type, descriptors, and acceptance endpoint [45,46,47].

### 5.3. Future Perspectives

Future evaluations of active packaging for fresh fruits should integrate conventional quality measurements with volatilome-oriented and sensory endpoints. Standard reporting should include fruit species, cultivar, maturity stage, climacteric behavior, cut status, package material, thickness, permeability, headspace, active-agent loading, release kinetics, O_2_/CO_2_ evolution, RH, condensation, storage temperature, microbial counts, and sensory validation [23,24,25,26,33,45,46,47,106,107,108,109,110,111,112,200,201].

Future research should also integrate advanced analytical platforms capable of linking packaging design with molecular, metabolic, and sensory outcomes. Targeted and untargeted volatilomics should be combined with metabolomics, transcriptomics, and, when appropriate, proteomics or microbiome analysis to clarify how active packaging modifies precursor availability, enzyme activity, ethylene-related signaling, fermentative metabolism, microbial volatile production, and odor-active compound formation. GC-MS remains essential for volatile identification and quantification, but GC-olfactometry should be incorporated when possible to determine which compounds are odor-active. Electronic nose systems can provide rapid, non-destructive monitoring when coupled with chemometrics or machine-learning models [6,7,23,24,25,26,45,46,47,54,87].

Future studies should report odor thresholds or odor activity values for key fruit-derived volatiles, active-agent volatiles, and off-flavor markers, because concentration data alone may overestimate abundant but weakly odor-active compounds and underestimate trace compounds with very low sensory thresholds. Future studies should also model active-agent release, absorption, or scavenging curves together with fruit respiration, ethylene production, humidity evolution, and volatile formation, rather than reporting only the initial active-agent loading or end-point shelf-life data [23,24,25,26,33,34,35,36,37,38,39,40,41,42,43,44,45,46,47,133,134,135,136,137,138,200,201].

To make these future directions operational, research on flavor-oriented active packaging should be organized around a limited number of actionable priorities. Table 15 summarizes five priorities: standardized reporting, kinetic matching between fruit physiology and active-agent release or absorption, sensory-relevant volatilome validation, integration of trained-panel and consumer-oriented sensory tests, and validation under realistic supply-chain scenarios [23,24,25,26,33,45,46,47,106,107,108,109,110,111,112,200,201].

## 6. Conclusions

Active packaging should be evaluated not only as a technology for extending the visible shelf life of fresh fruits, but also as a tool that can preserve or distort their sensory identity. The fruit volatilome provides a useful framework because it connects ripening physiology, package atmosphere, microbial activity, material interactions, and consumer perception. A fruit may remain visually acceptable while its characteristic aroma has already weakened or while fermentative, microbial, sulfurous, oxidized, or exogenous notes have begun to dominate the sensory profile [1,2,3,4,5,6,7,8,9,10,11,12,13,14,15,16,17,18,19,23,24,25,26].

The central conclusion of this review is that every active-packaging function has a dual effect on flavor. MAP and gas-regulating systems can slow respiration and senescence, but excessive O_2_ depletion or CO_2_ accumulation may induce ethanol, acetaldehyde, and ethyl acetate formation. Antimicrobial systems can reduce spoilage-related off-odors, but essential oils or other released compounds may mask the native aroma if their concentration exceeds the sensory compatibility threshold. Ethylene-scavenging systems may delay senescence in climacteric fruits, but excessive suppression of ripening can reduce ester and lactone formation. SO_2_ and ethanol emitters can control decay in susceptible fruits such as table grapes, but they may generate sulfurous or alcoholic off-notes if release kinetics are not well matched to the product [11,12,13,14,15,16,17,18,19,33,34,35,36,37,38,39,40,41,42,43,44,103,106,107,108,109,110,111,112,133,153,154,155,156,157,158,159,160,161,162,163,164,165,166,167,168,169,170,171,172,173,174,175,176,177,178,179,180].

Optimization should therefore be commodity-specific rather than technology-driven. The packaging system must be selected according to fruit species, cultivar, maturity stage, respiration rate, climacteric or non-climacteric behavior, cut status, storage temperature, expected temperature fluctuations, headspace volume, and product load. The same active-packaging strategy may have different sensory outcomes in climacteric, non-climacteric, fresh-cut, and overripe fruits, and its interpretation should always consider fruit physiology, maturity stage, and cut status [20,21,22,33,48,49,50,51,52,67,106,107,108,109,110,111,112,153,154,155,156,157,158,159,160,161,162,163,164,165,166,167,168,169,170,171,172,173,174,175].

Future evaluations should integrate conventional quality measurements with volatilome-oriented and sensory endpoints. Future validation should further combine targeted and untargeted volatilomics, GC-olfactometry, electronic nose systems, multi-omics approaches, and chemometric modeling with descriptive sensory analysis and consumer acceptance tests. Consumer-oriented validation is particularly important because preservation of volatile markers does not necessarily imply liking, purchase intention, or acceptance if the perceived aroma becomes weak, unfamiliar, masked, or inconsistent with the expected fruit identity [23,24,25,26,45,46,47,87,200,201].

Reporting the visual-sensory shelf-life gap would help distinguish packages that genuinely preserve eating quality from those that mainly delay visible deterioration. Refrigeration-induced aroma loss should also be interpreted according to reversibility: physical reduction in volatile release may be partly recovered by tempering, whereas chilling injury, senescence, microbial spoilage, aroma scalping, or substrate depletion require preventive packaging design rather than post-storage correction. Matching the kinetics of active release, absorption, or scavenging with fruit respiration, humidity generation, and flavor evolution is essential to avoid technologies that are microbiologically effective but sensorially damaging [12,13,14,15,16,17,23,24,25,26,27,28,29,30,31,32,74,77,78,79,81].

## Figures and Tables

**Table 1:** Main biochemical routes involved in the formation of taste and aroma during fruit ripening.

Biochemical Route or Sensory Component	Main Precursors	Representative Compounds	Typical Sensory Contribution	Fruit Examples	Relevance for Packaged Fruits	References
Sugar metabolism	Glucose, fructose, sucrose, sorbitol	Soluble sugars	Sweetness and flavor balance	Apple, pear, grape, peach, citrus, berries	Determines baseline acceptability before packaging	[1]
Organic acid metabolism	Malic acid, citric acid	Organic acids	Sourness, freshness, sweetness modulation	Citrus, pineapple, apple, peach, grape, strawberry	Sugar/acid balance may shift during storage	[68]
Phenolic metabolism	Flavonoids, tannins, phenolic acids	Catechins, anthocyanins, tannins	Bitterness, astringency, mouthfeel	Grape, persimmon, pomegranate, apple peel, berries	Oxidation or tissue damage may modify mouthfeel	[48]
Fatty-acid/lipoxygenase pathway	Linoleic and linolenic acids	Hexanal, (E)-2-hexenal, hexanol, (Z)-3-hexenol	Green, grassy, fresh-cut notes	Apple, tomato, melon, pear, strawberry	Sensitive to oxygen availability, tissue damage, and storage temperature	[55]
Ester biosynthesis	Alcohols and acyl-CoA substrates	Ethyl butanoate, ethyl hexanoate, hexyl acetate, isoamyl acetate	Fruity, sweet, banana-like, apple-like notes	Apple, banana, strawberry, melon, pear	Can be reduced by low temperature, ethylene inhibition, or unsuitable atmospheres	[57]
Amino-acid-derived volatiles	Leucine, isoleucine, valine, phenylalanine	2-Methylbutanal, 3-methylbutanol, phenylacetaldehyde, 2-phenylethanol	Fruity, malty, floral, honey-like notes	Melon, banana, tomato, apple, strawberry	Hypoxia and microbial activity may shift desirable notes towards off-flavors	[62]
Sulfur-containing compounds	Methionine, cysteine	Methionol and other sulfur volatiles	Tropical or varietal notes at trace levels; sulfury notes in excess	Melon, citrus, tropical fruits	May become problematic under low oxygen or microbial spoilage	[62]
Terpenoid pathway	Isoprenoid precursors	Limonene, linalool, geraniol, citral	Citrus-like, floral, fresh, resinous notes	Citrus, grape, mango, melon, berries	Retention depends on film permeability and headspace partitioning	[54]
Carotenoid cleavage	Beta-carotene, lycopene, zeaxanthin	Beta-ionone, beta-damascenone, geranylacetone	Floral, violet-like, fruity, tea-like notes	Tomato, grape, watermelon, melon	Linked to maturity, pigment metabolism and storage stress	[63]
Glycosidically bound aroma pool	Glycosylated terpenes, alcohols, phenols, and norisoprenoids	Linalool, geraniol, benzyl alcohol, 2-phenylethanol after hydrolysis	Latent floral, fruity or varietal aroma	Grape, mango, citrus, tamarillo, kiwifruit	Atmosphere and enzyme activity may affect release of bound aroma	[66]

**Table 2:** Volatilome-related variables that should be considered when evaluating active packaging for fresh fruits.

Quality Dimension	Conventional Measurement	Volatilome-Related Complement	Interpretation for Active Packaging	References
Visual shelf life	Color, browning, decay incidence	Volatile markers of senescence or microbial spoilage	Visual acceptability may persist after aroma decline	[14]
Texture	Firmness, softening rate, juiciness	Volatiles linked to tissue disruption and lipid oxidation	Mechanical damage may increase green or oxidized notes	[71]
Taste balance	Soluble solids, titratable acidity, sugar/acid ratio	Interaction between sugars, acids and aroma-enhancing volatiles	Sweetness perception may depend on volatile preservation	[1]
Aroma preservation	Total volatile abundance	Key odor-active compounds and odor thresholds	Total VOCs alone may not predict sensory quality	[23]
Off-flavor risk	Sensory panel descriptors	Ethanol, acetaldehyde, ethyl acetate, sulfur-like compounds	Indicates hypoxia, fermentation, or microbial activity	[17]
Packaging performance	Gas composition, weight loss, microbial counts	Fruit tissue/headspace volatile partitioning	Film permeability and active components may reshape aroma release	[12]
Maturity stage and ripening status	Harvest maturity index, color, firmness, soluble solids, titratable acidity, starch index, ethylene production, or respiration rate	Maturity-dependent esters, lactones, terpenes, aldehydes, alcohols, sulfur volatiles, glycosidically bound precursors, and fermentative markers	Immature, mature-firm, ready-to-eat, and overripe fruits may respond differently to the same package; active packaging preserves or modulates an existing sensory potential but cannot fully compensate for inadequate ripening	[48,52,67]
Sensory and consumer validation	Descriptive analysis, hedonic liking, preference tests, purchase intention, CATA, JAR scales, penalty analysis, and willingness-to-consume questions	Attribute intensity, perceived fruitiness, freshness, fermented notes, sulfurous notes, essential-oil notes, aroma masking, liking and rejection thresholds	GC-MS and total VOCs do not necessarily predict consumer acceptance; trained panels explain sensory mechanisms, whereas consumer tests determine market acceptance	[1,2,3,14,17,23]
Odor-threshold relevance	Total volatile concentration or relative peak area	Odor threshold, odor activity value, descriptor, matrix-specific threshold source and concentration range for key odorants	High abundance does not necessarily imply sensory relevance; trace compounds with very low thresholds may dominate aroma perception	[23,24,25,26]

**Table 3:** Reversibility of aroma loss after refrigerated storage in fresh fruits.

Aroma-Loss Scenario	Main Mechanism	Degree of Reversibility	Practical Implication for Active Packaging	References
Reduced aroma intensity immediately after cold removal	Lower vapor pressure and reduced volatile release from tissue to headspace and nasal cavity	Potentially reversible	Tempering at consumption temperature may increase perceived aroma if volatile compounds remain present in the tissue	[14,48,74]
Slowed ripening-related volatile biosynthesis	Reduced enzymatic activity and temporary slowing of ethylene- or ripening-related metabolism	Partially reversible	Recovery depends on maturity stage, fruit physiology, storage duration and whether the fruit remains metabolically competent	[14,15,16,74]
Cold-induced reduction of ester formation	Suppression of alcohol acyltransferase, alcohol dehydrogenase, lipoxygenase-related pathways or substrate availability	Partially reversible to poorly reversible	Recovery is more likely after short cold exposure and less likely after prolonged chilling or senescence	[15,16,57]
Chilling injury in sensitive fruits	Membrane dysfunction, oxidative stress, lipid-metabolism disruption, impaired energy status and ripening disorder	Largely irreversible	Active packaging cannot fully restore aroma once physiological injury has damaged volatile-biosynthesis pathways	[77,78]
Prolonged storage or senescence	Substrate depletion, cell-wall breakdown, loss of metabolic coordination, and accumulation of stress volatiles	Mostly irreversible	Warming may increase emission of remaining volatiles, but may also intensify stale, overripe, or fermented notes	[14,17,74]
Fresh-cut tissue damage under refrigeration	Wounding, exposed surfaces, leakage, enzyme-substrate contact, and microbial susceptibility	Poorly reversible	Packaging should prevent further deterioration rather than assume post-refrigeration aroma recovery	[71,72,75,79]
Microbial or fermentative off-flavor after cold-chain failure	Yeast, bacterial, or mold metabolism; ethanol, acetaldehyde, ethyl acetate, acids or sulfur-like volatiles	Irreversible from a sensory-quality standpoint	Tempering may intensify off-odor perception; active packaging must prevent rather than correct this route	[17,23,75,76,82,83,84,85,86,87]
Aroma scalping by packaging polymers	Partitioning of esters, terpenes, aldehydes or lactones into the packaging material	Limited and material-dependent	Recovery depends on polymer-volatile partitioning and may not occur during normal consumption; material selection is preventive	[27,28,29,30,31,32]
Controlled release of aroma-compatible volatiles	Addition or reinforcement of volatiles compatible with the native aroma profile	Compensatory rather than fully restorative	May improve perceived aroma only if released compounds belong to, or are compatible with, the expected fruit aroma and remain below masking thresholds	[81]

**Table 4:** Main deterioration processes affecting taste and aroma quality in packaged fresh fruits.

Deterioration Factor	Main Mechanism	Volatilome Consequence	Taste/Texture Consequence	Typical Risk Conditions	Relevant Control Approach	References
Refrigerated storage	Reduced respiration and microbial growth, but possible chilling injury or suppression of aroma biosynthesis in sensitive fruits	Loss of esters, terpenoids or varietal volatiles; stress-related aldehydes or alcohols	Weak aroma, uneven ripening, mealiness, browning, or poor flavor recovery after cold storage	Storage below physiological tolerance threshold; prolonged refrigeration; chilling-sensitive commodities	Commodity-specific temperature control; avoidance of chilling ranges; volatile profiling after storage and after tempering	[14]
Room-temperature storage	Accelerated respiration, ethylene action, substrate depletion, and microbial growth	Initially higher volatile release followed by overripe, fermented, or stale notes	Sugar/acid imbalance, softening, water loss, reduced freshness	Retail display, cold-chain interruption, domestic storage, high product load	Temperature-stable package design; cold-chain control; temperature-abuse validation	[74]
Microbial deterioration	Growth of yeasts, molds, and bacteria using sugars, acids, and leaked cellular nutrients	Ethanol, higher alcohols, esters, acids, sulfur compounds, musty/earthy volatiles	Fermented, sour, vinegar-like, musty, or spoiled notes; tissue leakage and decay	Condensation, damaged tissues, fresh-cut surfaces, high water activity	Antimicrobial packaging; moisture control; hygienic processing; microbial and VOC monitoring	[82]
Enzymatic deterioration	Lipid oxidation, phenolic oxidation, cell wall degradation, and altered ester metabolism	Excess C6 aldehydes/alcohols, oxidized notes, altered ester balance	Browning, bitterness/astringency changes, softening, juiciness loss	Cutting, bruising, chilling stress, senescence	Balanced O_2_/CO_2_ atmosphere; antioxidant/anti-browning systems; control of tissue damage	[9]
Mechanical damage	Cell rupture, membrane disruption, substrate leakage, and increased respiration	Rapid wound-related C6 volatiles; later fermentative or microbial VOCs	Bruising, texture collapse, juice leakage, altered mouthfeel	Harvest, grading, transport vibration, compression, slicing	Structural protection; cushioning; suitable fill weight; reduced fruit movement inside package	[97]
Packaging imbalance	Inadequate film permeability, excessive O_2_ removal, high CO_2_, condensation, or active-compound mismatch	Ethanol, acetaldehyde, ethyl acetate, aroma scalping, masking by active volatiles	Visual shelf life may persist despite flavor failure	Poor film/commodity match; temperature abuse; excessive active release	Commodity-specific package design; headspace gas monitoring; sensory, and volatilome validation	[75]

**Table 5:** Packaging-related mechanisms that may preserve or deteriorate fruit volatilome.

Packaging Mechanism	Potential Benefit	Sensory Risk	Volatilome Markers to Monitor	Design Consideration	References
Reduced O_2_ atmosphere	Slows respiration, senescence, and oxidative reactions	Excessive O_2_ reduction may induce fermentation	Ethanol, acetaldehyde, ethyl acetate, ethyl esters	Match film permeability to fruit respiration, product load, and temperature	[106]
Elevated CO_2_ atmosphere	May reduce respiration and inhibit some spoilage organisms	Excessive CO_2_ may cause physiological stress or abnormal flavor	Fermentation markers, altered ester/alcohol balance	Define commodity-specific CO_2_ tolerance thresholds	[107]
Moisture control	Limits dehydration and preserves juiciness	Excess humidity or condensation favors microbial growth; excessive absorption causes dryness	Microbial VOCs, musty notes, sulfur-like compounds	Balance water-loss prevention with condensation reduction	[12]
Ethylene scavenging	Delays ripening, softening, and senescence	Excessive ethylene removal may suppress aroma development in climacteric fruits	Esters, lactones, ripening-associated volatiles	Adjust to maturity stage and desired ripening window [108]	[108]
Antimicrobial active compounds	Reduces microbial spoilage and microbial off-odors	Active compounds may mask native fruit aroma	Essential-oil markers, microbial VOCs, varietal aroma compounds	Control release rate and sensory compatibility with the fruit [109]	[109]
Oxygen scavenging	Limits oxidative reactions and may reduce browning	Can create hypoxia if excessive	Ethanol, acetaldehyde, ethyl acetate	Use only when respiration rate and package permeability are well defined	[110]
Aroma scalping by polymers	None from a sensory perspective, unless unwanted volatiles are removed	Loss of key odor-active compounds and distortion of aroma balance	Esters, terpenes, aldehydes, norisoprenoids	Select materials with low sorption of target aroma compounds	[27]
Active labels or emitters	Targeted antimicrobial or antioxidant action without direct food contact	Over-release may produce artificial, medicinal, spicy, or resinous notes	Active volatile compounds and native fruit volatiles	Validate release kinetics under refrigeration and temperature abuse	[33]

**Table 6:** Case-based framework for quantifying the disconnect between visual shelf life and sensory shelf life in packaged fresh fruits.

Case or Packaging Context	Visual or Conventional Shelf-Life Endpoint	Sensory or Volatilome Endpoint	Type of Visual-sensory Disconnect	Recommended Quantitative Expression	References
Restrictive MAP or active gas regulation in high-respiring fruits	Firmness, color and decay incidence may remain acceptable	Ethanol, acetaldehyde, ethyl acetate, and other fermentative markers may increase; fruity esters may decrease	Visual shelf life may exceed sensory shelf life when hypoxia develops before visible decay	Days of visual acceptability minus days before fermentative markers exceed threshold or off-flavor score becomes unacceptable	[12,14,17,75,76,111,112]
Strawberry or berry packages with atmosphere imbalance	Surface appearance and decay may be delayed under modified atmosphere	Hypoxic conditions may increase ethyl esters associated with off-flavor and reduce methyl/hexyl esters associated with typical fruity aroma	Visual preservation may coexist with loss of characteristic aroma	Percentage change in fruity esters and fermentative esters relative to control at the same visually acceptable storage day	[12,14,22,106,107,110,113,114,115]
Longan under active biopolymer-coated film	External quality and decay control may be improved	Off-flavor production may be reduced and sesquiterpenes better preserved	Visual and sensory shelf life may be aligned when antimicrobial, moisture, and atmosphere effects are balanced	Extension in visual shelf life together with retention percentage of key sesquiterpenes and sensory off-flavor score	[22]
Antimicrobial essential-oil active packaging	Decay incidence and microbial counts may decrease	Native aroma may be masked by herbal, spicy, medicinal, phenolic, resinous, or citrus-like active-agent notes	Microbial shelf life may improve while sensory shelf life decreases if active release exceeds compatibility threshold	Percentage decay reduction versus active-compound headspace concentration, sensory descriptor intensity and consumer liking	[22,81,103,108,109,116,117,118,119,120,121,122,123,124,125,126,127,128,129,130,131,132]
SO_2_-generating pads in table grapes	Gray mold control, rachis freshness, and marketable appearance may improve	Excess SO_2_ may cause sulfurous off-flavor, bleaching, tissue injury or sulfite-residue concerns	Visual decay control may be achieved at the expense of sensory or safety acceptability if release is excessive	Days of marketable bunch appearance versus SO_2_ residue, bleaching score, sulfurous descriptor intensity, and consumer rejection	[34,35,36,37,38,39,40,41,42,43,44]
Ethanol-emitting systems	Fungal decay may decrease and commercial shelf life may increase	Alcoholic, fermented, or overripe notes may appear if vapor concentration or residue is excessive	Decay suppression may not equal sensory preservation	Decay reduction percentage versus ethanol residue/headspace concentration and fermented-note intensity	[39,133]
Moisture absorbers and absorbent pads	Condensation and visible microbial growth may decrease	Excessive moisture removal may reduce juiciness, freshness perception, and aroma release	Visual cleanliness may improve while eating quality weakens through dryness	Condensation score or weight loss versus juiciness score, texture, aroma intensity, and liking	[134,135,136,137,138,139,140,141,142]
Polymeric films with aroma scalping potential	Film may maintain atmosphere, firmness and visual freshness	Esters, terpenes, aldehydes, or lactones may be selectively sorbed by the polymer, reducing aroma intensity	Visual shelf life may be extended while varietal aroma becomes weaker	Headspace recovery percentage of key odorants, partition coefficients, and sensory aroma-intensity score at visually acceptable time points	[27,28,29,30,31,32]
Refrigerated storage of chilling-sensitive fruits	Low temperature may preserve firmness and slow decay	Characteristic flavor volatiles or ester biosynthesis may decrease, producing weak aroma despite acceptable appearance	Temperature-based visual preservation may precede sensory deterioration	Days of firmness/color acceptability versus percentage reduction in key aroma volatiles and consumer flavor liking	[14,15,16,74,77,78,79,80]
Active packaging with visually positive but chemically incomplete evaluation	Color, firmness, weight loss and decay are improved	Volatilome, off-flavor markers or sensory descriptors are not measured	Sensory shelf life cannot be inferred from visual shelf life	Report as visual-only evidence; do not claim flavor preservation unless volatile and sensory endpoints are included	[13,14,72]

**Table 7:** Unified classification framework for flavor-oriented active packaging systems in fresh fruits.

Classification Level	Main Categories	Examples in Fresh-Fruit Packaging	Relevance for Aroma and Taste Preservation	References
Primary active function	Gas regulation; antimicrobial action; ethylene control; SO_2_ emission; ethanol emission; O_2_ scavenging; CO_2_ emission or absorption; moisture adsorption; multi-active intervention	MAP films; antimicrobial essential-oil systems; ethylene scavenger sachets; SO_2_ pads; ethanol emitters; O_2_ scavengers; CO_2_ regulators; absorbent pads; active cardboard systems	Determines the main deterioration pathway targeted: respiration, fermentation, microbial off-odors, senescence, condensation, oxidation, or decay-related aroma loss	[18,19,75,76,111,112]
Material or format	Polymeric film; biodegradable film; coating; sachet; pad; label; tray; paper; cardboard; edible coating; multilayer structure	PE, PP, PET, PLA and multilayer films; active labels; cardboard boxes; cyclodextrin-coated papers; SO_2_ pads; moisture-absorbing trays; chitosan or wax-based coatings	Determines gas transfer, water-vapor behavior, physical protection, aroma scalping, active-agent location, and consumer exposure to released compounds	[23,24,27,28,104,105,108,109,116,117,118,119,120,121,122,123,124,125,126,127,128,129,130,131,132,139,140,141,142,146,147,148,149,150]
Release, absorption or interaction mechanism	Passive equilibrium; gas flushing; diffusion; controlled release; encapsulation; inclusion complex; adsorption; chemical scavenging; humidity-responsive release; direct surface interaction	Cyclodextrin-essential oil complexes; ethylene scavengers; activated carbon or zeolite adsorbents; SO_2_ pads; ethanol emitters; O_2_ scavengers; humidity-regulating pads	Determines whether the system acts gradually, rapidly, locally or throughout the headspace and whether it risks burst release, hypoxia, CO_2_ injury, aroma masking or loss of odor-active volatiles	[33,34,35,36,37,38,39,81,106,107,108,109,110,113,114,115,116,117,118,119,120,121,122,123,124,125,126,127,128,129,130,131,132,133,134,135,136,137,138,139,140,141,142,143,144,151,152,153,154,155,156,157,158,159,160,161,162,163,164,165,166,167,168,169,170,171,172,173,174,175,176,177,178,179,180,181]
Sensory target	Retention of desirable odor-active compounds; reduction of fermentative markers; suppression of microbial volatiles; preservation of sugar/acid balance; prevention of exogenous aroma masking	Maintenance of esters, terpenes, lactones and varietal aroma; reduction of ethanol, acetaldehyde, ethyl acetate, sulfur-like compounds, or musty volatiles	Links the classification directly to flavor preservation rather than general shelf-life extension	[14,17,23,24,25,26]
Main sensory risk	Hypoxia; excessive CO_2_; aroma scalping; active-compound masking; sulfurous or alcoholic notes; excessive dehydration; condensation-driven microbial odor	Over-restrictive MAP; high-barrier films; poorly controlled essential-oil release; excessive SO_2_ or ethanol emission; inadequate humidity regulation	Critical criterion for determining whether an active system preserves or distorts sensory identity	[22,27,28,29,30,31,32,34,35,36,37,38,39,103,133,134,135,136,137,138,139,140,141,142]

**Table 8:** Representative flavor-oriented active-packaging systems for fresh fruits classified according to active function, fruit physiology, material, or format, mechanism of action and sensory outcome.

Active-Packaging Strategy	Fruit Physiology/Climacteric Behavior	Material or Format	Release/Absorption/Interaction Mechanism	Representative Fruit/Context	Main Aroma/Taste Benefit and Sensory Risk	References
MAP and active gas regulation	Climacteric, non-climacteric, fresh-cut, and high-respiring fruits	Permeable films, gas-flushed packages and gas-regulating components	Passive atmosphere equilibrium or deliberate O_2_/CO_2_ modification	Fresh-cut nectarine, strawberry, berries, apple, peach, tomato, and other packaged fruits	Can slow respiration, senescence, and microbial growth when O_2_/CO_2_ remain within fruit-specific limits; may induce ethanol, acetaldehyde, and ethyl acetate if hypoxia develops	[75,78,79,111,112]
Antimicrobial active packaging	Non-climacteric and highly perishable fruits; fresh-cut commodities; climacteric fruits when microbial risk dominates	Films, labels, papers, cardboard, coatings, sachets, or inner-surface active layers	Controlled release, encapsulation, cyclodextrin inclusion, diffusion, or surface interaction	Berries, citrus, fresh-cut fruits, and decay-prone commodities	Reduces microbial off-odors, moldy/sour notes and spoilage-related volatiles; may introduce herbal, spicy, phenolic, medicinal, or resinous masking notes if release is excessive	[35,36,37,81,108,109,116,117,118,119,120,121,122,123,124,125,126,127,128,129,130,131,132]
Ethylene scavenging or adsorption	Mainly climacteric or ethylene-sensitive fruits	Sachets, pads, papers, cardboard inserts, zeolites, activated carbon, or mineral supports	Ethylene adsorption, oxidation, or scavenging from the headspace	Apple, banana, pear, peach, tomato, mango, and other ethylene-sensitive commodities	Delays senescence and softening; excessive ethylene removal may weaken ester, lactone, and ripe-fruit aroma formation	[155,156,157,158,159,160,161,162,163,164,165,166,167]
Suppression of ethylene biosynthesis by released volatiles	Climacteric or ethylene-sensitive produce; effect must be adjusted to maturity stage	Active paper sheets, active cardboard, and essential-oil-releasing systems	Controlled emission of aroma-active compounds that reduce ACC synthase/ACC oxidase activity or ethylene production	Apple, tomato, broccoli, citrus, blueberries, blackberries, and flat peaches	Combines anti-senescence and antimicrobial effects; released volatiles may reinforce or distort the native aroma depending on dose and compatibility	[33,81,172,173,174,175,176,177,178,179,180]
SO_2_-emitting systems	Non-climacteric, decay-prone fruits	Cellulosic pads combined with perforated liners or boxes	Humidity- and temperature-dependent release from sodium/potassium metabisulfite	Table grapes	Highly effective against Botrytis and moldy/rotten off-odors; excessive release causes bleaching, sulfurous off-flavor, tissue injury, and residue concerns	[34,35,36,37,38,40,41,42,43,44]
Ethanol emitters	Non-climacteric or decay-prone commodities where fungal control is the main objective	Pads, sachets, or emitter systems	Controlled ethanol vapor release	Table grapes and selected decay-prone fruits	Suppresses fungal development and decay-related off-odors; excessive vapor may cause alcoholic, fermented, or overripe perception	[39,133]
O_2_ scavengers	Fruit-specific; useful only when the lower oxygen limit is respected	Sachets, labels, pads, or scavenging layers	Chemical or enzymatic oxygen removal	Products sensitive to oxidation or requiring low residual O_2_	Can reduce oxidative reactions and stabilize atmosphere; over-scavenging promotes anaerobic metabolism, and fermentative off-flavors	[106,107,110,111,113,114,115,181]
CO_2_ emitters or absorbers	Fruits where CO_2_ control must be actively buffered or maintained	Sachets, pads, active layers, or gas-regulating components	CO_2_ release, absorption, or buffering	Berries, strawberries, and packaged high-respiring commodities	May support microbial inhibition or prevent CO_2_ excess; mismatch can cause CO_2_ injury, sour notes, and altered microbial ecology	[106,107,110,112,113,114,115,181]
Moisture absorbers and humidity regulators	Berries, fresh-cut fruits, high-transpiration fruits, and packages prone to condensation	Hygroscopic cardboard, absorbent pads, trays, and humidity-buffering layers	Water-vapor adsorption, liquid uptake, or humidity buffering	Berries, fresh-cut fruit mixes, soft fruits, and ready-to-eat products	Reduces condensation-driven microbial volatiles and surface wetness; excessive drying reduces juiciness, freshness, and aroma release	[134,135,136,137,138,139,140,141,142]
Multi-active systems	Mixed physiological categories; especially complex deterioration scenarios and long supply chains	Active cardboard, multilayer films, combined pads/sachets, and integrated systems	Combined gas regulation, antimicrobial release, ethylene control, moisture buffering, and material selection	Fruits exposed to multiple deterioration pathways, or fluctuating cold chains	Potentially comprehensive protection; highest risk of over-control, incompatible mechanisms, masking, dryness, or consumer rejection	[139,140,141,142]
Packaging-adjacent edible coatings	Surface-level systems; fresh-cut fruits, citrus, and products requiring direct surface protection	Chitosan, waxes, biopolymer coatings, and surface-active layers	Surface gas-barrier effect, moisture control, and controlled release at the fruit surface	Citrus, fresh-cut melon, fresh-cut products, and coating-treated fruits	Can reduce surface deterioration and active release needs; may affect mouthfeel, texture, active-compound residue, and consumer acceptance	[23,24,40,41,42,43,44,104,105,146,147,148,149,150]

**Table 9:** Packaging material variables affecting MAP performance and fruit flavor preservation.

Material Variable	Mechanistic Effect	Flavor-Preservation Implication	Minimum Reporting Requirement	References
Film thickness and gas permeability	Controls O_2_/CO_2_ exchange and development of equilibrium atmosphere	Wrong permeability may trigger hypoxia, CO_2_ injury, or insufficient respiration control	Polymer type, thickness, OTR, CO_2_TR, perforation, headspace, and product load	[75,76,182,183,184,185]
Water-vapor transmission and hygroscopicity	Controls humidity, condensation, and surface wetness	Poor humidity control favors microbial off-odors or dehydration-related flavor weakening	WVTR, RH, condensation score, absorbent capacity, and storage temperature	[12,75,135,136,137,138]
Polymer polarity/crystallinity/free volume	Determines sorption and diffusion of aroma compounds	Selective scalping may reduce esters, terpenes or lactones, and distort varietal aroma	Polymer grade, crystallinity, partition/solubility/diffusion data, or headspace recovery test	[27,28,29,30,31,32]
Cellulosic paper/cardboard	Buffers moisture and can carry active compounds	May reduce condensation and release active volatiles with lower aroma scalping than some polymers	Material basis, active loading, release kinetics, and humidity response	[33,81,139,140,141,142,178,179,180]

**Table 10:** Indicative O_2_/CO_2_ flavor-risk matrix for packaged fruits.

Commodity/Context	Indicative Atmosphere Concern	Flavor Risk if Limit Is Exceeded	Recommended Monitoring	References
High-respiring fresh-cut fruit	Rapid O_2_ depletion and CO_2_ accumulation	Ethanol, acetaldehyde, ethyl acetate, and weak fruity aroma	Dynamic O_2_/CO_2_, respiration, ethanol, acetaldehyde, and sensory descriptors	[75,76,111,112]
Strawberry and berries	CO_2_ can delay decay but also modify ester profile	Suppressed typical fruity esters or increased fermentative esters	O_2_/CO_2_, fruity esters, ethyl esters, decay, and consumer liking	[12,106,107,110,113,114,115]
Climacteric fruits	Excessive O_2_ restriction or ethylene suppression	Weak ripe-fruit esters, lactones or delayed aroma development	O_2_/CO_2_, ethylene, maturity, esters, and liking	[51,52,168,169,170,171,172,173,174,175]
Table grapes	Atmosphere contributes to decay control but SO_2_/ethanol are key	Fermented, sulfurous, or residue-related defects if emitters are overused	SO_2_/ethanol residue, decay, off-flavor, and regulatory compliance	[34,35,36,37,38,39,133]

**Table 11:** Safety, migration, and regulatory checkpoints for flavor-oriented active packaging of fresh fruits.

Active-Packaging System	Main Safety or Migration Issue	Flavor/Sensory Relevance	Minimum Recommended Reporting	References
Essential-oil films, labels, papers, or cardboard	Migration or residue of aroma-active terpenes, phenols, aldehydes, or other volatile constituents; carrier safety	May preserve aroma by reducing spoilage, but may also mask native fruit volatiles or add herbal, spicy, medicinal, or resinous notes	Active identity, purity, loading dose, carrier, release kinetics, headspace concentration, residue/migration data, sensory threshold, and food-contact status	[40,41,42,43,44,81,108,109,116,117,118,119,120,121,122,123,124,125,126,127,128,129,130,131,132]
SO_2_-generating pads	Sulfite residues, consumer sensitivity, and excessive release under high humidity or temperature fluctuations	Suppresses moldy and rotten off-odors, but excess exposure may cause bleaching and sulfurous off-flavor	Pad formulation, release phase, liner type, humidity, temperature, SO_2_ residue, sensory descriptors, and target-market compliance	[34,35,36,37,38,39,40,41,42,43,44]
Ethanol emitters	Ethanol residue, vapor concentration, and possible alcoholic note	Controls decay but may reinforce fermented or overripe perception if excessive	Emission rate, headspace ethanol, fruit residue, off-flavor markers, and consumer acceptance	[39,40,41,42,43,44,133]
O_2_ scavengers and CO_2_ emitters/absorbers	Over-scavenging, excessive CO_2_, reactive components, or sachet integrity	May reduce oxidation or decay, but may induce anaerobic metabolism, ethanol, acetaldehyde, and ethyl acetate	O_2_/CO_2_ kinetics, lower oxygen limit, fruit tolerance, sachet composition, placement, and integrity	[40,41,42,43,44,106,107,110,111,112,113,114,115,181]
Ethylene scavengers or adsorbents	Chemical oxidants, mineral supports, nanoparticles, or adsorbent migration if not immobilized	Delays senescence, but excessive ethylene control may suppress ripening-related aroma in climacteric fruits	Active chemistry, immobilization, sachet/carrier integrity, ethylene kinetics, fruit maturity, and aroma outcomes	[33,40,41,42,43,44,155,156,157,158,159,160,161,162,163,164,165,166,167,168,169,170,171,172,173,174,175,176,177,178,179,180]
Moisture absorbers, pads and trays	Contact safety of absorbent materials, leachates, antimicrobial additives, or pad rupture	Reduces condensation and microbial volatiles, but excessive drying may reduce freshness, and aroma release	Absorbent composition, contact status, water uptake, leachate assessment, humidity profile, and texture/sensory data	[40,41,42,43,44,134,135,136,137,138,139,140,141,142]
Polymeric, coated or multilayer films	Overall and specific migration, additives, adhesives, inks, non-intentionally added substances, and active-agent release	May scalp esters/terpenes or release compounds contributing to off-odors	Polymer type, layer structure, food-contact side, migration data, OTR/CO_2_TR/WVTR, additives, and aroma-scalping evaluation	[27,28,29,30,31,32,40,41,42,43,44]
Edible coatings and surface-active layers	Direct consumption of coating constituents and active compounds	May modify gas exchange and preserve aroma, but may also coat the palate, modify texture, or introduce active-compound taste	Composition, dose per surface area, active release, residue, sensory descriptors, regulatory status, and consumer acceptance	[23,24,40,41,42,43,44,104,105,146,147,148,149,150]

**Table 12:** Critical summary of potential negative effects of active packaging on fruit taste and aroma.

Active-Packaging Strategy	Potential Negative Effect	Main Sensory Consequence	Control Parameter Required	References
Passive or active MAP /high-barrier films	Excessive O_2_ depletion or CO_2_ accumulation	Ethanol, acetaldehyde, ethyl acetate, alcoholic, solvent-like, or fermented notes	Commodity-specific O_2_/CO_2_ tolerance limits, respiration rate, package permeability, and temperature history	[12,14,17,75,76,111,112]
O_2_ scavengers	Over-scavenging and anaerobic metabolism	Fermentative off-flavors and suppression of normal aroma biosynthesis	Lower oxygen limit, scavenger capacity, product load, headspace volume, and storage temperature	[106,107,110,111,113,114,115,181]
CO_2_ emitters or absorbers	Excessive CO_2_ or inadequate CO_2_ buffering	CO_2_ injury, sour or fermented notes, and altered microbial ecology	Fruit CO_2_ tolerance, emitter/absorber kinetics, perforation, and gas monitoring	[106,107,110,112,113,114,115,181]
Ethylene scavengers	Excessive suppression of ripening-related processes	Weak aroma, reduced esters/lactones, and insufficient ripe-fruit identity in climacteric fruits	Fruit maturity stage, climacteric behavior, ethylene sensitivity, and target market window	[33,155,156,157,158,159,160,161,162,163,164,165,166,167,168,169,170,171,172,173,174,175,176,177,178,179,180]
Essential-oil films, labels, papers, or cardboard	Burst release or excessive headspace concentration of active volatiles	Herbal, spicy, medicinal, phenolic, resinous, or artificial notes; masking of native aroma	Release kinetics, active dose, headspace concentration, sensory threshold, and consumer acceptance	[35,36,37,81,108,109,116,117,118,119,120,121,122,123,124,125,126,127,128,129,130,131,132]
SO_2_-generating pads	Excessive release under high humidity or temperature fluctuation	Sulfurous off-flavor, bleaching, rachis damage, and residue concerns	Pad formulation, liner perforation, humidity, temperature, residue level, and target-market limits	[34,35,36,37,38,39,40,41,42,43,44]
Ethanol emitters	Excessive vapor concentration or residue	Alcoholic, fermented, or overripe perception	Emission rate, fruit residue, headspace ethanol, and sensory validation	[39,133]
Moisture absorbers and pads	Under- or over-control of humidity	Condensation-driven microbial odor if insufficient; dryness, loss of juiciness, and weaker aroma release if excessive	Relative humidity, condensation, water uptake, texture, and juiciness measurements	[134,135,136,137,138,139,140,141,142]
Polymeric films and multilayers	Aroma scalping and migration or odor from additives/NIAS	Loss of key esters, terpenes or aldehydes; package-related off-odors	Polymer type, thickness, crystallinity, partition/sorption data, migration testing, and headspace recovery assays	[27,28,29,30,31,32,40,41,42,43,44]
Multi-active systems	Combined over-control or incompatible mechanisms	Weak native aroma, exogenous notes, fermentation, dryness, or consumer rejection despite good visual shelf life	Integrated optimization of gas balance, moisture, active release, microbial control, volatilome, and sensory acceptance	[139,140,141,142,143,144]

**Table 13:** Comparative matrix of active-packaging technologies for preserving taste and aroma in fresh fruits.

Active-Packaging Strategy	Main Target Mechanism	Most Suitable Fruit/Context	Expected Effectiveness for Flavor Preservation	Main Limitation or Sensory Risk	Best Validation Endpoints	References
MAP and active gas regulation	Respiration control, O_2_/CO_2_ balance, reduction of senescence, and fermentative risk	High-respiring fruits, fresh-cut fruits, climacteric fruits requiring delayed ripening, berries under cold storage	High when atmosphere remains within commodity-specific tolerance limits; low or negative if hypoxia develops	Low O_2_ or high CO_2_ may induce ethanol, acetaldehyde, ethyl acetate, and weak aroma biosynthesis	O_2_/CO_2_ kinetics, respiration rate, ethanol, acetaldehyde, ethyl acetate, esters, sensory freshness, and off-flavor descriptors	[75,78,79,111,112]
Antimicrobial active packaging with essential oils or plant volatiles	Microbial growth and microbial off-odor suppression	Berries, citrus, fresh-cut fruits, decay-prone fruits, and short shelf-life commodities	Moderate to high when release is controlled and aroma-compatible; low if active aroma dominates	Herbal, spicy, medicinal, phenolic or resinous masking of native fruit aroma	Microbial counts, decay, active-compound headspace concentration, native aroma volatiles, trained-panel descriptors, and consumer acceptance	[35,36,37,81,108,109,116,117,118,119,120,121,122,123,124,125,126,127,128,129,130,131,132]
Ethylene scavengers or ethylene-regulating systems	Senescence delay and ethylene-mediated ripening control	Climacteric or ethylene-sensitive fruits; mature-firm fruits requiring delayed softening	High for delaying ripening and senescence; variable for aroma preservation	Excessive ethylene removal may suppress esters, lactones, and ripe-fruit aroma	Ethylene kinetics, firmness, maturity index, esters, lactones, ripening-related volatiles, and consumer liking	[33,155,156,157,158,159,160,161,162,163,164,165,166,167,168,169,170,171,172,173,174,175,176,177,178,179,180]
SO_2_-generating pads	Fungal decay control, especially Botrytis suppression	Non-climacteric decay-prone fruits, especially table grapes	Very high for decay control in grapes; flavor benefit is indirect through prevention of moldy/rotten notes	Sulfurous off-flavor, bleaching, rachis damage, residues, and sulfite sensitivity	Botrytis incidence, SO_2_ residue, bleaching, rachis browning, sulfurous descriptors, consumer acceptance and compliance	[34,35,36,37,38,39,40,41,42,43,44]
Ethanol emitters	Antimicrobial vapor action and decay suppression	Table grapes and selected decay-prone commodities	Moderate to high for decay control; flavor impact depends on dose and residue	Alcoholic, fermented, or overripe perception if excessive	Ethanol headspace/residue, decay incidence, fermentative volatiles, off-flavor descriptors, and liking	[39,133]
O_2_ scavengers	Reduction of oxidative reactions and control of package oxygen	Products sensitive to oxidation or requiring low residual oxygen; use must be fruit-specific	Conditional; beneficial if O_2_ remains above lower oxygen limit, negative if over-scavenging occurs	Anaerobic metabolism, ethanol/acetaldehyde accumulation, and aroma suppression	Lower oxygen limit, O_2_ kinetics, fermentative markers, esters, and sensory off-flavor detection	[106,107,110,111,113,114,115,181]
CO_2_ emitters or absorbers	CO_2_ control, microbial inhibition, or avoidance of CO_2_ injury	Fruits where CO_2_ level must be actively buffered or maintained	Conditional; depends strongly on fruit CO_2_ tolerance	CO_2_ injury, sour or fermented notes, altered microbial ecology	CO_2_ kinetics, fruit injury symptoms, microbial counts, ethanol/ethyl acetate, and sensory descriptors	[106,107,110,112,113,114,115,181]
Moisture absorbers, pads and humidity regulators	Condensation control, reduction of wet microzones, and microbial niches	Berries, fresh-cut fruits, high-transpiration fruits, and packages prone to condensation	Moderate as a stand-alone strategy; high as a supporting technology	Excessive drying may reduce juiciness, freshness, and aroma release	Relative humidity, condensation, weight loss, texture, juiciness, microbial counts, and aroma release	[134,135,136,137,138,139,140,141,142]
Material selection and aroma-scalping control	Retention of key odorants and avoidance of polymer-volatile sorption	Citrus, strawberry, peach, apple, melon and fruits rich in low-threshold esters, terpenes, or lactones	High as a design determinant; not a preservation technology alone	Poorly selected polymers may scalp esters, terpenes, or aldehydes despite good shelf-life performance	Polymer-volatile partitioning, headspace recovery, key odorants, OAVs, and sensory aroma intensity	[27,28,29,30,31,32]
Multi-active systems	Simultaneous control of gas balance, microbial growth, ethylene, moisture, and active release	Fruits with multiple deterioration pathways; fresh-cut products; long or fluctuating supply chains	Potentially high, but only if mechanisms are balanced and sensory-compatible	Highest risk of over-control, incompatible mechanisms, masking, hypoxia, or consumer rejection	Integrated gas, microbial, moisture, volatilome, active-release, sensory, and consumer-acceptance endpoints	[139,140,141,142,143,144]
Packaging-adjacent edible coatings	Surface gas-barrier effect, moisture control and active-compound release at fruit surface	Fresh-cut fruits, citrus and fruits requiring surface protection or controlled release	Conditional; useful when surface-level deterioration dominates	Coating taste, mouthfeel changes, active-compound residue, and regulatory/consumer acceptance concerns	Coating composition, dose, gas exchange, texture, mouthfeel, residue, sensory descriptors, and consumer acceptance	[23,24,40,41,42,43,44,104,105,146,147,148,149,150]

**Table 14:** Kinetic matching between active-packaging function, fruit physiology, and flavor preservation.

Active-Packaging Function	Physiological or Sensory Process to Match	Risk If Release/Scavenging Is Too Slow	Risk If Release/Scavenging Is Too Fast or Excessive	Minimum Kinetic Variables to Report	References
Essential-oil or plant-volatile release	Microbial growth rate, ethylene-related senescence, native aroma evolution, and sensory threshold of the active compound	Microbial spoilage, moldy or sour off-odors, insufficient senescence delay	Herbal, spicy, phenolic, medicinal, resinous, or citrus-like masking of native aroma; consumer rejection	Active-agent loading, release curve, headspace concentration, temperature, RH, package volume, product load, sensory threshold, and consumer acceptance	[33,81,108,109,116,117,118,119,120,121,122,123,124,125,126,127,128,129,130,131,132]
SO_2_ emission	Botrytis pressure, berry transpiration, package humidity, and cold-chain temperature	Gray mold development, rotten or moldy off-odors, rachis deterioration	Bleaching, rachis injury, sulfurous off-flavor, sulfite residues, and safety concerns	Pad formulation, release phase, SO_2_ concentration/residue, RH, temperature, liner perforation, decay, and sulfurous descriptors	[34,35,36,37,38,39,40,41,42,43,44]
Ethanol emission	Fungal growth rate and fruit tolerance to ethanol vapor/residue	Insufficient decay control and microbial off-odor formation	Alcoholic, fermented or overripe notes; residue-related acceptance problems	Emission rate, headspace ethanol, fruit residue, storage temperature, decay incidence, and fermented-note intensity	[39,133]
O_2_ scavenging	Fruit respiration rate, lower oxygen limit, and oxidative-reaction risk	Oxidation or inadequate control of residual oxygen	Hypoxia, ethanol, acetaldehyde, ethyl acetate, and suppression of desirable aroma biosynthesis	O_2_ depletion curve, respiration rate, lower oxygen limit, headspace volume, product load, temperature, and fermentative markers	[106,107,110,111,113,114,115,181]
CO_2_ emission or absorption	CO_2_ production, microbial inhibition needs, and fruit CO_2_ tolerance	Weak antimicrobial effect or insufficient atmosphere stabilization	CO_2_ injury, altered microbial ecology, sour or fermented notes	CO_2_ curve, fruit CO_2_ tolerance, emitter/absorber capacity, perforation, temperature, and injury/off-flavor descriptors	[106,107,110,112,113,114,115,181]
Ethylene scavenging or adsorption	Ethylene production, climacteric behavior, maturity stage and target market window	Senescence, softening and over-ripening continue unchecked	Suppression of ripening-related esters, lactones and ripe-fruit aroma; weak sensory identity	Ethylene curve, fruit maturity, respiration, firmness, esters, lactones, sensory ripeness and liking	[33,155,156,157,158,159,160,161,162,163,164,165,166,167,168,169,170,171,172,173,174,175,176,177,178,179,180]
Moisture absorption or humidity regulation	Fruit transpiration, condensation risk, microbial growth, and juiciness preservation	Condensation, surface wetness, microbial niches, and microbial volatile production	Excessive dehydration, lower juiciness, weaker aroma release, and dry mouthfeel	RH curve, condensation score, water uptake, weight loss, texture, juiciness, microbial counts, and aroma intensity	[134,135,136,137,138,139,140,141,142]
Multi-active systems	Combined respiration, ethylene, microbial, moisture, and active-release dynamics	One deterioration route remains uncontrolled despite other active functions	Over-control, incompatible mechanisms, hypoxia, masking, dryness, or sensory rejection	Integrated kinetic monitoring of gases, RH, active release, microbial counts, volatile markers, texture, and consumer acceptance	[139,140,141,142,143,144]

**Table 15:** Actionable future research agenda for flavor-oriented active packaging of fresh fruits.

Future-Research Priority	Specific Action Required	Minimum Variables or Methods to Report	Expected Output	References
1. Standardize reporting	Use a minimum reporting checklist	Species, cultivar, maturity, climacteric behavior, cut status, product load, headspace, material, thickness, permeability, dose, temperature, RH, and time	Comparable studies and identification of weak visual-only claims	[75,76,182,183,184,185]
2. Match kinetics with physiology	Quantify whether active component acts within physiological and sensory window	O_2_/CO_2_, ethylene, respiration, release/scavenging rate, temperature/RH dependence, and tolerance limits	Avoid hypoxia, CO_2_ injury, excessive ethylene suppression, burst release, and masking	[33,34,35,36,37,38,39,111,112,133]
3. Validate aroma with sensory-relevant endpoints	Move from total VOCs to odor-active compounds and off-flavor markers	GC-MS, GC-O, esters, aldehydes, terpenes, lactones, sulfur compounds, ethanol, acetaldehyde, ethyl acetate, thresholds, and OAVs	Flavor preservation evaluated by sensory relevance	[14,17,23,24,25,26]
4. Combine trained and consumer testing	Distinguish sensory mechanisms from market relevance	Descriptive analysis, hedonic liking, preference, purchase intention, CATA, JAR, blinding, serving temperature, and visual control	Packaging judged by perceived freshness, familiarity, liking, and acceptance	[45,46,47]
5. Validate realistic supply chains	Test under cold-chain fluctuation, retail display, and domestic handling	Temperature abuse cycles, humidity, condensation, vibration, stacking, microbial counts, visual quality, texture, volatilome, and consumer endpoints	Real performance assessed under conditions where off-flavors, and rejection occur	[75,76,80,88,89,90]

## Data Availability

No new data were created or analyzed in this study.

## Supplementary Materials

The following supporting information can be downloaded at: https://www.mdpi.com/article/10.3390/foods15183299/s1, Table S1: Methodological Quality Evaluation Form for Original Studies on Packaging-Induced Odor and Flavor Preservation in Fresh Fruits; Table S2: Material-Science Descriptors and Reported Sorption/Partition Evidence for Aroma Scalping in PP, PET and PLA; Table S3: Indicative Odor-Threshold Values, Sensory Descriptors, and Interpretation of Selected Aroma-Active Compounds Relevant to Active Packaging of Fresh Fruits.
