# Supplementary material for: Active Packaging, Fruit Volatilome, and the Preservation of Taste and Aroma Quality in Fresh Fruits: A Review
Source: Foods. 2026 Sep 17;15(18):3299. doi: 10.3390/foods15183299 (PMC13606839; doi:10.3390/foods15183299)
Supplement: Supplementary file 1 [file foods-15-03299-s001.zip › foods-4495919-supplementary.pdf]

Supplementary Materials

The following supplementary tables are included in this tracked working version for reviewer response and editorial decision. They can be retained as supplementary files or moved outside the main manuscript during final submission.

Supplementary Table S1. Methodological Quality Evaluation Form for original studies on packaging-induced odor and flavor preservation in fresh fruits.

Domain	Evaluation item	Score 0	Score 1	Score 2	References
Study focus	Clear packaging-flavor or packaging-induced odor objective	Not stated	Flavor mentioned indirectly	Explicit aroma, taste, volatilome or off-flavor objective	[200,201]
Fruit material	Species, cultivar, maturity stage, climacteric status and cut status	Poorly described	Partially described	Fully described and justified	[200,201]
Control treatment	Appropriate comparison against passive packaging, no active agent or commercial standard	No clear control	Control present but incomplete	Relevant control clearly defined	[200,201]
Packaging description	Material, thickness, O2/CO2 transmission, WVTR, perforation, headspace and product load	Poorly reported	Some variables reported	Complete packaging characterization	[200,201]
Active system	Active agent, dose, carrier, position, food-contact status and release/absorption kinetics	Poorly reported	Agent/dose reported but kinetics missing	Full active-system characterization	[200,201]
Storage scenario	Temperature, duration, humidity, retail simulation and cold-chain fluctuations	Simplified or incomplete	Basic storage conditions reported	Realistic and well-controlled storage scenario	[200,201]
Gas and humidity monitoring	O2, CO2, ethylene, relative humidity and condensation evolution	Not measured	Measured at limited points	Dynamic monitoring during storage	[200,201]
Volatilome analysis	VOC method, sampling, calibration, replicates, key volatiles and off-flavor markers	No volatile analysis	Total VOCs or limited markers	Targeted odor-active and off-flavor compounds included	[23–26,200,201]

Domain	Evaluation item	Score 0	Score 1	Score 2	References
Sensory validation	Descriptive analysis, consumer acceptance, blinding, serving temperature and panel details	No sensory validation	General sensory score only	Methodologically described sensory validation	[45–47]
Microbial context	Microbial counts, visible decay, spoilage organisms or microbial VOCs	Not assessed	Decay or counts only	Microbial data integrated with volatile interpretation	[23,82–87]
Statistical design	Biological replication, technical replication, randomization and statistical analysis	Weak or unclear	Partial reporting	Robust experimental and statistical design	[200,201]
Causal interpretation	Ability to distinguish physiology, microbial spoilage, aroma scalping and active-compound masking	Not discussed	Partially discussed	Mechanistic interpretation supported by data	[200,201]

Note: Each item is scored from 0 to 2. The total score ranges from 0 to 24. Studies scoring 20-24 are considered strong evidence for packaging-flavor interpretation, 14-19 moderate evidence and <=13 limited evidence. Studies without volatile analysis or sensory validation should not be used alone to claim flavor preservation, even when visual shelf life or decay control improves.

Supplementary Table S2. Material-science descriptors and reported sorption/partition evidence for aroma scalping in PP, PET and PLA.

Material	Representative aroma compounds	Reported coefficient or experimental approach	Main material-science interpretation	Relevance for fruit aroma preservation	References
PP / BOPP	Ethyl acetate, d-limonene	Sorption of individual and binary vapor mixtures assessed by gravimetric and thermal stripping/desorption methods; solubility coefficients followed Henry-type behavior in the studied range, with non-Fickian effects at higher limonene activity	Non-polar polyolefin structure can interact with hydrophobic terpene-like volatiles; film orientation and vapor activity influence diffusion and relaxation behavior	Relevant for citrus, mango, melon and other fruits rich in terpenes or fruity esters; PP films should be evaluated for selective loss of key odorants	[29]
PET / thermosealable polyester	Esters, alcohols, hydrocarbons,	Inverse gas chromatography and partition studies	Polyester polarity, chain packing and lower	PET may be preferable when aroma retention is	[30,31]

Material	Representative aroma compounds	Reported coefficient or experimental approach	Main material-science interpretation	Relevance for fruit aroma preservation	References
	ketones, limonene and ethyl caproate	showed selective sorption and comparatively lower scalping potential for PET than some polyolefin-based materials under tested conditions	compatibility with some non-polar volatiles may reduce sorption, but behavior depends on simulant, compound polarity and temperature	critical, but fruit-specific validation is needed because matrix effects can change partitioning	
PLA	Ethyl acetate and d-limonene	Diffusion, solubility and permeability coefficients were determined for ethyl acetate; d-limonene permeability was below detection under tested conditions	PLA behaves as a glassy polyester at room temperature and may limit permeation or scalping of selected aroma compounds, although crystallinity, grade and temperature are decisive	Promising for biodegradable flavor-oriented packaging, but must be tested under real fruit humidity, headspace and storage-temperature conditions	[32]
General polymer-volatile system	Esters, terpenes, aldehydes, alcohols and sulfur volatiles	Coefficients should be reported as K, S, D or P with compound, polymer grade, matrix/simulant, temperature, time and vapor activity specified	No universal coefficient applies to all esters, terpenes or polymer films; scalping is compound- and material-specific	Flavor-oriented active packaging should include targeted recovery or depletion tests for the key odor-active compounds of the fruit under study	[27–32]

Supplementary Table S3. Indicative odor-threshold values, sensory descriptors and interpretation of selected aroma-active compounds relevant to active packaging of fresh fruits.

Compound or compound group	Main sensory descriptor	Indicative odor-threshold example	Relevance for active-packaging studies	References
Ethyl butanoate	Fruity	1 microgram kg-1 reported in mango cv. Corazon	Key fruity ester; loss may indicate suppression of desirable aroma, while excessive ethyl esters together with ethanol/acetaldehyde may indicate fermentative imbalance	[25]
Ethyl 2-methylbutanoate	Fruity	0.2 microgram kg-1 reported in mango cv. Corazon	Relevant to apple, mango and tropical-fruit aroma; very low threshold means small changes may affect perceived fruitiness	[25]
Hexanal	Green, fatty	4.5 microgram kg-1 reported in mango cv. Corazon	Marker of green notes and lipid-oxidation/wound-related pathways; moderate levels may support freshness but excess suggests tissue damage	[25]
Nonanal	Floral, fatty or citrus-like depending on matrix	1 microgram kg-1 reported in mango cv. Corazon	Useful aldehyde marker for oxidative and aroma-balance changes; should be interpreted with other aldehydes and descriptors	[25]
Linalool	Floral, citrus-like	6 microgram kg-1 reported in mango cv. Corazon	Important in citrus, berries, mango and other fruits; may be preserved, lost by scalping or confused with released essential-oil volatiles	[25]
Limonene	Citrus-like	210 microgram kg-1 reported in mango cv. Corazon; thresholds may be much higher in complex citrus matrices than in water	Key citrus terpene; active packaging should distinguish native limonene retention from exogenous citrus-like volatile release or polymer scalping	[24,25]
Myrcene	Resinous, balsamic	36 microgram kg-1 reported in mango cv. Corazon	Relevant terpene in mango, citrus and tropical fruits; useful for assessing terpene	[25]

Compound or compound group	Main sensory descriptor	Indicative odor-threshold example	Relevance for active-packaging studies	References
			retention and material-related scalping	
gamma-Octalactone	Coconut-like, creamy	7 microgram kg-1 reported in mango cv. Corazon	Lactone associated with ripe-fruit notes; excessive ethylene suppression or immature harvest may reduce its contribution	[25]
gamma-Decalactone	Peach-like	11 microgram kg-1 reported in mango cv. Corazon	Important in peach-like aroma; packaging should preserve ripe-fruit lactones without promoting senescence or fermentation	[25]
beta-Ionone	Floral, violet-like	0.2 microgram kg-1 reported in mango cv. Corazon	Very odor-potent norisoprenoid; small concentration changes may have strong sensory effects	[25]
beta-Damascenone	Fruity, sweet	0.002 microgram kg-1 reported in mango cv. Corazon	Extremely low threshold; low-abundance volatiles can dominate aroma perception	[25]
(E,Z)-2,6-Nonadienal	Cucumber-like, green	0.01 microgram kg-1 reported in mango cv. Corazon	Important low-threshold aldehyde for fresh, green and cut-surface aroma interpretation	[25]
(E)-2-Nonenal	Fatty, oxidized	0.08 microgram kg-1 reported in mango cv. Corazon	Useful marker of oxidative or stale notes; may indicate loss of freshness even at low concentration	[25]
Ethanol, acetaldehyde and ethyl acetate	Alcoholic, sherry-like, solvent-like, fermented; fruity at low levels depending on matrix	Thresholds are highly matrix-dependent; fruit- or matrix-specific studies should be used whenever possible	Key fermentative/off-flavor markers in restrictive MAP, hypoxia or microbial activity; interpretation should combine concentration, O2/CO2, microbial counts and sensory descriptors	[17,24,26]
Sulfur-containing volatiles and essential-oil markers	Sulfurous, cabbage-like, rotten, onion/garlic-like; herbal, phenolic, spicy, clove-like, cinnamon-like or	Often very low or compound-specific thresholds; sensory threshold should be reported together with release kinetics	Relevant for SO2 systems, microbial spoilage and active-agent masking; even trace levels may affect consumer acceptance	[22,26,34–39,103]

Compound or compound group	Main sensory descriptor	Indicative odor-threshold example	Relevance for active-packaging studies	References
	citrus-like for essential-oil compounds			

Note for Supplementary Table S3: Values are indicative examples and should not be interpreted as universal thresholds. Odor thresholds depend strongly on matrix composition, pH, sugar/acid balance, temperature, headspace conditions, sensory method and route of perception. Whenever possible, active-packaging studies should report compound concentrations together with odor thresholds measured in the same or closely related fruit matrix, or should calculate odor activity values using clearly stated threshold sources.
